# Multiparameter Analysis of Human Bone Marrow Stromal Cells Identifies Distinct Immunomodulatory and Differentiation-Competent Subtypes

**DOI:** 10.1016/j.stemcr.2015.05.005

**Published:** 2015-06-09

**Authors:** Sally James, James Fox, Farinaz Afsari, Jennifer Lee, Sally Clough, Charlotte Knight, James Ashmore, Peter Ashton, Olivier Preham, Martin Hoogduijn, Raquel De Almeida Rocha Ponzoni, Y. Hancock, Mark Coles, Paul Genever

**Affiliations:** 1 Department of Biology, University of York, York YO10 5DD, UK; 2 Erasmus Medical Centre, Dr. Molewaterplein 50, Rotterdam 3015 GE, the Netherlands; 3 Department of Physics, University of York, York YO10 5DD, UK; 4 York Centre for Complex Systems Analysis, University of York, York YO10 5GE, UK

## Abstract

Bone marrow stromal cells (BMSCs, also called bone-marrow-derived mesenchymal stromal cells) provide hematopoietic support and immunoregulation and contain a stem cell fraction capable of skeletogenic differentiation. We used immortalized human BMSC clonal lines for multi-level analysis of functional markers for BMSC subsets. All clones expressed typical BMSC cell-surface antigens; however, clones with trilineage differentiation capacity exhibited enhanced vascular interaction gene sets, whereas non-differentiating clones were uniquely CD317 positive with significantly enriched immunomodulatory transcriptional networks and high IL-7 production. IL-7 lineage tracing and CD317 immunolocalization confirmed the existence of a rare non-differentiating BMSC subtype, distinct from *Cxcl12*-DsRed^+^ perivascular stromal cells in vivo. Colony-forming CD317^+^ IL-7^hi^ cells, identified at ∼1%–3% frequency in heterogeneous human BMSC fractions, were found to have the same biomolecular profile as non-differentiating BMSC clones using Raman spectroscopy. Distinct functional identities can be assigned to BMSC subpopulations, which are likely to have specific roles in immune control, lymphopoiesis, and bone homeostasis.

## Highlights

• Immortalized BMSC clones were generated for in-depth functional and biophysical analysis
• Distinct differentiation-competent and incompetent BMSC subsets are defined
• Trilineage-competent BMSC clones exhibit enhanced vascular interaction gene sets
• A non-differentiating, “immune-primed,” CD317^+^/IL-7^hi^ BMSC subset was identified in vivo

## Teaser

Genever and colleagues used immortalized human bone marrow stromal cell (BMSC) clones for in-depth analysis of subtype variation. They identified a rare, colony-forming BMSC subtype that lacked typical trilineage differentiation capacity, which under basal conditions displayed a marked immunomodulatory gene set, with high IL-7 and CD317 expression in vitro and in vivo.

## Introduction

Bone marrow stromal cells (BMSCs, also called bone-marrow-derived mesenchymal stromal cells) are heterogeneous populations that likely contain varying levels of tripotent (osteogenic, adipogenic, and chondrogenic [OAC]) stem-cell-like cells; cells with restricted potency (bi-, uni-, and nullipotent), committed precursors, and other stromal cell types. Phenotypic variations probably reflect in vivo functional diversity and a biological requirement for distinct stromal subsets with specific roles in bone marrow maintenance. Single-cell-derived BMSC clone analysis has identified considerable variation in differentiation capacity, ranging from OAC tripotency to nullipotency in vitro (Muraglia et al., 2000; Okamoto et al., 2002; Russell et al., 2010, 2011) and in vivo (Kuznetsov et al., 1997), which may indicate the existence of BMSC subtypes with varied potencies and/or a hierarchical developmental progression. BMSCs also possess significant immunomodulatory characteristics and can influence all aspects of immune cell function via cell-cell interaction and immunoregulatory factor secretion (Nauta and Fibbe, 2007), although clear demarcation between the skeletogenic and immunomodulatory capacity of BMSCs has not been made. Identification of human BMSCs often relies on non-discriminatory epitope detection (such as CD29, CD44, CD73, CD90 [THY-1], CD105, CD106 [VCAM-1], and CD166) with lack of hematopoietic markers (such as CD34, CD14, and CD45) (Dominici et al., 2006). Additional BMSC surface antigens have been described, including STRO-1 (Simmons and Torok-Storb, 1991), CD146 (MCAM) (Sacchetti et al., 2007), CD271 (LNGFR) (Quirici et al., 2002), Nestin (Méndez-Ferrer et al., 2010), platelet-derived growth factor receptor alpha (PDGFRα)/CD51 (Pinho et al., 2013), LNGFR^+^THY-1^+^VCAM-1^hi+^ (Mabuchi et al., 2013), and in mice, leptin receptor (LepR/CD295) (Zhou et al., 2014). However, cell-sorting experiments using these markers all show that they contain the colony-forming, differentiation-competent BMSC population, demonstrating that other, as yet undefined BMSC subtypes exist and further resolution of BMSC heterogeneity is required. In vitro studies of BMSC functionality are hindered by their limited lifespan as replicative senescence occurs during culture, thus limiting the number and depth of studies that can be performed. To address these issues, we immortalized human BMSCs using human telomerase reverse transcriptase (hTERT), followed by clonal isolation to generate a panel of BMSC lines (hTERT-BMSCs). This strategy enabled in-depth, multiparameter analysis of variation in human BMSC subpopulations with different behavioral traits that subsequently could be examined in heterogeneous primary BMSCs. We unveil a range of biophysico-chemical markers for identification of different BMSC subsets with specific immunomodulatory and differentiation competencies.

## Results

### Generation and Phenotyping of Variant hTERT Immortalized Clonal Cell Lines from Primary BMSCs

**Figure 1:**
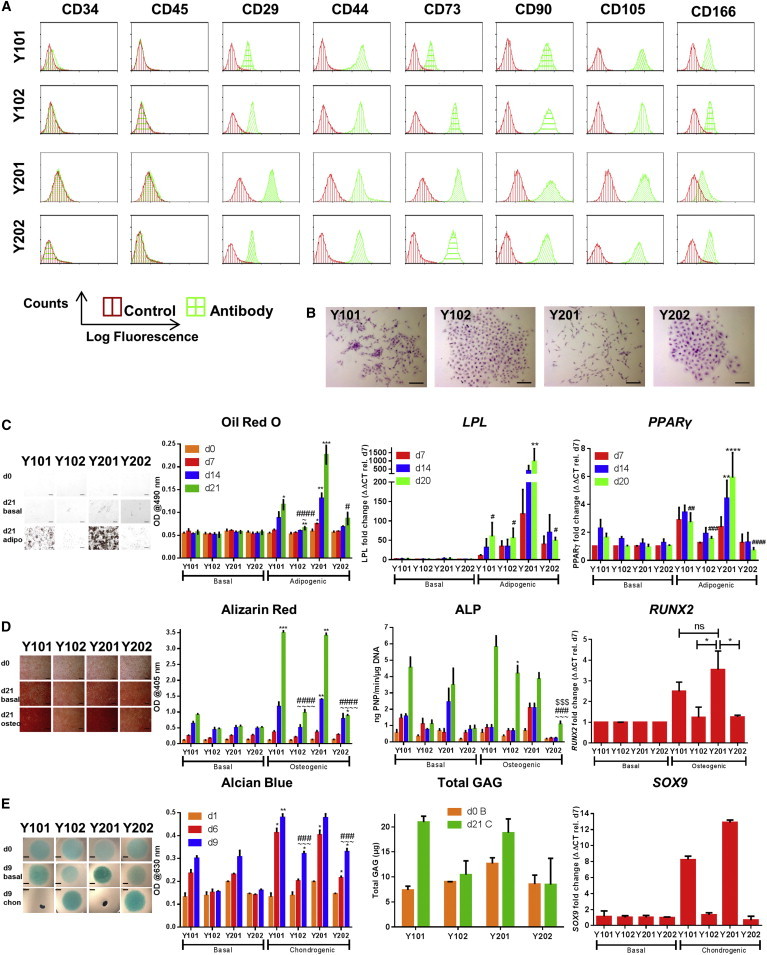
Generation and Analysis of hTERT-BMSC Clones (A) Analysis of hTERT-BMSC clones (Y101, Y102, Y201, and Y202) by flow cytometry for surface markers typically assigned to BMSCs. Representative histograms are shown. (B) Clonal growth of hTERT-BMSCs (crystal violet stain). Scale bars, 200 μm. (C–E) Assessment of the OAC potential of hTERT-BMSC clones; representative images and quantification of cells differentiated with adipogenic (C), osteogenic (D), and chondrogenic supplements (E). Scale bar represents 200 μm (C and D) or 2 mm (E). Data represent average quantified values ± SD for two or three independent experiments performed using two to six replicates. Differentiation was considered to be statistically significant after the data from n > 3 experiments were analyzed by one-way ANOVA with Holm Sidak’s multiple comparisons if ^∗^p < 0.05, ^∗∗^p < 0.01, ^∗∗∗^p < 0.001, or ^∗∗∗∗^p < 0.0001 between both day 0 and the time-matched basal control. Additionally, one-way ANOVA with Holm Sidak’s comparisons was performed between the different cell lines at day 21, and significant differences compared with Y101 (∼) or Y102 ($) and Y201 (#) are indicated. See also Figure S1.

A heterogeneous population of primary bone marrow BMSCs was isolated from one donor (FH181) based on their strong growth properties and multilineage differentiation potential. A single donor was deliberately selected to guard against expected genetic/lifestyle factors that cause inter-donor variation and would cloud interpretation of BMSC sub-population variation. A lentiviral expression system was used to overexpress hTERT in these cells, and stable-expressing lines were generated from single-cell-derived colonies. From numerous hTERT-BMSCs generated, our attention focused initially on four clonal lines selected for their strong clonal and stable growth characteristics (termed Y101, Y102, Y201, and Y202) that were maintained for over 400 days, showing exponential growth (Figure S1A) and elevated telomerase activity (Figure S1B). Their BMSC marker profile was determined by flow cytometry; all were positive for CD29, CD44, CD73, CD90, CD105, and CD166 and negative for CD34 and CD45 (Figure 1A), a profile matching their parental cells and typical of human primary BMSCs. All clones were also negative for CD11b, CD14, CD169, CD1a, CD4, CD11c, and CD83 (data not shown). It has been suggested previously that some BMSC lines may undergo oncogenic transformation either through hTERT transduction or extended time in culture (Serakinci et al., 2004). We found no evidence of tumorigenicity in our hTERT-BMSCs using in vitro colony transformation assays for non-adherent growth (Figure S1C) or following subcutaneous injection into immunocompromised mice (data not shown). Distinct differences were observed in individual cell morphologies and single-cell-derived colonies: Y101 and Y201 shared characteristics that were distinct from Y102 and Y202 lines. Y101 and Y201 lines had elongated fibroblastoid morphology, typical of BMSCs, whereas Y102 and Y202 were flattened and spread. When plated at clonal density, Y101/Y201 cells consistently formed dispersed colonies with low cell contact, characteristic of a migratory phenotype, whereas Y102 and Y202 cells formed regular, high-density, compact colonies (Figures 1B and S1D). Morphological differences were quantified by image analysis, which demonstrated that Y202 cells had a statistically significantly greater area and perimeter compared to both the Y101 and Y201 cells. Y102 cells were also larger than the Y101/Y201; the mean cell perimeters for Y102, Y202, Y101, and Y201 were 273 ± 23, 317 ± 107, 202 ± 19, and 190 ± 34 μm, and mean cell areas were 1,537 ± 132, 1,649 ± 614, 1,067 ± 92, and 857 ± 214 μm^2^, respectively (Figures S1E and S1F).

We identified variations in potency following OAC differentiation assays (Figures 1C–1E). Adipogenic media for up to 21 days induced the most potent observable and statistically significant adipogenic differentiation in Y201 cells at each time point, measured using the oil red O lipid-staining assay (Figure 1C). Y101 was also observed to differentiate into adipocytes, reaching significance at day 21, but Y102 and Y202 showed severely limited, although not completely absent, adipogenic potential, which was significantly different to Y201 at day 21. In addition, there were statistically significant increases in the levels of expression of the adipogenic genes peroxisome proliferator-activated receptor (PPAR)-γ and lipoprotein lipase (LPL) in the Y201 cells cultured in adipogenic media at day 14 and day 21 compared to the time-matched untreated controls. At day 21, the expression level of both these genes was significantly lower in the three other cell lines (Figure 1C). Y101 and Y201 differentiated convincingly along osteogenic lineages and showed elevated alkaline phosphatase (ALP) activity with significantly increased calcium deposition by alizarin red staining (Figure 1D), which was statistically higher than Y102/Y202 clones, which showed minimal mineral deposition, although Y102 displayed elevated ALP activity (Figure 1D). Increases in ALP activity observed under basal conditions for Y101 and Y201 were replicated in qPCR analyses of the ALP gene (Figure S1G); further increases in ALP gene expression were clearly evident in the Y101 cell line under osteogenic conditions, while the levels in the other lines only showed modest increases. Furthermore, histological staining for elevated ALP activity was only observable in the Y101/Y201 cells (Figure S1G). Gene expression analyses for *RUNX2* showed that only the Y101/Y201 cell lines upregulated this early osteogenic marker at day 7 (Figure 1D). In response to chondrogenic induction using transforming growth factor β (TGF-β), only lines Y101 and Y201 displayed an early cell-condensation phenotype associated with chondrogenic induction (Johnson et al., 2012), whereas no condensation was observed in similar micromass culture of lines Y102 and Y202 (Figure 1E). When cultures were stained with alcian blue and stain-associated glycosaminoglycans (GAGs) were eluted and quantified, significantly different levels were observed in all cell lines at day 9 compared to their time-matched controls under basal conditions, but the highest and statistically significant GAG levels were clearly evident in the Y101/Y201 compared to the Y102/Y202 cell lines. Similarly, only the Y101 and Y201 lines displayed marked increases in total GAG production under chondrogenic conditions in the Blyscan GAG assay (Figure 1E). Gene expression analyses for the early chondrogenic marker *SOX9* confirmed that this marker only increased in the Y101/Y201 cell lines after 7 days in chondrogenic differentiation media (Figure 1E). Therefore, lines Y101/Y201 were capable of BMSC OAC differentiation, with Y201 showing stronger adipogenesis. The Y102/Y202 BMSC lines exhibited a non-hematopoietic, stromal phenotype with distinctive clonal behavior but with atypical BMSC morphology and limited differentiation ability compared to Y101/Y201.

### Interrogation of Gene Expression Profiles Identifies Immune-Related BMSC Subtypes

**Figure 2:**
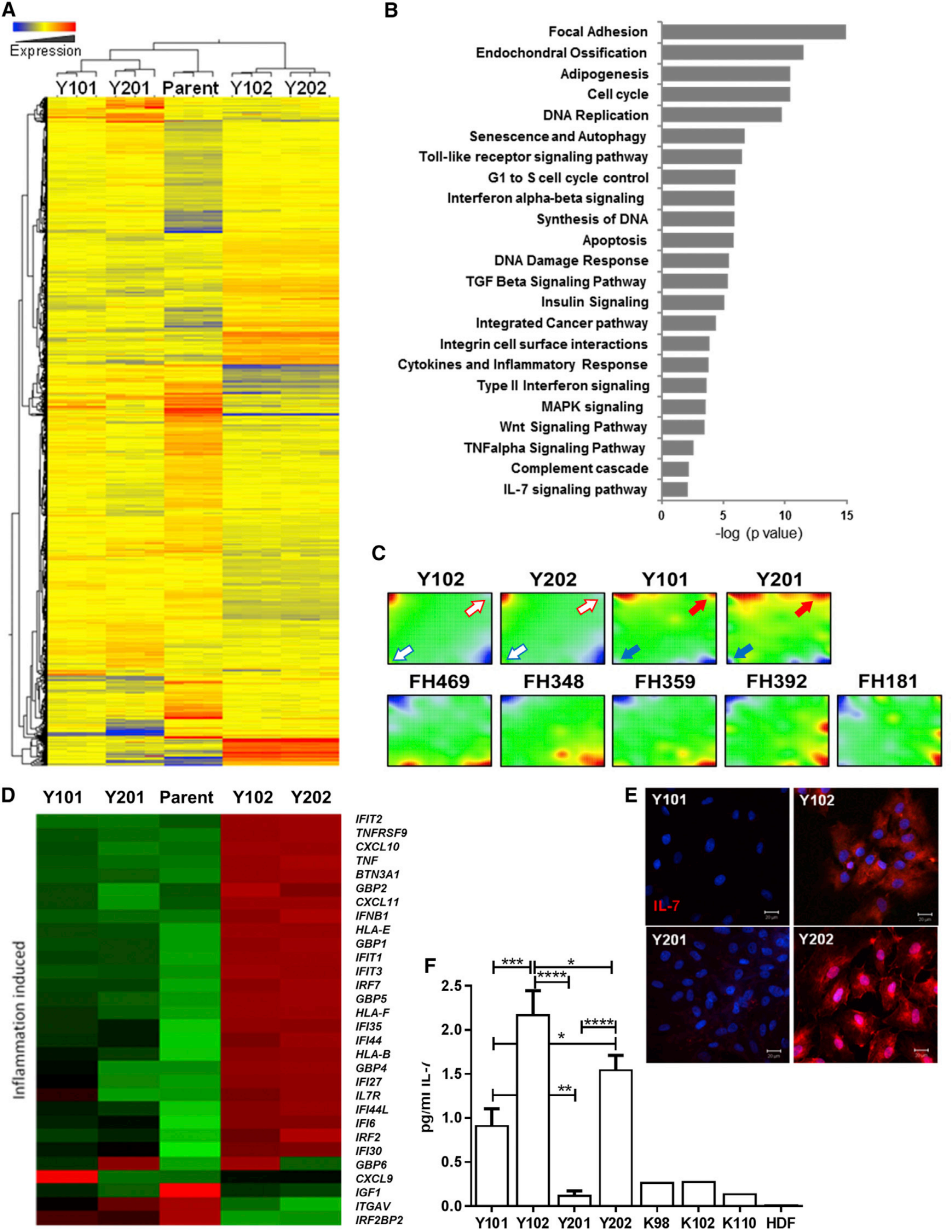
Expression Profiling Identifies BMSC Clones with Distinct Immunoregulatory Features (A) Hierarchical clustering heatmap of global gene expression profiles from each hTERT-BMSC clonal line and the parental BMSCs. (B) Pathway analysis showing most significantly enriched pathways. (C) Self-organizing heatmaps of the four hTERT-BMSC clones and five primary BMSC samples, including the parent population (FH181). Arrows indicate presence (closed) and absence (open) of overrepresented (red) and underrepresented (blue) metagene spots. (D) Heatmap showing expression of key genes involved in inflammatory-induced responses. (E) Immunocytochemical detection of IL-7 expression (red) by the four hTERT-BMSC clones (after 24 hr in culture) (DAPI nuclear counterstain in blue). Scale bars, 20 μm. (F) IL-7 expression by ELISA in the four hTERT-BMSC clones, primary BMSCs (K98, K102, and K110), and human dermal fibroblasts (HDF). Data represent mean values ± SD from three individual experiments performed in duplicate. See also Figure S2.

We performed global gene expression analyses to identify distinguishing characteristics between the hTERT-BMSC clones and parental BMSCs; selecting significantly differentially expressed genes (p < 0.05, >2-fold change). Hierarchical clustering grouped Y101 with Y201 and closest to the primary parent BMSCs, while Y102/Y202 clustered separately (Figure 2A). Principal component analysis (PCA) revealed that 70% of the total variance was captured by the first two components (Figure S2A) and confirmed segregation of Y102/Y202 BMSC lines from the Y101/Y201 BMSC lines, the parent BMSC (FH181), and other primary BMSC populations (Figure S2B). Pathway analysis identified significant differences in expression of genes involved in the cell cycle, DNA replication, and other processes associated with cell replication, as would be predicted when comparing hTERT-immortalized lines with the parent sample (Figure 2B). Additional gene sets identified were involved in cell adhesion, endochondral ossification, and adipogenesis. Differentially expressed genes were notably enriched in pathways involved in Toll-like receptor, interferon, tumor necrosis factor α (TNF-α), interleukin-7 (IL-7) signaling, and inflammatory responses, implicating potential differences in the immunoregulatory properties between these lines (Figure 2B). Patterns of gene expression were also visualized and investigated using self-organizing heatmaps (SOMs). Expression data were used to produce mosaic fingerprints, and each mosaic tile represents metagenes that consist of mini-clusters of genes with similar expression placed in the same position across the mosaics (Figure 2C). Y101, Y201, Y102, and Y202 were compared against the primary parent BMSC source (FH181) and four others (FH469, FH348, FH359, and FH392). Primary BMSCs showed consistent spots of strong over- and under-expression in the bottom-right and top-left corners, respectively. Y101/Y201 and Y102/Y202 hTERT-BMSCs showed differences in patterns of expression at regions of over- and under-expression in the top right and bottom left of the SOMs, respectively (Figure 2C, arrows, and Figures S2C and S2D). The most significant gene sets overexpressed in Y101/201 versus Y102/202 were related to vascular growth (blood vessel remodelling, blood vessel development, artery morphogenesis, and patterning of blood vessels; Figure S2C). Under-expressed Y101/Y201 gene sets that were most significantly over-represented in Y102/Y202 lines were immunomodulatory (antigen processing, MHC class II proteins, T cell signaling, and responses to interferon; Figure S2D).

Further analysis found a strikingly elevated endogenous expression of inflammation-induced genes in the non-differentiating lines Y102 and Y202 compared to the OAC-differentiation-competent BMSC clones (Figure 2D). By analyzing gene expression changes reported in a previously published dataset, where global gene expression was studied in adipose-derived BMSCs with or without stimulation with inflammatory cytokines (interferon γ [IFN-γ], TNF-α, and IL-6) (Crop et al., 2010), we found that inflammation-induced genes were also elevated in unstimulated Y102 and Y202 cells compared to the Y101/Y201 and parental BMSCs (Figure 2D). These observations pointed to the existence of a non-differentiating, resident stromal cell subset with “unlicensed” immunoregulatory potential associated with a pro-inflammatory response. The immunomodulatory effects of BMSCs are normally reported to be related to immunosuppression. In assays of immunosuppressive function, we found that all hTERT-BMSC lines were similarly capable of inhibiting anti-CD3/anti-CD28 antibody-stimulated peripheral blood mononuclear cell (PBMC) proliferation with no significant differences of the inhibitory capacity of the cell lines at each BMSC:PBMC ratio used (Figure S2E). Similar increases in the expression of immunosuppressive factors (*IDO1*, *TGFB1*, and *CD274*) following exposure to TNF-α and IFN-γ were observed in all hTERT-BMSC lines, but *IL6* expression was significantly higher in the Y201 cells under TNF-α/IFN-γ stimulation (Figure S2F). Y102/Y202 cells were significantly more responsive to inflammatory cytokine-induced expression of factors involved in lymphocyte homing and development, CXCL10 and IL-7. In particular, basal mRNA expression of *IL-7* in Y102/Y202 cells was equivalent to expression levels in TNF-α/IFN-γ-stimulated Y101/Y201 cells (Figure S2F). IL-7 signaling was highlighted as a differentially regulated pathway (Figure 2B), and we identified strong expression of IL-7 in Y102/Y102 compared to low IL-7 levels in Y101/Y201 by immunocytochemistry (Figure 2E). Using ELISAs, we confirmed significantly higher secretion of IL-7 in Y102/202 versus Y101/Y201; heterogeneous primary BMSCs secreted low levels of IL-7, with negligible expression in human dermal fibroblasts (Figure 2F). No difference in IL-7 receptor expression level was observed by flow cytometry across the four cell lines (data not shown).

Together, these data indicate that OAC-competent BMSC clones expressed an extensive gene set consistent with vascular interaction; all BMSC lines showed broadly similar immunosuppressive features, while the poorly differentiating hTERT-BMSCs displayed a striking constitutive immunostimulatory expression profile and could be identified by elevated basal IL-7 expression, which has important functions in lymphopoiesis (Ceredig and Rolink, 2012).

### In Vivo Lineage Tracing of IL-7-Expressing Stromal Cells

**Figure 3:**
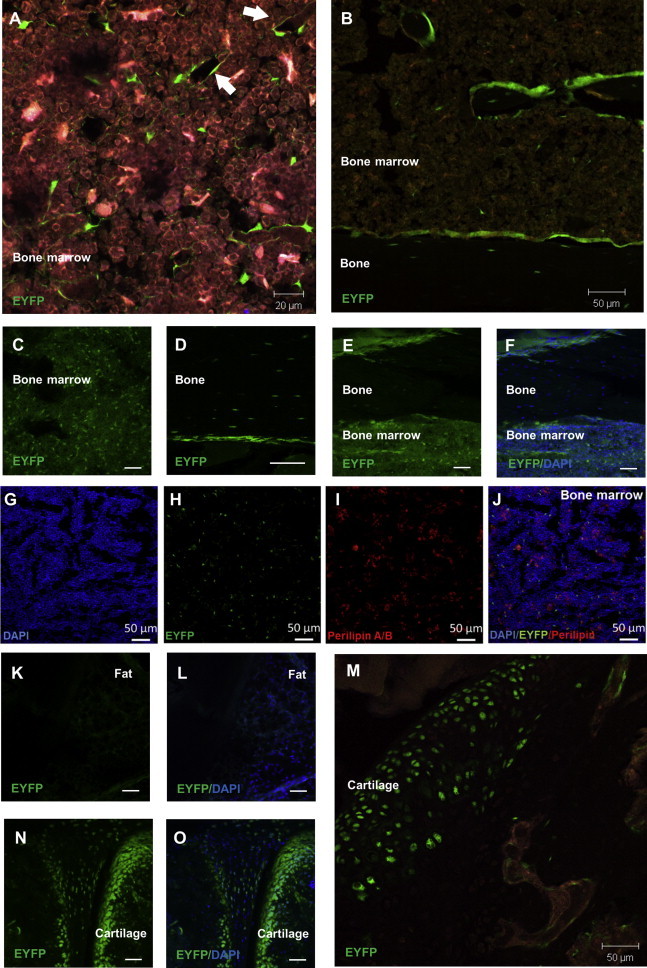
Lineage Tracing of IL-7-Expressing Cells Using IL-7cre Rosa26-EYFP Mice (A–F) Sporadic EYFP expression throughout bone marrow, associated occasionally with cells lining the vasculature (A, arrows) and prominent on endosteal surfaces (B). Infrequent osteocytes were EYFP positive (D–F). (G–J) Immunostaining of perilipin-positive adipocytes (red) in bone marrow of IL-7cre Rosa26-EYFP mice. (K and L) Absence of EYFP expression in white adipose tissue extracted from IL-7cre Rosa26-EYFP mice. (M–O) EYFP expression in chondrocytes of articular cartilage. Scale bars, 50 μm (unless otherwise stated). See also Figure S3.

To identify and track IL-7-positive BMSC subpopulations in vivo, we used an IL-7Cre Rosa26-EYFP lineage-tracing mouse model, where enhanced yellow fluorescent protein (EYFP) expression persists in the progeny of cells that expressed IL-7 at any stage. EYFP-positive cells were found in bone marrow occasionally at perivascular and endosteal-lining locations but broadly distributed throughout the marrow with an ∼4% frequency (Figures 3A–3F and S3A). Using flow cytometry to discriminate CD45-expressing hematopoietic cells, we found the EYFP-positive stromal fraction represented ∼0.9% of the marrow cell population (Figure S3B). From histological sections, 91%, 77%, and 92%, respectively, of femoral, tibial, and calvarial osteocytes, terminally differentiated cells of the osteogenic lineage (Figures 3D–3F and S3A), and all perilipin-positive bone marrow cells and all adipocytes in white adipose tissue examined were EYFP negative (Figures 3G–3L and S3C). Strong EYFP positivity was confirmed in 11%–60% of articular chondrocytes (Figures 3M–3O and S3A), where IL-7 expression has been previously identified (Long et al., 2008), and in 7%–31% of hypertrophic chondrocytes (Figure S3A). These data demonstrate that a substantial proportion of mesodermal-derived adipose, bone, and cartilage tissues originate from a progenitor cell type that at no point expressed IL-7 and support the existence of poorly/non-differentiating IL-7^hi^ BMSC subtype in vivo.

### Identification of Y102/Y202-Related Subtypes in Primary BMSCs

**Figure 4:**
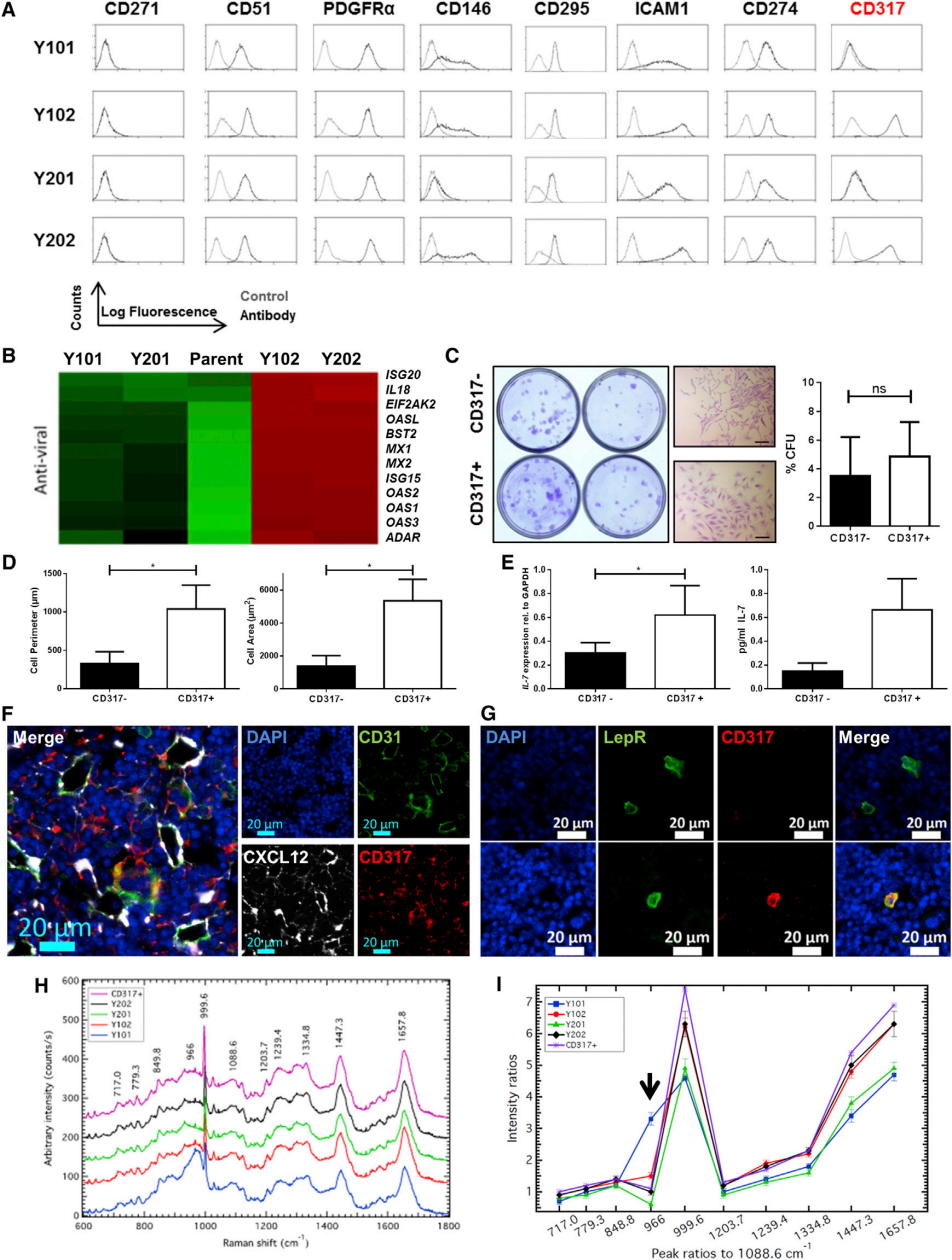
Identification of CD317^+^ Bone Marrow Cells (A) Flow cytometric analysis of hTERT-BMSC; CD317 expression selectively discriminates Y102/Y202 from Y101/Y201. (B) Heatmap showing expression of key genes involved in anti-viral responses. (C) CFU-F assay and morphology of CD317^−^ and CD317^+^ cells sorted from heterogeneous BMSCs. Scale bars, 200 μm. Histogram represents mean ± SEM (n = 4 independent experiments performed with five replicates). (D) Analysis of cell perimeter (left) and cell area (right) of CD317^−^ and CD317^+^ cells sorted from heterogeneous BMSCs. Mean ± SEM, n = 4 independent experiments in which, on average, 113 cells were examined. ^∗^p < 0.05 by unpaired non-parametric t test. (E) IL-7 mRNA (left) and protein expression (right, by ELISA) in CD317^−^ and CD317^+^ cells. Data represent mean IL-7 levels ± SD from FACS-sorted cells in triplicate from four or two donors, respectively. ^∗^p < 0.05 by paired t test. (F) Immunohistochemistry of mouse femur bone marrow sections stained with antibodies to CD317 (red), CD31 (green) with *Cxcl12*-DsRed (white) and nuclear (DAPI, blue) staining. Representative merged and single-panel images shown. (G) Immunolocalization of LepR/CD295 (green) and CD317 (red) in mouse bone marrow showing LepR^+^CD317^−^ (upper panel) and occasional dual LepR^+^CD317^+^ cells (lower panel). Blue indicates nuclear DAPI stain. (H and I) Analysis of hTERT-BMSC clones and CD317^+^ cells by Raman spectroscopy, Raman shifts (H) and peak ratios (I) are shown. Raman peak assignments are provided in Table S2. See also Figure S4.

Y101/Y201 and Y102/Y202 lines were screened by flow cytometry in an attempt to identify a distinguishing cell-surface protein to sort from primary BMSC populations. We found little difference in expression of candidate BMSC markers CD271, CD51, and PDGFRα; while CD146 was largely undetectable on the Y201 line, its expression was similar in the other BMSC clones (Figure 4A). LepR/CD295 was expressed by both OAC-differentiating lines and the differentiation-incompetent lines (Figure 4A); in murine bone marrow, LepR/CD295 expression was found to be predominantly, though not exclusively, expressed by bone- and adipocyte-forming BMSCs (Zhou et al., 2014).

We analyzed the array data for differential expression of genes encoding cell-surface proteins; while ICAM1 and CD274 had similar protein expression profiles across all lines, CD317 was selectively and highly expressed in Y102 and Y202 cells and absent from Y101 and Y201 cells (Figure 4A). We examined another four BMSC clones (Y204, Y205, Y301, and Y302) generated during the initial hTERT immortalization experiments (Figure S4A). They exhibited varying degrees of differentiation potency (Figures S4B–S4H), but none were differentiation incompetent across osteogenic, adipogenic, and chondrogenic lineages to the same degree as observed for Y102/Y202, and all were negative for CD317 (Figure S4I). CD317 (also known as BST2/tetherin) has recognized anti-viral properties (Hotter et al., 2013), and Y102/Y202 cells expressed elevated levels of several other anti-viral genes compared to Y101/Y201 and parent BMSCs (Figure 4B).

In low passage, primary BMSCs, a CD317-expressing population was detected in ∼1%–3% of the total cells (Figure S4J). We used fluorescence-activated cell sorting (FACS) to sort the CD317^+^ fraction from CD317^−^ cells for further analysis and found that CD317^+^ cells were capable of density-independent growth and formed CFU fibroblasts (CFU-F) at a frequency similar to CD317^−^ cells (Figure 4C). Morphological analysis of individual colonies identified that CD317^+^ cells had statistically significantly increased cell perimeters (1,042 ± 309 μm) and areas (5,355 ± 1,306 μm) compared to CD317^−^ cells (1,386 ± 638 μm and 330 ± 154 μm, respectively) (Figures 4D and S4K), mirroring Y102/Y202 versus Y102/Y201 size differences. Using qPCR and ELISA, we confirmed that CD317^+^ BMSCs expressed significantly elevated IL-7 mRNA and secreted more IL-7 protein than the CD317^−^ population (Figure 4E), with limited evidence that CD317^+^ cells have elevated basal mRNA expression levels of immunosuppressive factors compared to CD317^−^ cells (Figure S4L), while exposure of primary BMSCs to IFN-γ for 24 hr resulted in an ∼3-fold increase in CD317 expression (Figure S4M). As marker expression can change with time in culture, we also sorted CD317-expressing cells from fresh, whole human bone marrow mononuclear cells (BM-MNCs); CD317^+^ cells in the stromal CD45^−^CD31^−^ population represented on average 0.4% of the live cell population from two donors (Figures S4N and S4O).

In mouse tissue sections, CD317 was expressed sporadically throughout the bone marrow (Figures 4F and S4P; isotype control is shown in Figure S4Q). CD317-positive cells rarely associated with CD31-positive vascular cells (Figures 4F and S4P) and did not generally colocalize with *Cxcl12*-DsRed^+^ perivascular stromal and endothelial cells (Figure 4F). The majority of bone marrow LepR-expressing cells lacked CD317 expression, though occasional LepR^+^CD317^+^ cells were identified (Figure 4G).

Collectively, these findings identify the presence of a rare, colony-forming population of BMSCs, marked by CD317 positivity, which appear to have an enhanced immunostimulatory capacity and are closely related to the Y102 and Y202 phenotype. We employed Raman spectroscopy to determine the extent of this relationship and provide a diagnostic evaluation of functional variation between BMSC subtypes. Averaged spectra were generated for each hTERT clonal line and primary CD317^+^ sorted BMSCs (Figure 4H), with their respective peak assignments identified as described previously (Movasaghi et al., 2007; Schulze et al., 2010). Peak intensity ratios were obtained between all identified peaks for each cell type; the most discriminatory was found to be against the 1,088.6 cm^−1^ peaks. The 1,088.6 cm^−1^ peak is related to the symmetric phosphate stretch of the DNA backbone (Movasaghi et al., 2007) with its discrimination signifying a fundamental difference in the DNA of the cell types. This peak has also been used to discriminate other cell types, (Chan et al., 2006). The 966cm^-1^/1088.6 cm^−1^ peak intensity ratios distinguished Y101 from Y201 (Figure 4I, arrow). Other peak intensity ratios, most notably from 999.6 cm^−1^ and higher, clearly separated Y101/Y201 from Y102/Y202 (Figure 4I). Importantly, primary CD317^+^ cells mapped with remarkable consistency to the Y102/Y202 Raman profile (Figure 4I), providing strong evidence of relatedness.

## Discussion

We generated immortalized BMSC clones to perform detailed analysis of functional diversity in BMSC subtypes to levels not previously described. Similar immortalization techniques (e.g., hTERT combined with human papillomavirus E6/E7) have been used for the clonal analysis of BMSCs, which found OAC, AO, OC, unipotent, and nullipotent clones at varied frequencies, but other functional characteristics and discriminating features were not examined (Larsen et al., 2010; Okamoto et al., 2002). Notably, however, enhanced immune-related features could be assigned to non-bone-forming hTERT-BMSCs in a previous study (Larsen et al., 2010). Using non-immortalized BMSC clones, Muraglia et al. identified OAC-, OC-, and O-competent lines only, with very rare (<1%) nullipotent subsets (Muraglia et al., 2000). Rather than determine occurrence of O/A/C phenotypes in heterogeneous BMSCs, here our aim was to identify molecular and biophysical markers of BMSC subtypes and their alignment with non-progenitor cell functions, including immunomodulatory properties. To this end, from the original hTERT-BMSC lines generated, we selected four clones positioned at opposing ends of a differentiation competency scale, tripotent Y101/Y201 and relatively impotent Y102/Y202, for in-depth cell profiling. The OAC-differentiating lines expressed an extensive and significantly enriched gene set consistent with blood vessel support/interaction compared to nullipotent clones. This may reflect a relationship between BMSC differentiation competency and a perivascular niche location (Sacchetti et al., 2007). Conversely, Y102 and Y202 lines expressed CD antigens widely used to describe BMSCs (Dominici et al., 2006; Pittenger et al., 1999) but exhibited very little differentiation capacity. Subsequent assays revealed that an equivalent CD317^+^ cell type existed in heterogeneous primary BMSC populations at ∼1%–3% frequency and CD45^−^CD31^−^CD317^+^ cells represented ∼0.4% of whole human BM-MNCs. Primary cell analysis and in vivo validation is an important component of our study. Data obtained using in vitro immortalized cell lines must be interpreted with some caution, considering the effects of hTERT transduction artifacts. Integration site, enhanced telomerase activity and long-term culture are all likely to impact on cell function. We attempted to minimize potential confounding effects by selecting two cell lines, each displaying similar properties (Y101/Y201 and Y102/Y202). We used Raman spectroscopy, which has been demonstrated as a non-destructive label-free method to study the biological state of single cells and to identify molecular-scale differences for cell discrimination (see Schulze et al., 2010, for example). In this work, Raman spectroscopy was able to uniquely identify the four hTERT-BMSC clones, separating Y101 from Y201 and confirming a striking biomolecular relationship between Y102/Y202 and primary CD317^+^ cells, which also shared an enlarged, spread morphology, forming compact, non-migratory colonies. We performed detailed bioinformatics to scrutinize gene expression profiles against the parental BMSCs. These analyses ultimately identified the closely clustered Y102 and Y202 lines as having remarkably high basal level expression of factors associated with a pro-inflammatory and anti-viral function. The significantly enriched gene sets we identified in unchallenged Y102/Y202 BMSC lines are typical of an immediate immunostimulatory response to infection and other insults. Notably, all BMSC clones exhibited similar inhibitory effects on PBMC proliferation when activated by exposure to anti-CD3 and anti-CD28 to mimic antigen presentation. Consequently, Y102/Y202 BMSC subtypes may function in both immunostimulatory (early, pro-inflammatory) and immunosuppressive (anti-inflammatory, tissue protective) responses depending on the immune environment; however, functional validation and further refinement of the characteristics of this CD317-expressing population is required. Dual immunomodulatory functions for BMSCs have been identified and discussed (Dazzi and Krampera, 2011), but not assigned to BMSC subtypes. CD317^+^ cells (as well as Y102/Y202) were characterized as having elevated IL-7 expression, which has well-established roles in T and B cell development (Ceredig and Rolink, 2012). Examination of skeletal tissues in a lineage-tracing mouse model confirmed that a substantial subpopulation of the differentiated progeny of BMSCs could not have arisen from IL-7-expressing stromal progenitors. It should be noted that differentiated skeletal cells may express IL-7 (Long et al., 2008; Zhu et al., 2007), which would also be revealed as EYFP positivity. However, these studies provide unequivocal evidence linking IL-7 expression in stromal subsets with differentiation incompetence during normal development in vivo. Our in vivo observations were reflected in our in vitro assays of differentiation, where the IL-7^hi^ immortalized BMSC clones (Y102/Y202) exhibited limited skeletogenic potential. These in vitro assays are prone to artifact that could result in non-specific detection of widely used markers of differentiation that may not report authentic in vivo differentiation capacity, observed for example using in vivo transplantation techniques (Robey et al., 2014). Even with these caveats, we still failed to detect clear evidence of differentiation ability in Y102/Y202 CD317^+^ IL-7^hi^ BMSC lines. In vivo, CD317^+^ cells had a dispersed bone marrow distribution and did not generally colocalize with *Cxcl12*-DsRed^+^ perivascular stromal cells, again pointing to an alternative function for CD317^+^ BMSCs, unrelated to CXCL12-mediated hematopoietic stem cell niche support. CD317 has a well-defined anti-viral function acting to tether and restrict the release of viral particles and may also trigger the viral immune response (Hotter et al., 2013). The existence of a marrow-resident, non-migratory, non-differentiating colony-forming stromal cell type, apparently primed for host defense, is an interesting concept. These cells could act as first responders to pathogen invasion and/or provide low-level immune control. As IL-7 has been implicated as a causative factor in autoimmunity, including rheumatoid arthritis (Dooms, 2013), unregulated control of CD317^+^ cell numbers and function may also contribute to disease onset and progression. These findings highlight the importance of identifying unique markers of BMSC sub-populations and assigning function, which will instruct strategies to analyze stromal-immune cell interaction, differentiation competency, and cell selection for safe and effective therapy.

## Experimental Procedures

### Primary BMSC Isolation, Culture, and hTERT BMSC Production

All animal experiments and work involving human samples were approved by the University of York, Department of Biology ethics committee. Primary human BMSCs were isolated from femoral heads obtained with informed consent during routine hip replacement (see the Supplemental Experimental Procedures) or as explant cultures from human tibial plateaus after routine knee replacement. Cells were cultured in DMEM containing 15% fetal bovine serum, 100 U/ml penicillin, and 100 μg/ml streptomycin (changed every 3–4 days); cells were passaged at 70%–80% confluency. Cells were expanded and used between passage 1 (p1) and p5.

The hTERT lentiviral vector was produced using ViraPower Lentiviral Gateway Expression Kit (Invitrogen), according to the manufacturer’s guidelines, before transduction into primary human BMSCs isolated at p3 from the femoral head of a single donor. For selection of single cell lines, the transduced BMSCs were trypsinized and plated at 10 cells/cm^2^. Cells were grown in BMSC medium containing 20% HyClone serum at plating, then replaced with fresh media containing 15% HyClone serum every 3 or 4 days to day 14 (until discrete single-cell colonies were visible). Single-cell colonies were isolated and expanded.

For further information, see Supplemental Experimental Procedures.

## Author Contributions

S.J. and J.F. performed experiments and designed the studies. F.A., M.H., S.C., J.L., and C.K. performed in vitro experiments; J.A. and P.A. performed the bioinformatics analyses; and O.P., S.C., and M.C. provided and analysed in vivo data. R.D.A.R.P. and Y.H. designed and analysed the Raman work. P.G. supervised the work and designed experiments. All authors contributed to writing the paper, which was led by J.F., S.J., and P.G.

## Supplemental Information

Document S1. Supplemental Experimental Procedures, Figures S1–S4, and Table S1–S3

Document S2. Article plus Supplemental Information
