# Supplementary material for: Multiparameter Analysis of Human Bone Marrow Stromal Cells Identifies Distinct Immunomodulatory and Differentiation-Competent Subtypes
Source: Stem Cell Reports. 2015 Jun 9;4(6):1004–15. doi: 10.1016/j.stemcr.2015.05.005 (PMC4471830; doi:10.1016/j.stemcr.2015.05.005)
Supplement: Document S1. Supplemental Experimental Procedures, Figures S1–S4, and Table S1–S3 [file mmc1.pdf]

Stem Cell Reports

Supplemental Information

Multiparameter Analysis of Human Bone Marrow Stromal Cells Identifies Distinct Immunomodulatory and Differentiation-Competent Subtypes

Sally James, James Fox, Farinaz Afsari, Jennifer Lee, Sally Clough, Charlotte Knight, James Ashmore, Peter Ashton, Olivier Preham, Martin Hoogduijn, Raquel De Almeida Rocha Ponzoni, Y. Hancock, Mark Coles, and Paul Genever

SUPPLEMENTAL FIGURES

Figure S1

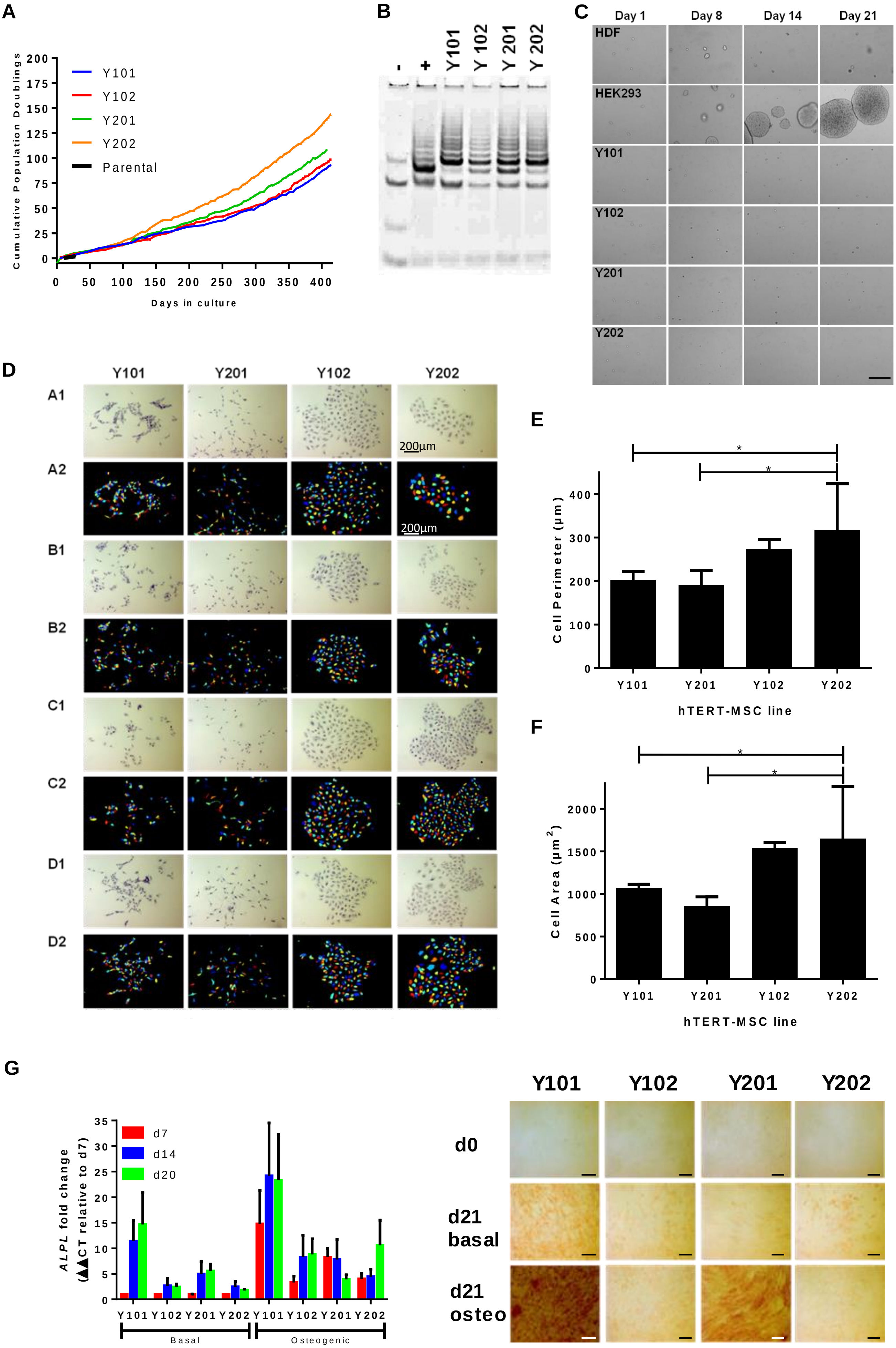

Figure S2

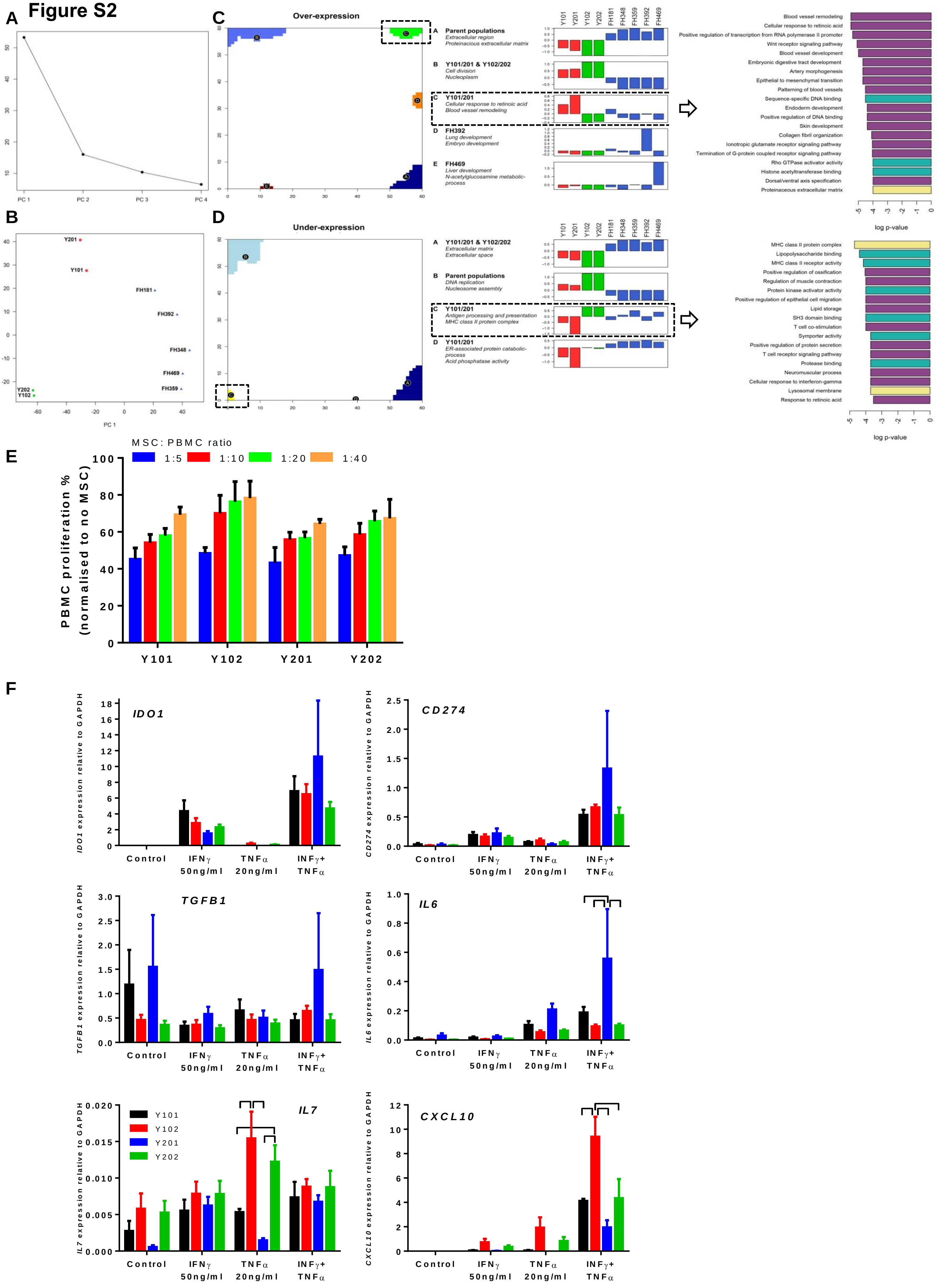

A
% Variance
PC 1
PC 2
PC 3
PC 4
B
Y201
Y101
FH181
FH392
FH348
FH469
FH359
Y202
Y102
PC 1
PC 2
C
Over-expression
A Parent populations
Extracellular region
Proteinacious extracellular matrix
B Y101/201 & Y102/202
Cell division
Nucleoplasm
C Y101/201
Cellular response to retinoic acid
Blood vessel remodeling
D FH392
Lung development
Embryo development
E FH469
Liver development
N-acetylglucosamine metabolic-process
Y101
Y201
Y102
Y202
FH181
FH348
FH359
FH392
FH469
Blood vessel remodeling
Cellular response to retinoic acid
Positive regulation of transcription from RNA polymerase II promoter
Wnt receptor signaling pathway
Blood vessel development
Embryonic digestive tract development
Artery morphogenesis
Epithelial to mesenchymal transition
Patterning of blood vessels
Sequence-specific DNA binding
Endoderm development
Positive regulation of DNA binding
Skin development
Collagen fibril organization
Ionotropic glutamate receptor signaling pathway
Termination of G-protein coupled receptor signaling pathway
Rho GTPase activator activity
Histone acetyltransferase binding
Dorsal/ventral axis specification
Proteinaceous extracellular matrix
log p-value
D
Under-expression
A Y101/201 & Y102/202
Extracellular matrix
Extracellular space
B Parent populations
DNA replication
Nucleosome assembly
C Y101/201
Antigen processing and presentation
MHC class II protein complex
D Y101/201
ER-associated protein catabolic-process
Acid phosphatase activity
MHC class II protein complex
Lipopolysaccharide binding
MHC class II receptor activity
Positive regulation of ossification
Regulation of muscle contraction
Protein kinase activator activity
Positive regulation of epithelial cell migration
Lipid storage
SH3 domain binding
T cell co-stimulation
Symporter activity
Positive regulation of protein secretion
T cell receptor signaling pathway
Protease binding
Neuromuscular process
Cellular response to interferon-gamma
Lysosomal membrane
Response to retinoic acid
log p-value
E
MSC: PBMC ratio
1:5
1:10
1:20
1:40
PBMC proliferation %
(normalised to no MSC)
Y101
Y102
Y201
Y202
F
IDO1
IDO1 expression relative to GAPDH
CD274
CD274 expression relative to GAPDH
TGFB1
TGFB1 expression relative to GAPDH
IL6
IL6 expression relative to GAPDH
IL7
IL7 expression relative to GAPDH
CXCL10
CXCL10 expression relative to GAPDH
Control
IFNγ 50ng/ml
TNFα 20ng/ml
INFγ+ TNFα
Y101
Y102
Y201
Y202

Figure S3

A

**Cell/Tissue**	**Femur**	**Tibia**	**Sternum**	**Calvaria**
Central marrow	3.9	3.9	5.2	3.4
Endosteal region	5.4	6.0	8.5	2.2
Articular chondrocyte	60.0	54.0	11.1	nd
Hypertrophic chondrocyte	13.8	3.6	31.0	6.9
Osteocyte	9.5	22.9	nd	7.6

B

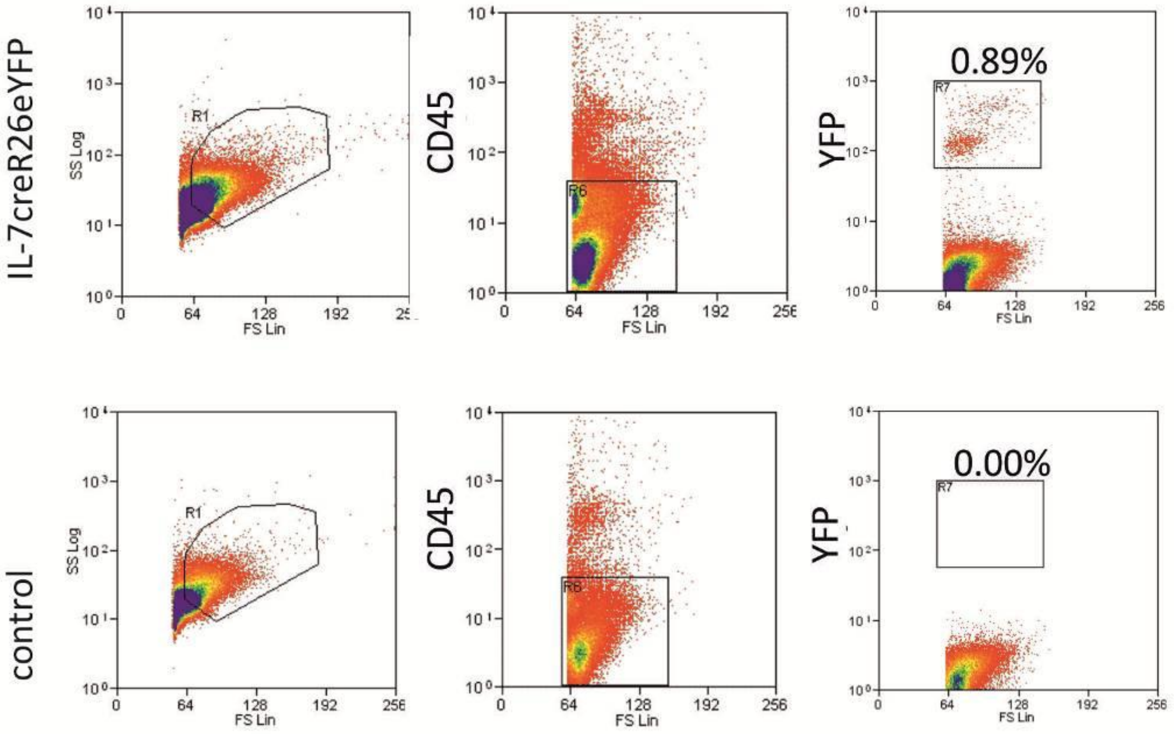

C

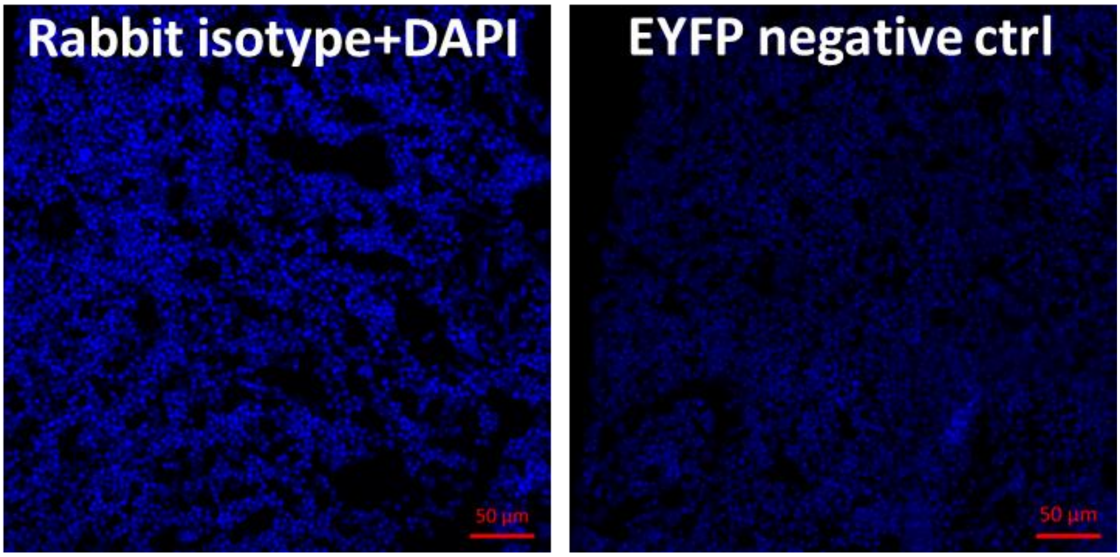

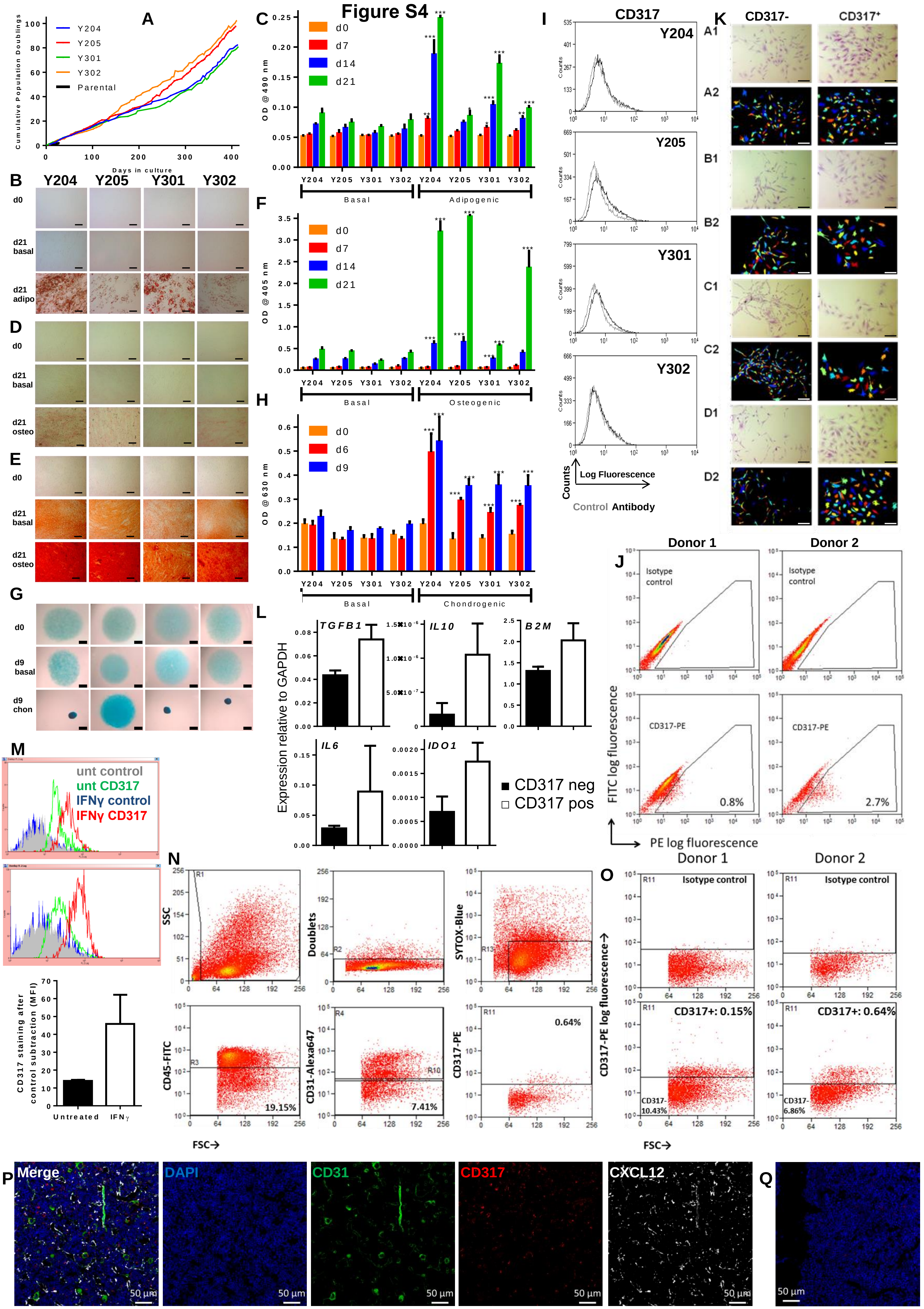

Figure S4
A
B
C
D
E
F
G
H
I
J
K
L
M
N
O
P
Q
Cumulative Population Doublings
Days in culture
Y204
Y205
Y301
Y302
Parental
d0
d21 basal
d21 adipo
d21 osteo
d9 basal
d9 chon
d7
d14
d21
d6
d9
OD @ 490 nm
OD @ 405 nm
OD @ 630 nm
Basal
Adipogenic
Osteogenic
Chondrogenic
CD317
Counts
Log Fluorescence
Control Antibody
CD317-
CD317+
A1
A2
B1
B2
C1
C2
D1
D2
Donor 1
Donor 2
Isotype control
CD317-PE
0.8%
2.7%
FITC log fluorescence
PE log fluorescence
Expression relative to GAPDH
TGFB1
IL10
B2M
IL6
IDO1
CD317 neg
CD317 pos
unt control
unt CD317
IFNγ control
IFNγ CD317
CD317 staining after control subtraction (MFI)
Untreated
IFNγ
SSC
Doublets
SYTOX-Blue
CD45-FITC
CD31-Alexa647
CD317-PE
FSC→
19.15%
7.41%
0.64%
CD317-PE log fluorescence→
CD317+: 0.15%
CD317+: 0.64%
CD317- 10.43%
CD317- 6.86%
Merge
DAPI
CD31
CD317
CXCL12
50 µm

SUPPLEMENTAL FIGURE LEGENDS

Figure S1. Generation and analysis of hTERT-BMSC clones, Y101, Y102, Y201 and Y202, Related to Figure 1.

(A) Cumulative population doublings of hTERT-BMSC clonal lines with the original parental primary BMSCs.

(B) Telomerase activity measured in the four hTERT-BMSC clones against positive (+) and negative (-) controls. Banding indicates PCR amplification of telomeric repeats.

(C) Anchorage-independent growth assays in soft agarose assays using the hTERT-BMSC clones Y101, Y102, Y201 and Y202, alongside human diploid fibroblast (HDF) and HEK-293 cells as negative/positive controls respectively. Representative images of colonies taken at same magnification are shown after 1, 8, 14 and 21 days of culture, scale bar = 250μm.

(D) Microscopic examination of colonies formed by Y101, Y102, Y201 and Y202 clones. Toluidine blue stain (A1-D1) and CellProfiler analysis (A2-D2), four representative images shown. Images are all taken at same scale, scale bar = 200μm.

(E-F) Cell perimeter (E) and area (F) measurements of hTERT-BMSC clones using CellProfiler software. The mean ± SEM of four independent experiments examining on average 124 cells per experiment is shown. Data were analysed for statistically significant differences in perimeter or area using 1way ANOVA and Newman-Keuls multiple comparison tests between the hTERT –BMSC lines; *=$p<0.05$.

(G) Histological ALP staining and real time qPCR gene expression analysis for *ALPL* of the hTERT-BMSCs cultured in osteogenic differentiation media. Mean gene expression levels ± SEM are shown (average of three experiments performed in triplicate). Scale bar = 200μm.

Figure S2. Bioinformatic and functional analysis of hTERT-BMSC clones, Related to Figure 2.

(A-B) Principal component analysis of Y101, Y102, Y201 and Y202 clones against the parent BMSC population (FH181) and four other primary BMSC cultures (FH392, FH348, FH469 and FH359).

(C-D) Analysis of over- (C) and under- (D) expressed metagenes in Y101, Y102, Y201 and Y202 clones, the parent BMSC population (FH181) and four additional primary BMSC cultures (FH392, FH348, FH469 and FH359).

(E) Peripheral blood mononuclear cell (PBMC) proliferation assay in the presence of hTERT-BMSCs at the stated ratios. Data represents average inhibition of PBMC proliferation ± SEM from five experiments performed in triplicate. 2way ANOVA with Sidak's multiple comparisons between the 4 cell lines under the different conditions were performed, revealing no significant difference at any cell:cell ratio.

(F) Expression of immunomodulatory factors (*IDO1, CD274, TGFB1, IL6, IL7 and CXCL10*) by hTERT-BMSC clones following exposure to TNFα and/or INFγ. Mean expression levels from three experiments performed in triplicate ± SEM are shown. Where there was significant difference in gene expression between control and treated cells, significant differences between the cell lines was assessed by 2way ANOVA and differences are indicated as brackets across the data points at at least $p<0.05$.

Figure S3. Identification of EYFP-positive cells in bone/marrow of IL-7cre Rosa26-EYFP lineage-tracing mice, Related to Figure 3.

(A) Distribution of EYFP-positive cells in bone. Slides were scored for the number of EYFP-positive cells per field as a percentage of total nucleated cells as stained by DAPI, nd = not determined.

(B) Bone marrow cells were isolated from the femora and tibiae of IL-7cre Rosa26-EYFP mice and analysed by flow cytometry. The R1 gate represents live cells separated by FSC/SSC, R6 gate discriminates between CD45-negative, non-hematopoietic cells and CD45-positive cells, R7 gate identifies CD45-negative, EYFP-positive cells. Representative flow cytometric data shown.

(C) Representative isotype control IL-7cre Rosa26-EYFP bone marrow section and bone marrow section showing EYFP negative control stained with the anti-GFP, alexa fluor 488 conjugated antibody. DAPI nuclear stain (blue).

Figure S4. Validation of CD317 expression in BMSC subpopulations, Related to Figure 4.

(A) Cumulative population doublings of four additional hTERT-BMSC clonal lines (Y204, Y205, Y301, Y302) with the original parental primary BMSCs (experiment performed alongside data shown in Figure S1A).

(B-H) Assessment of the OAC potential of Y204, Y205, Y301 and Y302 BMSC clones; representative images (B, D, E, G) and quantification (C, F, H) of cells differentiated with adipogenic (B, C), osteogenic (D, E, F) and chondrogenic (G, H) supplements. Scale bar = 200µm in B, D & E whilst in G, scale bar = 2mm. Data represents average quantified values ± SD for 3 independent experiments performed using 6 replicates. 2way ANOVA with Sidak's multiple comparisons were used to assess statistically significant differences in the three measures of differentiation between basal and induced conditions at each time point for each cell line. Statistical significance is denoted by *=p<0.05; **=p<0.01; ***=p<0.001.

(I) Representative flow cytometric analysis of CD317 expression in Y204, Y205, Y301 and Y302 BMSC clones.

(J) Identification of rare CD317^{+} cells in heterogeneous primary BMSC cultures by flow cytometry, two example donors shown.

(K) Microscopic examination of colonies formed by CD317^{-} and CD317^{+} cells sorted from primary BMSCs. Crystal violet stain (A1-D1) and CellProfiler analysis (A2-D2), four representative images shown. Scale bar represents 200µm

(L) Expression of immunomodulatory factors by CD317^{-} and CD317^{+} cells sorted from primary BMSCs by qPCR. Mean ± SEM of gene expression in sorted cells from two donors is shown.

(M) CD317 staining on untreated (unt) primary BMSCs or following treatment with 50ng/ml IFN-γ for 24hrs. Flow cytometry histograms for the two donors (left and middle) are shown and CD317 mean fluorescence intensity (MFI) is quantified (right) following subtraction of the control staining in the adjacent histograms. Mean MFI ± SD of two independent experiments are shown in the histogram.

(N) Gating strategy for flow cytometric sorting of human bone-marrow mononuclear cells (BM-MNCs). Debris, doublets, dead cells (sytox blue positive), CD45-positive and CD31-positive cells were removed. Percentages shown are the cell populations of the live (sytox blue negative) cells. Data

shown are representative of two independent experiments using two separate preparations of primary human BM-MNCs.

(O) Representative flow cytometric analysis of CD317 expression in BM-MNCs: two donors are shown with the percentage of total live cells negative for CD45 and CD31 but expressing CD317 displayed.

(P) Representative staining of mouse bone marrow sections. Scale bar represents 50µm. Blue = nuclear DAPI, green = CD31, red = CD317 and white = *Cxcl12*-DsRed. Single channel and merged overlay images are shown.

(Q) Representative antibody isotype control for the donkey anti-rabbit antibody used as a secondary antibody to reveal CD317 staining on mouse bone marrow sections in Fig 4F/G. Scale bar represents 50µm. Blue = DAPI; Red = isotype control plus donkey anti-rabbit secondary antibody.

SUPPLEMENTAL TABLES

Target	Conjugate	Supplier	Catalogue number
hCD45	FITC	eBioscience	11-9459
hCD166	PE	BD Pharmingen	559263
hCD44	FITC	BD Pharmingen	555478
hCD90	None	eBioscience	14-0909
hCD105	APC	eBioscience	17-1057
hCD29	Purified (none)	BD Pharmingen	556048
hCD73	Purified (none)	BD Pharmingen	550256
hCD34	FITC	Miltenyi Biotec	130-081-001
hCD317	PE	BioLegend or eBioscience	348406 or 12-3179
hCD271	FITC	Miltenyi Biotec	130-091-917
hCD51/CD61	None	BD Pharmingen	555504
hPDGFRα	None	Santa Cruz Biotechnology	sc-21789
hCD146/MCAM	None	Abcam	ab24577
hICAM	None	BD Pharmingen	555510
hCD274	APC	eBioscience	17-5983
hCD295	PE	R&D Systems	FAB867P
hCD31	Alexa Fluor 647	BD Pharmingen	558094
hIL-7	None	R&D Systems	MAB207
mCD317	None	Novus Biologicals	NBP2-27154SS
mCD295	Biotin	R&D Systems	BAF497
mPerilipin A/B	None	Bioss	bs-6765R
mIgG2b, isotype control	PE	Biolegend	400313
mIgG1, isotype control	None	eBioscience	14-4714
Goat anti-mouse-IgG	Alexa Fluor 488	Invitrogen	A11001
Goat anti-rabbit IgG	Alexa Fluor 488	Invitrogen	A11008
Donkey anti-rabbit-IgG	Alexa Fluor 647	Invitrogen	A31573
Anti-GFP tag	Alexa Fluor 488	Invitrogen	A21311

Table S1. Antibodies used for flow cytometry. Key: h=human; m=mouse.

**Peak assignment cm^{-1}**	**Peak Identification***
717.1	Lipids
779.3	DNA/RNA nucleic acids
849.8	Proteins/amino acids
966.0	DNA/RNA nucleic acids
999.6	Phenylalanine
1088.6	PO^{-}_{2} stretch DNA
1203.7	Amide III and other amino acids/nucleic acids
1239.4	Amide III
1334.8	Lipids/nucleic acids/proteins
1447.3	Proteins and lipids
1657.8	Lipids

Table S2. Mean Raman peak assignments corresponding to the averaged spectra per cell line (Figure 4H, I); maximum uncertainties in the peak measurements are ± 0.8 cm^{-1}, corresponding to the spectral resolution of the instrument.

**Gene Target**	**Primer sequence**
Peroxisome proliferator activated receptor (*PPAR*)-γ forward	GGCTCCATGACAAGGGAGTTTC
PPARγ reverse	AACTCAAACTTGGGCTCCATAAAG
Lipoprotein lipase (*LPL*) forward	GAGGTACTTTTCAGCCAGGATGTAAC
LPL reverse	AGCTGGTCCACATCTCCAAGTC
Ribosomal protein S (*RPS*)*27a* forward	TGGATGAGAATGGCAAAATTAGTC
RPS27a reverse	CACCCCAGCACCACATTCA
Alkaline Phosphatase (*ALPL*) forward	GGGAACGAGGTCACCTCCAT
ALPL reverse	TGGTCACAATGCCCACAGAT
RUNX2 forward	GGTTAATCTCCGCAGGTCAC
RUNX2 reverse	GTCACTGTGCTGAAGAGGCT
SOX9 forward	TTCCGCGACGTGGACAT
SOX9 reverse	TCAAACTCGTTGACATCGAAGGT

Table S3. qPCR primer sequences

SUPPLEMENTAL EXPERIMENTAL PROCEDURES

Primary BMSC isolation and culture

Primary human BMSCs were isolated from femoral heads obtained with informed consent during routine hip replacement, as previously described (Dyson et al., 2007; Etheridge et al., 2004), or as explant cultures from human tibial plateaus after routine knee replacement surgery. Cells were cultured in DMEM containing 15% FBS, 100U/mL penicillin and 100µg/mL streptomycin, which was changed every 3–4 days; cells were passaged at 70-80% confluency. Cells were expanded and used between passages (p)1 and p5.

hTERT BMSC production

The hTERT lentiviral vector was produced using the ViraPower Lentiviral Gateway Expression Kit (Invitrogen) according to manufacturer's guidelines, and as previously described (Saleh et al., 2012) before transduction into primary human BMSCs from a single donor. After clonal selection, the human telomerase gene was subcloned from pCI-neo-hEST2 (Addgene, courtesy of Robert Weinberg) into the pENTR 1A backbone provided using restriction enzyme digestion and ligation. After appropriate screening, an LR recombination reaction was performed, successfully incorporating the hTERT gene into pLent6/V5-DEST lentiviral vector.

pLenti6/V5-DEST-hTERT lentiviral stocks were produced using 293FT cells according to manufacturer's instructions. 24hrs after lipofectamine transfection of pLenti6/V5-DEST-hTERT and viral packaging mix, culture media was replaced with 5ml fresh culture medium to concentrate viral particles. Lentivirus-containing medium was harvested 48hrs post-transfection, and lentiviral stocks were titered using the Lenti-X qRTPCR Titration Kit (Clontech).

BMSCs were transduced with pLenti6/V5-DEST-hTERT in the presence of 6µg/ml polybrene. After 16hrs the lentivirus-containing medium was removed and replaced with fresh BMSC media. After a further 24hrs, the cells were passaged and 2µg/ml blasticidin was added to the culture media. Media was replaced every 3-4 days with fresh blasticidin-containing medium for 12 days, when no living mock-transfected cells remained. To select single cell lines the transduced BMSCs were trypsinized

and plated in 10cm plates at 10 cells/cm^2. Cells were grown in BMSC medium containing 20% HyClone serum for plating out the cells, then replaced with fresh BMSC medium containing 15% HyClone serum every 3/4 days for 14 days (until discrete single cell colonies were visible). Single cell colonies were isolated using cloning cylinders, silicone grease, and trypsin-EDTA and then transferred to wells of 24-well plates. Once the cells reached 70% confluence they were passaged into 6-well plates, then 25cm^2 flasks, and finally 75cm^2 flasks for continued expansion. Cells were counted at each passage to determine population doubling time and maintained for continued culture in DMEM containing 10% FBS and antibiotics. Telomerase activity in hTERT-BMSCs was assessed using the TRAPeze® gel-based telomerase detection kit (Millipore) according to manufacturer's guidelines. Analyses using CellProfiler software were performed according to the designer instructions (www.cellprofiler.org).

I*n vitro* hTERT BMSC differentiation

In vitro induction of BMSC differentiation was performed as previously outlined (Etheridge et al., 2004; Hoogduijn et al., 2006; Saleh et al., 2011). Osteogenic differentiation was induced for up to 21 days with the addition of 5mM β-glycerophosphate, 50□g/mL L-ascorbic acid phosphate and 10nM dexamethasone to growth media. Osteogenesis was assessed by both histological staining with alizarin red and quantitative elution of the stain alongside quantitative assays for alkaline phosphatase (ALP) activity (Cook et al., 2014; Hoogduijn et al., 2006) and gene expression analyses for Runx2 and ALP. Adipogenic differentiation was induced with 0.5mM isobutyl-methylxanthine, 1µg/ml insulin, 100µM indomethacin and 1µM dexamethasone for up to 21 days. Lipid accumulation was examined and quantified by Oil Red O staining (Cook et al., 2014) and gene expression analyses for *PPARγ* and *LPL* were performed. Micromass pellet cultures were utilized for chondrogenesis assays. Briefly, cells were trypsinized and resuspended to 2x10^7 cells/ml in serum-free media; 20µl cell suspension was placed in the middle of a culture well and allowed to adhere at 37°C for 3h. Wells were flooded with serum-free media containing 50µg/ml L-ascorbic acid phosphate, 100nM dexamethasone, 40µg/ml L-proline, 1% ITS$^+$, with or without TGF-β; medium was changed twice

weekly. Chondrogenic differentiation was assessed using 9 day micromass cultures and potential was indicated through an early cell condensation phenotype (Johnson et al., 2012). After 1, 6 or 9 days of culture, cells were fixed in 100% methanol (-20°C, 30 mins), then incubated overnight with 0.5% alcian blue 8GS in 1M HCl (pH 0.2 to selectively stain highly sulphated glycosaminoglycans (GAGs) associated with cartilage tissue). Cells were washed extensively with distilled water before stereomicroscope visualisation. Specific GAG-associated stain was eluted from pellets by 6M guanidine incubation for 3 hours with rocking; supernatant absorbance was measured at 630nm. Total GAG content (pellet associated and secreted) for each cell line following chondrogenic induction was measured by the Blyscan glycosaminoglycan assay (Biocolor Ltd, UK) according to the manufacturer's instructions. Gene expression analyses by qPCR for the early chondrogenic marker Sox9 were also performed.

Flow cytometry and cell sorting

Cell surface marker profiles were assessed on a CyAn flow cytometer and analyzed using Summit software (v4.3; Beckman Coulter). Cells were detached from culture plastic by incubation with PBS containing 0.2% BSA and 5mM EDTA, and stained with either directly conjugated primary antibodies, or successive incubations with optimised concentrations of primary antibody and an appropriate fluorescent-conjugated secondary antibody. All antibodies are listed in Table S1. For cell sorting, BMSCs were processed and stained as above then sorted on a MoFlo Astrios high-speed, sterile cell sorter (Beckman Coulter). Cryopreserved, primary human bone marrow mononuclear cells (BM-MNCs) were purchased from Lonza (Belgium); both healthy donors were male Caucasians, donor one was 21 years of age, donor two was 22. Cells were thawed according to the Poietics protocol, rested for an hour before staining for 40mins with optimised concentrations of CD45, CD31 and CD317 antibodies or equivalent concentrations of isotype controls before sorting. Cells were also extracted from femora and tibiae bone marrow of IL-7cre Rosa26-EYFP mice and incubated with ACK buffer to lyse any red blood cells prior to flow cytometric analysis. CD317 staining was also

assessed on primary cells after 50ng/ml recombinant human IFN-γ (PeproTech EC Ltd, UK) treatment for 24 hours.

Anchorage independent growth assay

Anchorage independent growth assays as an assessment of tumorigenic potential were performed on hTERT BMSC-lines alongside HEK-293 cells as positive controls and human dermal fibroblasts as a negative control. Cells were seeded into media containing 0.45% agarose in plates coated with 0.75% agarose and cultured for up to 21 days before imaging by brightfield microscopy.

RNA isolation and gene expression analysis

Total RNA was isolated from 80-90% confluent cultures using Macherey-Nagel Nucleospin RNA II kit. RNA quality was determined with the Agilent 2100 Bioanalyzer nano chip; high quality RNA samples were labelled using Agilent One Color Quick Amp labelling. Samples were processed for genome-wide gene expression analysis using the Agilent SurePrint G3 Human Gene Expression 8x60K v2 Microarray kit; triplicate arrays were performed on each cell line. All quality control checks met required standards. Data were analyzed using GeneSpring version 12.1 software (Agilent Technologies), using initial one-way ANOVA with controlling for multiple testing. This allowed for p-value adjustment lowering false discovery rate whilst maintaining sensitivity. Genes with a minimum two-fold difference in expression level and an adjusted p-value of <0.05 were considered differentially expressed. Subsequent pathway analysis was performed on lists of differentially expressed genes within the GeneSpring software. Additional analyses were performed using the statistical programming language R and the Bioconductor package limma.

Quantitative real-time PCR (qPCR) was performed using two protocols; the first used 500ng cDNA with the StepOnePlus™ Real-Time PCR System, TaqMan Universal PCR Master Mix and the assay-on-demand primer/probes for *IDO1* (Hs00158027.m1), *IL6* (Hs00174131.m1), IL7 (Hs00174202.m1), *IL10* (Hs00174086.m1), *TGFB1* (Hs00171257.m1), *CD274* (Hs00204257.m1), *CXCL10* (Hs00171042.m1) and *B2M* (Hs00187842.m1) (all Applied Biosciences, Foster City, CA,

USA). Glyceraldehyde 3-phosphate dehydrogenase (GAPDH) mRNA served as endogenous control for normalization (Hs99999905.m1; Applied Biosciences). Intra- and inter-assay variations were determined by negative controls and positive reference samples. Changes in target gene expression relative to GAPDH were quantified using the comparative CT method. The second method used the SYBR green system and optimised amounts of cDNA reverse transcribed using superscript II (Invitrogen, UK) from 1µg of RNA isolated from primary BMSCs differentiated along the osteogenic, adipogenic or chondrogenic lineages using Trizol/chloroform extraction (Life Technologies, UK). cDNA was amplified using Fast SYBR Green Master Mix (Life Technologies) and optimised primers (Table S3) for analysis of adipogenic genes (peroxisome proliferator-activated receptor gamma and lipoprotein lipase), osteogenic genes (alkaline phosphatase and *RUNX2*) or a chondrogenic gene (*SOX9*) using the StepOne real-time PCR system. Gene expression levels were quantified using the comparative CT (ΔΔCt) method and StepOne software v2.3 relative to the expression of the housekeeping gene RPS27a and normalized to d7 basal levels.

Agilent microarray data processing

Preprocessing of the microarray data was carried out using the statistical programming language R and the Bioconductor package limma. Firstly, background correction with optimal offset value was applied to the arrays to minimize signal related to non-specific fluorophore binding. The optimal offset value was evaluated by identifying which value stabilized the variances of probeset intensities the most. Quantile normalization was then applied to the arrays, which resulted in each array having the same empirical distribution of probeset intensity values, removing systematic variation from the data and scaling it so comparisons could be made between them. The values were then transformed by $\log_2$ to control for variability in both highly and poorly expressed genes. Uninformative data such as control probesets and genes with low variance were also removed to decrease the false discovery rate (FDR). Lastly, the average expression levels from each of the replicates were collected into a global expression dataset.

Microarray data can be found on ArrayExpress: E-MTAB-3511 (release date: 03-August-2015).

Differential gene expression analysis

Differentially expressed genes were defined as those which exhibited a log fold-change of greater than two. The statistical significance of this differential expression was evaluated using a moderated t-test, known as Empirical Bayes. Instead of calculating an estimate of the variance for each gene, this moderated version calculates a global variance from the variances for all genes in the dataset. This is combined with an average of the variance for each gene producing a statistical test value that has a t-distribution. The Bioconductor package limma was used to calculate this log fold-change and significance value by applying a linear model to the probe set intensities for each gene using a least squares method. Limma also employed the Benjamini and Hochberg algorithm to control for the effects of multiple testing. This procedure allowed for the adjustment of the p-values and the lowering of the false discovery rate whilst keeping the sensitivity for true positive results. Genes with a two-fold difference in expression levels and with an adjusted p-value of ≤ 0.05 between any pair of cell lines in the dataset were considered as significantly differentially expressed. The differentially expressed genes limma outputs were then collected into a differential expression dataset for use in subsequent analyses.

Sample and gene clustering algorithms

A preliminary insight into the variation of gene expression profiles between cell lines was achieved by performing principal component analysis (PCA) on the differential expression dataset using the R CRAN package stats. This approach reduces the dimensionality of the dataset into two orthogonal variables, visualised as a two-dimensional scatterplot of the cell lines. Cell lines that cluster close together in two-dimensional space are considered to have more similar expression profiles than those further away.

SOM-mapping of global expression profiles

Self-organizing map (SOM) machine learning was applied to the global expression dataset using the R CRAN package oposSOM (Wirth et al., 2011). This unsupervised learning method uses neural networks to reduce high dimensional data onto a two-dimensional mosaic. Each tile on the mosaic

represents a metagene consisting of mini-clusters of genes with similar expression profiles. Genes are assigned to clusters using the minimum Euclidian distance and metagenes with similar expression profiles are positioned next to each other. Because the same metagene is assigned to the same tile in SOMs across all samples, direct visual comparisons of global expression can be made and areas of notable over- or under- expression are readily observed. After running the analysis, oposSOM outputs a SOM for each cell line, a combined SOM of over- and under- expressed metagene hotspots, including a list of genes which contribute to those metagenes and their relative expression in each cell line.

Gene set over-representation analysis

Gene set over-representation analysis was used to help define the functional nature of the respective spots. For each of the spots the degree of over-representation of 1454 pre-defined gene sets was measured using the hypergeometrical distribution (Wirth et al., 2011). This assigns the probability that members of a certain gene set are present in a list of genes compared against the random chance of their appearance independent of expression values. Gene sets contain lists of genes which have been shown to act in the same pathway or appear to be coexpressed. An over-representation p-value is then used to evaluate the significance of over-representation of each gene set to each metagene. The hypergeometrical distribution then ranks the list of gene sets by decreasing significance of over-representation for the spots. In addition, gene ontologies were used to describe the attributes of each gene product in these hotspots. Gene ontologies provide a comprehensive and unified way of annotating gene products with i) the biological process to which they contribute ii) their functional biochemical activity iii) the cellular component where they are active (Ashburner et al., 2000). Using a hypergeometrical distribution, the statistical over-representation of these terms with regards to the global expressed set was also calculated. Again, oposSOM was used to carry out this analysis, and given the row names of the expression dataset are labelled using ENSEMBL gene IDs is automatically performed when the function is called.

Immunofluorescent staining for IL-7

To identify IL-7 immunopositivity in hTERT-BMSCs, $2x10^4$ cells were seeded onto coverslips, allowed to adhere overnight then fixed in 4% PFA for 5mins at 21°C before permeabilization with 0.1% Triton X-100 in PBS and blocking of endogenous peroxidase activity with 1% H_2O_2. All future steps were performed at room temperature in a humidified chamber in the presence of 1% blocking reagent supplied with the tyramide signal amplification kit. Extensive washes were performed between antibodies; all reagents were from Invitrogen unless otherwise stated. Samples were initially blocked for 30mins in PBS containing 1% blocking reagent before incubations for 60mins with optimised concentrations of either mouse anti-human IL-7 primary antibody (R&D clone 7417/catalogue number MAB207) or an IgG1 isotype control. Coverslips were incubated with 1:200 biotinylated anti-mouse secondary antibody for 30mins before incubation with 1:100 diluted horseradish peroxidase for 45mins. Alexa Fluor-647 conjugated tyramide was 1:100 diluted in amplification buffer and incubated with the samples for 5mins. Finally, samples were extensively washed before mounting in Prolong gold containing DAPI and imaged using an LSM710 invert confocal microscope (Carl Zeiss).

In vivo IL-7 lineage tracing

IL-7cre Rosa26-EYFP mice were bred under pathogen free conditions at the University of York (Repass et al., 2009). All work was in accordance with ethical approvals from the University of York and Home Office License 60/4169. For lineage tracing experiments, tissues from IL-7cre Rosa26-EYFP mice were fixed in 4% PFA overnight and placed at room temperature in 30% sucrose solution for 15mins followed by 15% sucrose overnight at 4°C. Tissues were then coated with 10% polyvinyl alcohol (PVA) and frozen in cooled isopropanol on dry ice. They were then fully mounted in 10% PVA and sectioned (at10μm) using a Bright's cryostat, slide-mounted and observed by fluorescence microscopy.

Immunohistochemistry

Bone samples from the femurs of 11- or 12-week old female hybrid C57BL/Ka x C57BL/6 mice (derived from in-house crossing of *Cxcl12*$^{tm2.1Sjm}$/J (The Jackson Laboratory) and wild-type C57BL/6 mice) or IL-7cre Rosa26-EYFP and non-EYFP expressing mice were used. These mice express DsRedE2 under control of the *Cxcl12* promoter (Ding and Morrison, 2013) or IL-7 EYFP, respectively. Samples were fixed overnight at 4°C in 4% paraformaldehyde/75mM lysine/10mM periodate fixative. Bones were decalcified for 3 days in 10% EDTA, 0.1M Tris, pH7.2 at 4°C with agitation before overnight incubation in 30% sucrose in PBS at 4°C and embedding in optimal cutting temperature embedding media (Tissue-Tek). 5µm sections were cut, applied to slides and blocked for 1hr at room temperature in staining buffer (PBS + 0.05% BSA + 5% species specific serum) before staining with optimised concentrations of primary antibodies in staining buffer at 4°C overnight. Three five-minute washes (PBS + 0.05% BSA) were performed before slides were incubated with optimised concentrations of relevant secondary antibody for 1hr at RT. Where indicated, anti-GFP tag antibody was applied for 1hr. Three further washes, DAPI counterstaining and prolong gold mounting (Invitrogen) were performed before imaging using an LSM710, LSM780 or LSM 510 confocal microscopes (Carl Zeiss) and image analysis with Zen 2012 (blue edition) software (Carl Zeiss).

ELISA for IL-7 secretion

Cells were seeded into 6 well plates at $2x10^4$ cells/cm^2 and left to adhere overnight. Culture media was replaced with 2ml fresh media; 24h later the quantity of IL-7 in the supernatant was measured using the Quantikine HS Human IL-7 Immunoassay (R&D systems) according to the manufacturer's guidelines, and compared against medium alone.

Peripheral blood mononuclear cell (PBMC) proliferation assay

PBMCs were incubated with the hTERT-BMSCs at ratios of 1:5, 1:10, 1:20 and 1:40. PBMC proliferation was quantified by detection of ^{3}H-thymidine incorporation into the PBMCs after three days of stimulation with anti-CD3 anti-CD28 antibodies.

Raman Methods

The hTERT BMSCs and CD317+ cells were seeded at $5x10^4$ cells per CaF_2 microscope slide; slides were incubated in growth media with 0.1% amphotericin B for 24h before replacement with DMEM containing 0.5% FBS and another 24h incubation for G_0 synchronisation. Before Raman analysis, slides were PBS rinsed twice and air-dried. Raman spectra were collected using a HORIBA XploRA micro-Raman instrument with 532nm laser wavelength and x100 objective (NA = 0.9) in confocal setting. Spectra were acquired ambiently using 45s laser exposure resulting in 3.5mW power at the sample. Each measurement was obtained using a 2400 lines/mm diffraction grating, with the results averaged over 2 spectral acquisitions over the 'finger-print' range ($600cm^{-1}$ to $1800cm^{-1}$). Raman spectra were collected from hTERT BMSC lines using 20 randomly selected cells, with five randomly selected points taken in the nucleus of each cell. The resulting 100 spectra per BMSC line were baseline-corrected and averaged so that a representative spectrum of each BMSC population was obtained. For the CD317+ cell line, a Raman spatial map consisting of 172 spectra across the nucleus was acquired. These spectra were baseline-corrected and averaged to obtain a representative spectrum of the individual CD317+ cell under investigation. To prevent laser-induced cell damage, Raman spectra were monitored during real-time data acquisition, with optical inspection also being performed after each Raman measurement. All spectra were collected using the LabSpec 5 software (HORIBA). Baseline-correction was performed to each spectrum using an interval linear fitting method obtained in the '*Raman tool set'* software (Candeloro et al., 2013). Further analysis on the averaged spectra, including individual peak baselines and Gaussian peak fitting, was conducted using IGOR Pro 6.32. Raman markers were determined from the set of two-peak intensity ratios derived from key peaks in the averaged cell spectra.

Data Analysis

Data were analysed in GraphPad Prism v6.05; statistical analyses using 2way ANOVA followed by Sidak's multiple comparisons, paired t-test or 1way ANOVA with relevant multiple comparison test were used, where indicated.

SUPPLEMENTAL REFERENCES

Ashburner, M., Ball, C.A., Blake, J.A., Botstein, D., Butler, H., Cherry, J.M., Davis, A.P., Dolinski, K., Dwight, S.S., Eppig, J.T., *et al.* (2000). Gene Ontology: Tool for the Unification of Biology. The Gene Ontology Consortium. Nature genetics. *25*, 25-29.

Candeloro, P., Grande, E., Raimondo, R., Di Mascolo, D., Gentile, F., Coluccio, M.L., Perozziello, G., Malara, N., Francardi, M., and Di Fabrizio, E. (2013). Raman Database of Amino Acids Solutions: A Critical Study of Extended Multiplicative Signal Correction. The Analyst. *138*, 7331-7340.

Cook, D.A., Fellgett, S.W., Pownall, M.E., O'Shea, P.J., and Genever, P.G. (2014). Wnt-Dependent Osteogenic Commitment of Bone Marrow Stromal Cells Using a Novel Gsk3beta Inhibitor. Stem cell research. *12*, 415-427.

Ding, L., and Morrison, S.J. (2013). Haematopoietic Stem Cells and Early Lymphoid Progenitors Occupy Distinct Bone Marrow Niches. Nature. *495*, 231-235.

Dyson, J.A., Genever, P.G., Dalgarno, K.W., and Wood, D.J. (2007). Development of Custom-Built Bone Scaffolds Using Mesenchymal Stem Cells and Apatite-Wollastonite Glass-Ceramics. Tissue engineering. *13*, 2891-2901.

Etheridge, S.L., Spencer, G.J., Heath, D.J., and Genever, P.G. (2004). Expression Profiling and Functional Analysis of Wnt Signaling Mechanisms in Mesenchymal Stem Cells. Stem cells. *22*, 849-860.

Hoogduijn, M.J., Gorjup, E., and Genever, P.G. (2006). Comparative Characterization of Hair Follicle Dermal Stem Cells and Bone Marrow Mesenchymal Stem Cells. Stem cells and development. *15*, 49-60.

Johnson, K., Zhu, S., Tremblay, M.S., Payette, J.N., Wang, J., Bouchez, L.C., Meeusen, S., Althage, A., Cho, C.Y., Wu, X., *et al.* (2012). A Stem Cell-Based Approach to Cartilage Repair. Science. *336*, 717-721.

Repass, J.F., Laurent, M.N., Carter, C., Reizis, B., Bedford, M.T., Cardenas, K., Narang, P., Coles, M., and Richie, E.R. (2009). Il7-Hcd25 and Il7-Cre Bac Transgenic Mouse Lines: New Tools for Analysis of Il-7 Expressing Cells. Genesis. *47*, 281-287.

Saleh, F.A., Frith, J.E., Lee, J.A., and Genever, P.G. (2012). Three-Dimensional in Vitro Culture Techniques for Mesenchymal Stem Cells. Methods in molecular biology. *916*, 31-45.

Saleh, F.A., Whyte, M., Ashton, P., and Genever, P.G. (2011). Regulation of Mesenchymal Stem Cell Activity by Endothelial Cells. Stem cells and development. *20*, 391-403.

Wirth, H., Loffler, M., von Bergen, M., and Binder, H. (2011). Expression Cartography of Human Tissues Using Self Organizing Maps. BMC bioinformatics. *12*, 306.
