# Supplementary material for: Multiparameter Analysis of Human Bone Marrow Stromal Cells Identifies Distinct Immunomodulatory and Differentiation-Competent Subtypes
Source: Stem Cell Reports. 2015 Jun 9;4(6):1004–15. doi: 10.1016/j.stemcr.2015.05.005 (PMC4471830; doi:10.1016/j.stemcr.2015.05.005)
Supplement: Document S2. Article plus Supplemental Information [file mmc2.pdf]

Stem Cell Reports
Report
OPEN ACCESS

Multiparameter Analysis of Human Bone Marrow Stromal Cells Identifies Distinct Immunomodulatory and Differentiation-Competent Subtypes

Sally James,[1,5] James Fox,[1,5] Farinaz Afsari,[1] Jennifer Lee,[1] Sally Clough,[1] Charlotte Knight,[1] James Ashmore,[1] Peter Ashton,[1] Olivier Preham,[1] Martin Hoogduijn,[2] Raquel De Almeida Rocha Ponzoni,[3] Y. Hancock,[3,4] Mark Coles,[1] and Paul Genever[1,*]
[1]Department of Biology, University of York, York YO10 5DD, UK
[2]Erasmus Medical Centre, Dr. Molewaterplein 50, Rotterdam 3015 GE, the Netherlands
[3]Department of Physics, University of York, York YO10 5DD, UK
[4]York Centre for Complex Systems Analysis, University of York, York YO10 5GE, UK
[5]Co-first author
*Correspondence: paul.genever@york.ac.uk
http://dx.doi.org/10.1016/j.stemcr.2015.05.005
This is an open access article under the CC BY license (http://creativecommons.org/licenses/by/4.0/).

SUMMARY

Bone marrow stromal cells (BMSCs, also called bone-marrow-derived mesenchymal stromal cells) provide hematopoietic support and immunoregulation and contain a stem cell fraction capable of skeletogenic differentiation. We used immortalized human BMSC clonal lines for multi-level analysis of functional markers for BMSC subsets. All clones expressed typical BMSC cell-surface antigens; however, clones with trilineage differentiation capacity exhibited enhanced vascular interaction gene sets, whereas non-differentiating clones were uniquely CD317 positive with significantly enriched immunomodulatory transcriptional networks and high IL-7 production. IL-7 lineage tracing and CD317 immunolocalization confirmed the existence of a rare non-differentiating BMSC subtype, distinct from *Cxcl12*-DsRed$^+$ perivascular stromal cells in vivo. Colony-forming CD317$^+$ IL-7^{hi} cells, identified at ~1%–3% frequency in heterogeneous human BMSC fractions, were found to have the same biomolecular profile as non-differentiating BMSC clones using Raman spectroscopy. Distinct functional identities can be assigned to BMSC subpopulations, which are likely to have specific roles in immune control, lymphopoiesis, and bone homeostasis.

INTRODUCTION

Bone marrow stromal cells (BMSCs, also called bone-marrow-derived mesenchymal stromal cells) are heterogeneous populations that likely contain varying levels of tripotent (osteogenic, adipogenic, and chondrogenic [OAC]) stem-cell-like cells; cells with restricted potency (bi-, uni-, and nullipotent), committed precursors, and other stromal cell types. Phenotypic variations probably reflect in vivo functional diversity and a biological requirement for distinct stromal subsets with specific roles in bone marrow maintenance. Single-cell-derived BMSC clone analysis has identified considerable variation in differentiation capacity, ranging from OAC tripotency to nullipotency in vitro (Muraglia et al., 2000; Okamoto et al., 2002; Russell et al., 2010, 2011) and in vivo (Kuznetsov et al., 1997), which may indicate the existence of BMSC subtypes with varied potencies and/or a hierarchical developmental progression. BMSCs also possess significant immunomodulatory characteristics and can influence all aspects of immune cell function via cell-cell interaction and immunoregulatory factor secretion (Nauta and Fibbe, 2007), although clear demarcation between the skeletogenic and immunomodulatory capacity of BMSCs has not been made. Identification of human BMSCs often relies on non-discriminatory epitope detection (such as CD29, CD44, CD73, CD90 [THY-1], CD105, CD106 [VCAM-1], and CD166) with lack of hematopoietic markers (such as CD34, CD14, and CD45) (Dominici et al., 2006). Additional BMSC surface antigens have been described, including STRO-1 (Simmons and Torok-Storb, 1991), CD146 (MCAM) (Sacchetti et al., 2007), CD271 (LNGFR) (Quirici et al., 2002), Nestin (Méndez-Ferrer et al., 2010), platelet-derived growth factor receptor alpha (PDGFRα)/CD51 (Pinho et al., 2013), LNGFR$^+$THY-1$^+$VCAM-1^{hi+} (Mabuchi et al., 2013), and in mice, leptin receptor (LepR/CD295) (Zhou et al., 2014). However, cell-sorting experiments using these markers all show that they contain the colony-forming, differentiation-competent BMSC population, demonstrating that other, as yet undefined BMSC subtypes exist and further resolution of BMSC heterogeneity is required. In vitro studies of BMSC functionality are hindered by their limited lifespan as replicative senescence occurs during culture, thus limiting the number and depth of studies that can be performed. To address these issues, we immortalized human BMSCs using human telomerase reverse transcriptase (hTERT), followed by clonal isolation to generate a panel of BMSC lines (hTERT-BMSCs). This strategy enabled in-depth, multiparameter analysis of variation in human BMSC subpopulations with different behavioral traits that subsequently could be examined in heterogeneous primary BMSCs. We unveil a range of biophysico-chemical markers

for identification of different BMSC subsets with specific immunomodulatory and differentiation competencies.

RESULTS

Generation and Phenotyping of Variant hTERT Immortalized Clonal Cell Lines from Primary BMSCs

A heterogeneous population of primary bone marrow BMSCs was isolated from one donor (FH181) based on their strong growth properties and multilineage differentiation potential. A single donor was deliberately selected to guard against expected genetic/lifestyle factors that cause inter-donor variation and would cloud interpretation of BMSC sub-population variation. A lentiviral expression system was used to overexpress hTERT in these cells, and stable-expressing lines were generated from single-cell-derived colonies. From numerous hTERT-BMSCs generated, our attention focused initially on four clonal lines selected for their strong clonal and stable growth characteristics (termed Y101, Y102, Y201, and Y202) that were maintained for over 400 days, showing exponential growth (Figure S1A) and elevated telomerase activity (Figure S1B). Their BMSC marker profile was determined by flow cytometry; all were positive for CD29, CD44, CD73, CD90, CD105, and CD166 and negative for CD34 and CD45 (Figure 1A), a profile matching their parental cells and typical of human primary BMSCs. All clones were also negative for CD11b, CD14, CD169, CD1a, CD4, CD11c, and CD83 (data not shown). It has been suggested previously that some BMSC lines may undergo oncogenic transformation either through hTERT transduction or extended time in culture (Serakinci et al., 2004). We found no evidence of tumorigenicity in our hTERT-BMSCs using in vitro colony transformation assays for non-adherent growth (Figure S1C) or following subcutaneous injection into immunocompromised mice (data not shown). Distinct differences were observed in individual cell morphologies and single-cell-derived colonies: Y101 and Y201 shared characteristics that were distinct from Y102 and Y202 lines. Y101 and Y201 lines had elongated fibroblastoid morphology, typical of BMSCs, whereas Y102 and Y202 were flattened and spread. When plated at clonal density, Y101/Y201 cells consistently formed dispersed colonies with low cell contact, characteristic of a migratory phenotype, whereas Y102 and Y202 cells formed regular, high-density, compact colonies (Figures 1B and S1D). Morphological differences were quantified by image analysis, which demonstrated that Y202 cells had a statistically significantly greater area and perimeter compared to both the Y101 and Y201 cells. Y102 cells were also larger than the Y101/Y201; the mean cell perimeters for Y102, Y202, Y101, and Y201 were 273 ± 23, 317 ± 107, 202 ± 19, and 190 ± 34 μm, and mean cell areas were 1,537 ± 132, 1,649 ± 614, 1,067 ± 92, and 857 ± 214 μm^2, respectively (Figures S1E and S1F).

We identified variations in potency following OAC differentiation assays (Figures 1C–1E). Adipogenic media for up to 21 days induced the most potent observable and statistically significant adipogenic differentiation in Y201 cells at each time point, measured using the oil red O lipid-staining assay (Figure 1C). Y101 was also observed to differentiate into adipocytes, reaching significance at day 21, but Y102 and Y202 showed severely limited, although not completely absent, adipogenic potential, which was significantly different to Y201 at day 21. In addition, there were statistically significant increases in the levels of expression of the adipogenic genes peroxisome proliferator-activated receptor (PPAR)-γ and lipoprotein lipase (LPL) in the Y201 cells cultured in adipogenic media at day 14 and day 21 compared to the time-matched untreated controls. At day 21, the expression level of both these genes was significantly lower in the three other cell lines (Figure 1C). Y101 and Y201 differentiated convincingly along osteogenic lineages and showed elevated alkaline phosphatase (ALP) activity with significantly increased calcium deposition by alizarin red staining (Figure 1D), which was statistically higher than Y102/Y202 clones, which showed minimal mineral deposition, although Y102 displayed elevated ALP activity (Figure 1D). Increases in ALP activity observed under basal conditions for Y101 and Y201 were replicated in qPCR analyses of the ALP gene (Figure S1G); further increases in ALP gene expression were clearly evident in the Y101 cell line under osteogenic conditions, while the levels in the other lines only showed modest increases. Furthermore, histological staining for elevated ALP activity was only observable in the Y101/Y201 cells (Figure S1G). Gene expression analyses for *RUNX2* showed that only the Y101/Y201 cell lines upregulated this early osteogenic marker at day 7 (Figure 1D). In response to chondrogenic induction using transforming growth factor β (TGF-β), only lines Y101 and Y201 displayed an early cell-condensation phenotype associated with chondrogenic induction (Johnson et al., 2012), whereas no condensation was observed in similar micromass culture of lines Y102 and Y202 (Figure 1E). When cultures were stained with alcian blue and stain-associated glycosaminoglycans (GAGs) were eluted and quantified, significantly different levels were observed in all cell lines at day 9 compared to their time-matched controls under basal conditions, but the highest and statistically significant GAG levels were clearly evident in the Y101/Y201 compared to the Y102/Y202 cell lines. Similarly, only the Y101 and Y201 lines displayed marked increases in total GAG production under chondrogenic conditions in the Blyscan GAG assay (Figure 1E). Gene expression analyses

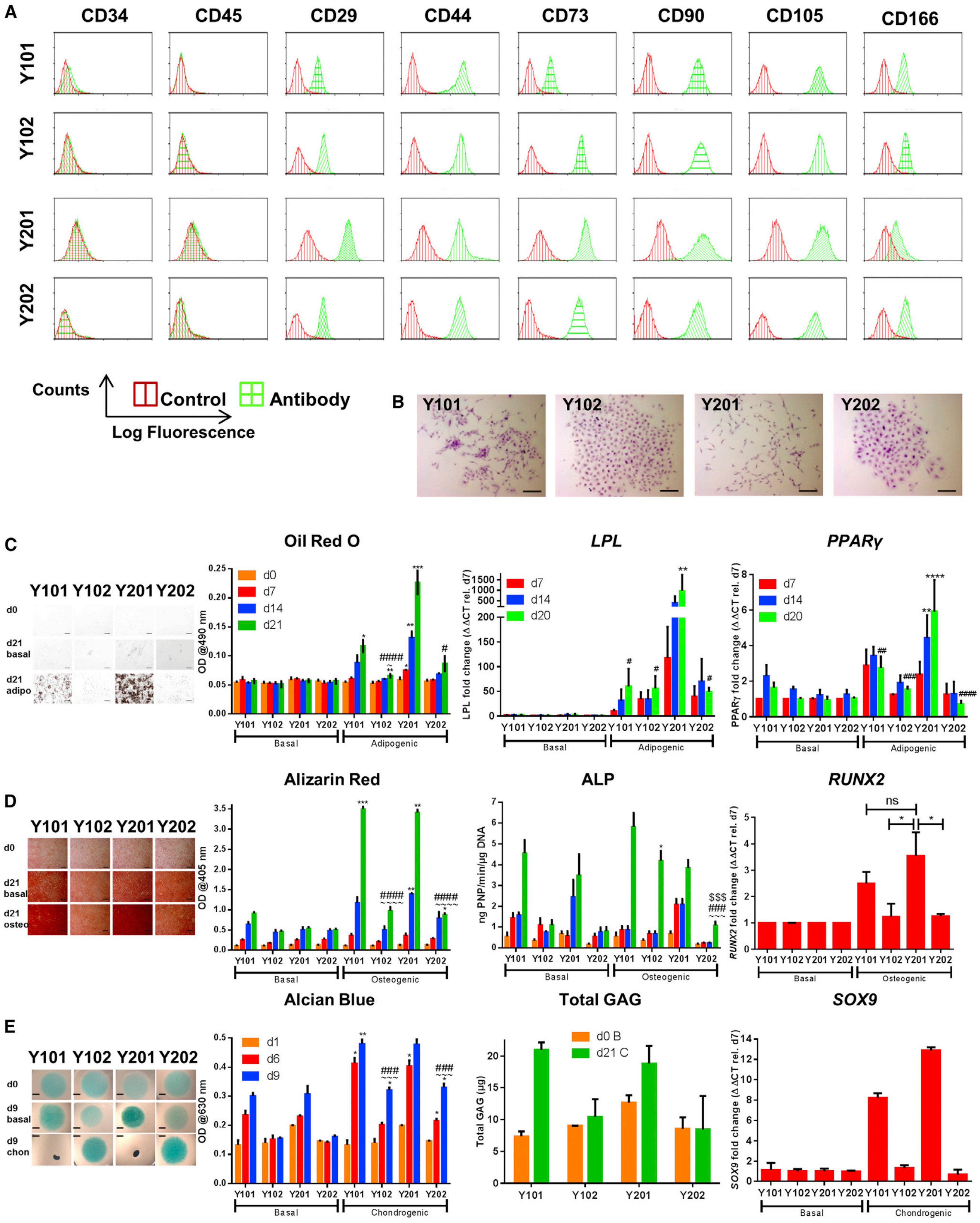

(legend on next page)

for the early chondrogenic marker *SOX9* confirmed that this marker only increased in the Y101/Y201 cell lines after 7 days in chondrogenic differentiation media (Figure 1E). Therefore, lines Y101/Y201 were capable of BMSC OAC differentiation, with Y201 showing stronger adipogenesis. The Y102/Y202 BMSC lines exhibited a non-hematopoietic, stromal phenotype with distinctive clonal behavior but with atypical BMSC morphology and limited differentiation ability compared to Y101/Y201.

Interrogation of Gene Expression Profiles Identifies Immune-Related BMSC Subtypes

We performed global gene expression analyses to identify distinguishing characteristics between the hTERT-BMSC clones and parental BMSCs; selecting significantly differentially expressed genes ($p < 0.05$, >2-fold change). Hierarchical clustering grouped Y101 with Y201 and closest to the primary parent BMSCs, while Y102/Y202 clustered separately (Figure 2A). Principal component analysis (PCA) revealed that 70% of the total variance was captured by the first two components (Figure S2A) and confirmed segregation of Y102/Y202 BMSC lines from the Y101/Y201 BMSC lines, the parent BMSC (FH181), and other primary BMSC populations (Figure S2B). Pathway analysis identified significant differences in expression of genes involved in the cell cycle, DNA replication, and other processes associated with cell replication, as would be predicted when comparing hTERT-immortalized lines with the parent sample (Figure 2B). Additional gene sets identified were involved in cell adhesion, endochondral ossification, and adipogenesis. Differentially expressed genes were notably enriched in pathways involved in Toll-like receptor, interferon, tumor necrosis factor α (TNF-α), interleukin-7 (IL-7) signaling, and inflammatory responses, implicating potential differences in the immunoregulatory properties between these lines (Figure 2B). Patterns of gene expression were also visualized and investigated using self-organizing heatmaps (SOMs). Expression data were used to produce mosaic fingerprints, and each mosaic tile represents metagenes that consist of mini-clusters of genes with similar expression placed in the same position across the mosaics (Figure 2C). Y101, Y201, Y102, and Y202 were compared against the primary parent BMSC source (FH181) and four others (FH469, FH348, FH359, and FH392). Primary BMSCs showed consistent spots of strong over- and under-expression in the bottom-right and top-left corners, respectively. Y101/Y201 and Y102/Y202 hTERT-BMSCs showed differences in patterns of expression at regions of over- and under-expression in the top right and bottom left of the SOMs, respectively (Figure 2C, arrows, and Figures S2C and S2D). The most significant gene sets overexpressed in Y101/201 versus Y102/202 were related to vascular growth (blood vessel remodelling, blood vessel development, artery morphogenesis, and patterning of blood vessels; Figure S2C). Under-expressed Y101/Y201 gene sets that were most significantly over-represented in Y102/Y202 lines were immunomodulatory (antigen processing, MHC class II proteins, T cell signaling, and responses to interferon; Figure S2D).

Further analysis found a strikingly elevated endogenous expression of inflammation-induced genes in the non-differentiating lines Y102 and Y202 compared to the OAC-differentiation-competent BMSC clones (Figure 2D). By analyzing gene expression changes reported in a previously published dataset, where global gene expression was studied in adipose-derived BMSCs with or without stimulation with inflammatory cytokines (interferon γ [IFN-γ], TNF-α, and IL-6) (Crop et al., 2010), we found that inflammation-induced genes were also elevated in unstimulated Y102 and Y202 cells compared to the Y101/Y201 and parental BMSCs (Figure 2D). These observations pointed to the existence of a non-differentiating, resident stromal cell subset with "unlicensed" immunoregulatory potential associated with a pro-inflammatory response. The immunomodulatory effects of BMSCs are normally reported to be related to immunosuppression. In assays of immunosuppressive function, we found that all hTERT-BMSC lines were similarly capable of inhibiting anti-CD3/anti-CD28 antibody-stimulated peripheral blood mononuclear cell (PBMC) proliferation with no significant

Figure 1. Generation and Analysis of hTERT-BMSC Clones

(A) Analysis of hTERT-BMSC clones (Y101, Y102, Y201, and Y202) by flow cytometry for surface markers typically assigned to BMSCs. Representative histograms are shown.

(B) Clonal growth of hTERT-BMSCs (crystal violet stain). Scale bars, 200 μm.

(C–E) Assessment of the OAC potential of hTERT-BMSC clones; representative images and quantification of cells differentiated with adipogenic (C), osteogenic (D), and chondrogenic supplements (E). Scale bar represents 200 μm (C and D) or 2 mm (E). Data represent average quantified values ± SD for two or three independent experiments performed using two to six replicates. Differentiation was considered to be statistically significant after the data from $n > 3$ experiments were analyzed by one-way ANOVA with Holm Sidak's multiple comparisons if *$p < 0.05$, **$p < 0.01$, ***$p < 0.001$, or ****$p < 0.0001$ between both day 0 and the time-matched basal control. Additionally, one-way ANOVA with Holm Sidak's comparisons was performed between the different cell lines at day 21, and significant differences compared with Y101 (~) or Y102 ($) and Y201 (#) are indicated.

See also Figure S1.

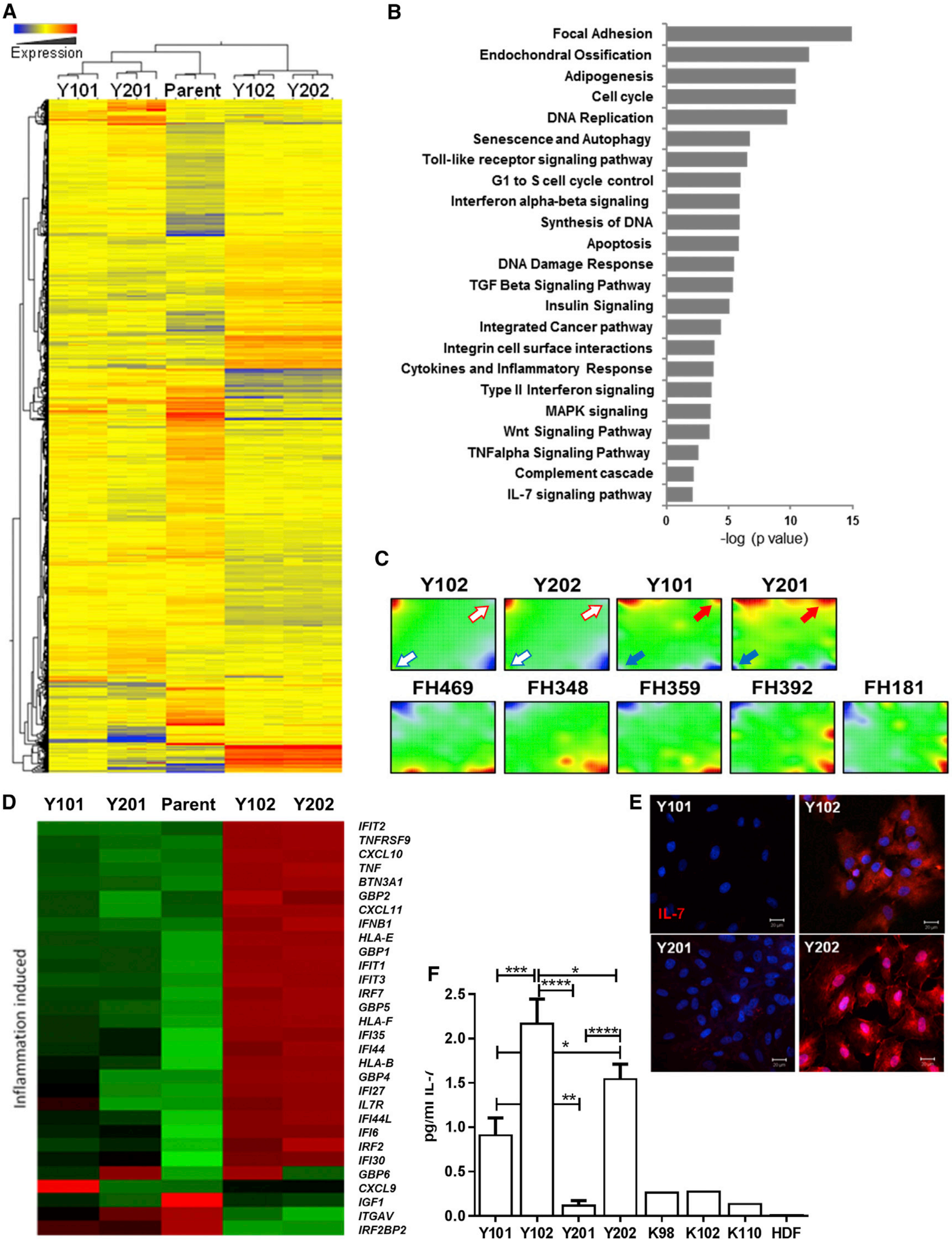

Figure 2. Expression Profiling Identifies BMSC Clones with Distinct Immunoregulatory Features

(A) Hierarchical clustering heatmap of global gene expression profiles from each hTERT-BMSC clonal line and the parental BMSCs.

(B) Pathway analysis showing most significantly enriched pathways.

(C) Self-organizing heatmaps of the four hTERT-BMSC clones and five primary BMSC samples, including the parent population (FH181). Arrows indicate presence (closed) and absence (open) of overrepresented (red) and underrepresented (blue) metagene spots.

(D) Heatmap showing expression of key genes involved in inflammatory-induced responses.

(legend continued on next page)

differences of the inhibitory capacity of the cell lines at each BMSC:PBMC ratio used (Figure S2E). Similar increases in the expression of immunosuppressive factors (*IDO1*, *TGFB1*, and *CD274*) following exposure to TNF-α and IFN-γ were observed in all hTERT-BMSC lines, but *IL6* expression was significantly higher in the Y201 cells under TNF-α/IFN-γ stimulation (Figure S2F). Y102/Y202 cells were significantly more responsive to inflammatory cytokine-induced expression of factors involved in lymphocyte homing and development, CXCL10 and IL-7. In particular, basal mRNA expression of *IL-7* in Y102/Y202 cells was equivalent to expression levels in TNF-α/IFN-γ-stimulated Y101/Y201 cells (Figure S2F). IL-7 signaling was highlighted as a differentially regulated pathway (Figure 2B), and we identified strong expression of IL-7 in Y102/Y102 compared to low IL-7 levels in Y101/Y201 by immunocytochemistry (Figure 2E). Using ELISAs, we confirmed significantly higher secretion of IL-7 in Y102/202 versus Y101/Y201; heterogeneous primary BMSCs secreted low levels of IL-7, with negligible expression in human dermal fibroblasts (Figure 2F). No difference in IL-7 receptor expression level was observed by flow cytometry across the four cell lines (data not shown).

Together, these data indicate that OAC-competent BMSC clones expressed an extensive gene set consistent with vascular interaction; all BMSC lines showed broadly similar immunosuppressive features, while the poorly differentiating hTERT-BMSCs displayed a striking constitutive immunostimulatory expression profile and could be identified by elevated basal IL-7 expression, which has important functions in lymphopoiesis (Ceredig and Rolink, 2012).

In Vivo Lineage Tracing of IL-7-Expressing Stromal Cells

To identify and track IL-7-positive BMSC subpopulations in vivo, we used an IL-7Cre Rosa26-EYFP lineage-tracing mouse model, where enhanced yellow fluorescent protein (EYFP) expression persists in the progeny of cells that expressed IL-7 at any stage. EYFP-positive cells were found in bone marrow occasionally at perivascular and endosteal-lining locations but broadly distributed throughout the marrow with an ~4% frequency (Figures 3A–3F and S3A). Using flow cytometry to discriminate CD45-expressing hematopoietic cells, we found the EYFP-positive stromal fraction represented ~0.9% of the marrow cell population (Figure S3B). From histological sections, 91%, 77%, and 92%, respectively, of femoral, tibial, and calvarial osteocytes, terminally differentiated cells of the osteogenic lineage (Figures 3D–3F and S3A), and all perilipin-positive bone marrow cells and all adipocytes in white adipose tissue examined were EYFP negative (Figures 3G–3L and S3C). Strong EYFP positivity was confirmed in 11%–60% of articular chondrocytes (Figures 3M–3O and S3A), where IL-7 expression has been previously identified (Long et al., 2008), and in 7%–31% of hypertrophic chondrocytes (Figure S3A). These data demonstrate that a substantial proportion of mesodermal-derived adipose, bone, and cartilage tissues originate from a progenitor cell type that at no point expressed IL-7 and support the existence of poorly/non-differentiating IL-7^{hi} BMSC subtype in vivo.

Identification of Y102/Y202-Related Subtypes in Primary BMSCs

Y101/Y201 and Y102/Y202 lines were screened by flow cytometry in an attempt to identify a distinguishing cell-surface protein to sort from primary BMSC populations. We found little difference in expression of candidate BMSC markers CD271, CD51, and PDGFRα; while CD146 was largely undetectable on the Y201 line, its expression was similar in the other BMSC clones (Figure 4A). LepR/CD295 was expressed by both OAC-differentiating lines and the differentiation-incompetent lines (Figure 4A); in murine bone marrow, LepR/CD295 expression was found to be predominantly, though not exclusively, expressed by bone- and adipocyte-forming BMSCs (Zhou et al., 2014).

We analyzed the array data for differential expression of genes encoding cell-surface proteins; while ICAM1 and CD274 had similar protein expression profiles across all lines, CD317 was selectively and highly expressed in Y102 and Y202 cells and absent from Y101 and Y201 cells (Figure 4A). We examined another four BMSC clones (Y204, Y205, Y301, and Y302) generated during the initial hTERT immortalization experiments (Figure S4A). They exhibited varying degrees of differentiation potency (Figures S4B–S4H), but none were differentiation incompetent across osteogenic, adipogenic, and chondrogenic lineages to the same degree as observed for Y102/Y202, and all were negative for CD317 (Figure S4I). CD317 (also known as BST2/tetherin) has recognized anti-viral properties (Hotter et al., 2013), and

(E) Immunocytochemical detection of IL-7 expression (red) by the four hTERT-BMSC clones (after 24 hr in culture) (DAPI nuclear counterstain in blue). Scale bars, 20 μm.
(F) IL-7 expression by ELISA in the four hTERT-BMSC clones, primary BMSCs (K98, K102, and K110), and human dermal fibroblasts (HDF). Data represent mean values ± SD from three individual experiments performed in duplicate.
See also Figure S2.

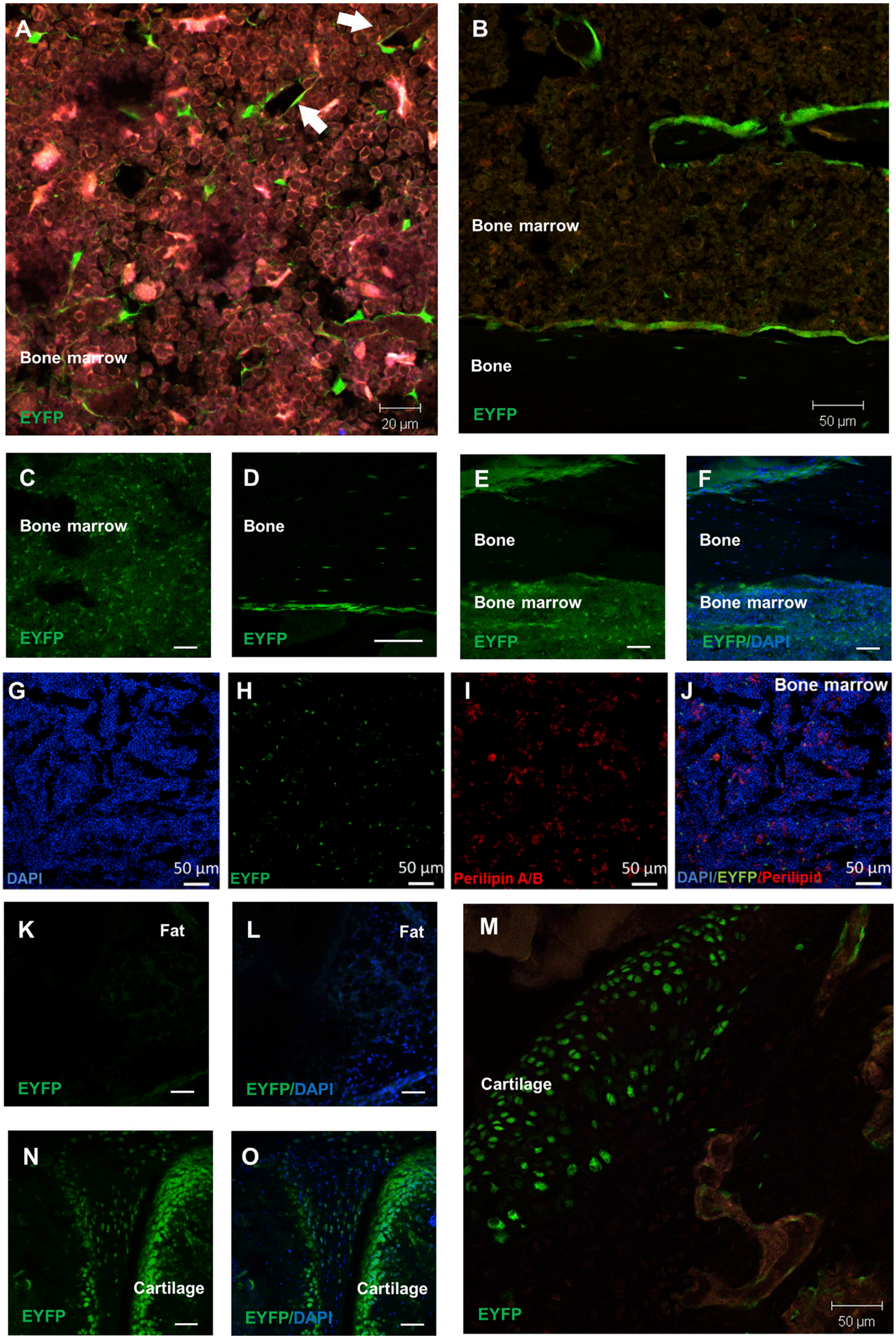

(legend on next page)

Y102/Y202 cells expressed elevated levels of several other anti-viral genes compared to Y101/Y201 and parent BMSCs (Figure 4B).

In low passage, primary BMSCs, a CD317-expressing population was detected in ~1%–3% of the total cells (Figure S4J). We used fluorescence-activated cell sorting (FACS) to sort the $CD317^{+}$ fraction from $CD317^{-}$ cells for further analysis and found that $CD317^{+}$ cells were capable of density-independent growth and formed CFU fibroblasts (CFU-F) at a frequency similar to $CD317^{-}$ cells (Figure 4C). Morphological analysis of individual colonies identified that $CD317^{+}$ cells had statistically significantly increased cell perimeters (1,042 ± 309 μm) and areas (5,355 ± 1,306 μm) compared to $CD317^{-}$ cells (1,386 ± 638 μm and 330 ± 154 μm, respectively) (Figures 4D and S4K), mirroring Y102/Y202 versus Y102/Y201 size differences. Using qPCR and ELISA, we confirmed that $CD317^{+}$ BMSCs expressed significantly elevated IL-7 mRNA and secreted more IL-7 protein than the $CD317^{-}$ population (Figure 4E), with limited evidence that $CD317^{+}$ cells have elevated basal mRNA expression levels of immunosuppressive factors compared to $CD317^{-}$ cells (Figure S4L), while exposure of primary BMSCs to IFN-γ for 24 hr resulted in an ~3-fold increase in CD317 expression (Figure S4M). As marker expression can change with time in culture, we also sorted CD317-expressing cells from fresh, whole human bone marrow mononuclear cells (BM-MNCs); $CD317^{+}$ cells in the stromal $CD45^{-}CD31^{-}$ population represented on average 0.4% of the live cell population from two donors (Figures S4N and S4O).

In mouse tissue sections, CD317 was expressed sporadically throughout the bone marrow (Figures 4F and S4P; isotype control is shown in Figure S4Q). CD317-positive cells rarely associated with CD31-positive vascular cells (Figures 4F and S4P) and did not generally colocalize with *Cxcl12*-$DsRed^{+}$ perivascular stromal and endothelial cells (Figure 4F). The majority of bone marrow LepR-expressing cells lacked CD317 expression, though occasional $LepR^{+}CD317^{+}$ cells were identified (Figure 4G).

Collectively, these findings identify the presence of a rare, colony-forming population of BMSCs, marked by CD317 positivity, which appear to have an enhanced immunostimulatory capacity and are closely related to the Y102 and Y202 phenotype. We employed Raman spectroscopy to determine the extent of this relationship and provide a diagnostic evaluation of functional variation between BMSC subtypes. Averaged spectra were generated for each hTERT clonal line and primary $CD317^{+}$ sorted BMSCs (Figure 4H), with their respective peak assignments identified as described previously (Movasaghi et al., 2007; Schulze et al., 2010). Peak intensity ratios were obtained between all identified peaks for each cell type; the most discriminatory was found to be against the 1,088.6 cm^{-1} peaks. The 1,088.6 cm^{-1} peak is related to the symmetric phosphate stretch of the DNA backbone (Movasaghi et al., 2007) with its discrimination signifying a fundamental difference in the DNA of the cell types. This peak has also been used to discriminate other cell types, (Chan et al., 2006). The 966cm^{-1}/1088.6 cm^{-1} peak intensity ratios distinguished Y101 from Y201 (Figure 4I, arrow). Other peak intensity ratios, most notably from 999.6 cm^{-1} and higher, clearly separated Y101/Y201 from Y102/Y202 (Figure 4I). Importantly, primary $CD317^{+}$ cells mapped with remarkable consistency to the Y102/Y202 Raman profile (Figure 4I), providing strong evidence of relatedness.

DISCUSSION

We generated immortalized BMSC clones to perform detailed analysis of functional diversity in BMSC subtypes to levels not previously described. Similar immortalization techniques (e.g., hTERT combined with human papillomavirus E6/E7) have been used for the clonal analysis of BMSCs, which found OAC, AO, OC, unipotent, and nullipotent clones at varied frequencies, but other functional characteristics and discriminating features were not examined (Larsen et al., 2010; Okamoto et al., 2002). Notably, however, enhanced immune-related features could be assigned to non-bone-forming hTERT-BMSCs in a previous study (Larsen et al., 2010). Using non-immortalized BMSC clones, Muraglia et al. identified OAC-, OC-, and O-competent lines only, with very rare (<1%) nullipotent subsets (Muraglia et al., 2000). Rather than determine occurrence of O/A/C phenotypes in heterogeneous BMSCs, here our aim was to identify molecular and biophysical markers of BMSC subtypes and their alignment with

Figure 3. Lineage Tracing of IL-7-Expressing Cells Using IL-7cre Rosa26-EYFP Mice

(A–F) Sporadic EYFP expression throughout bone marrow, associated occasionally with cells lining the vasculature (A, arrows) and prominent on endosteal surfaces (B). Infrequent osteocytes were EYFP positive (D–F).

(G–J) Immunostaining of perilipin-positive adipocytes (red) in bone marrow of IL-7cre Rosa26-EYFP mice.

(K and L) Absence of EYFP expression in white adipose tissue extracted from IL-7cre Rosa26-EYFP mice.

(M–O) EYFP expression in chondrocytes of articular cartilage.

Scale bars, 50 μm (unless otherwise stated). See also Figure S3.

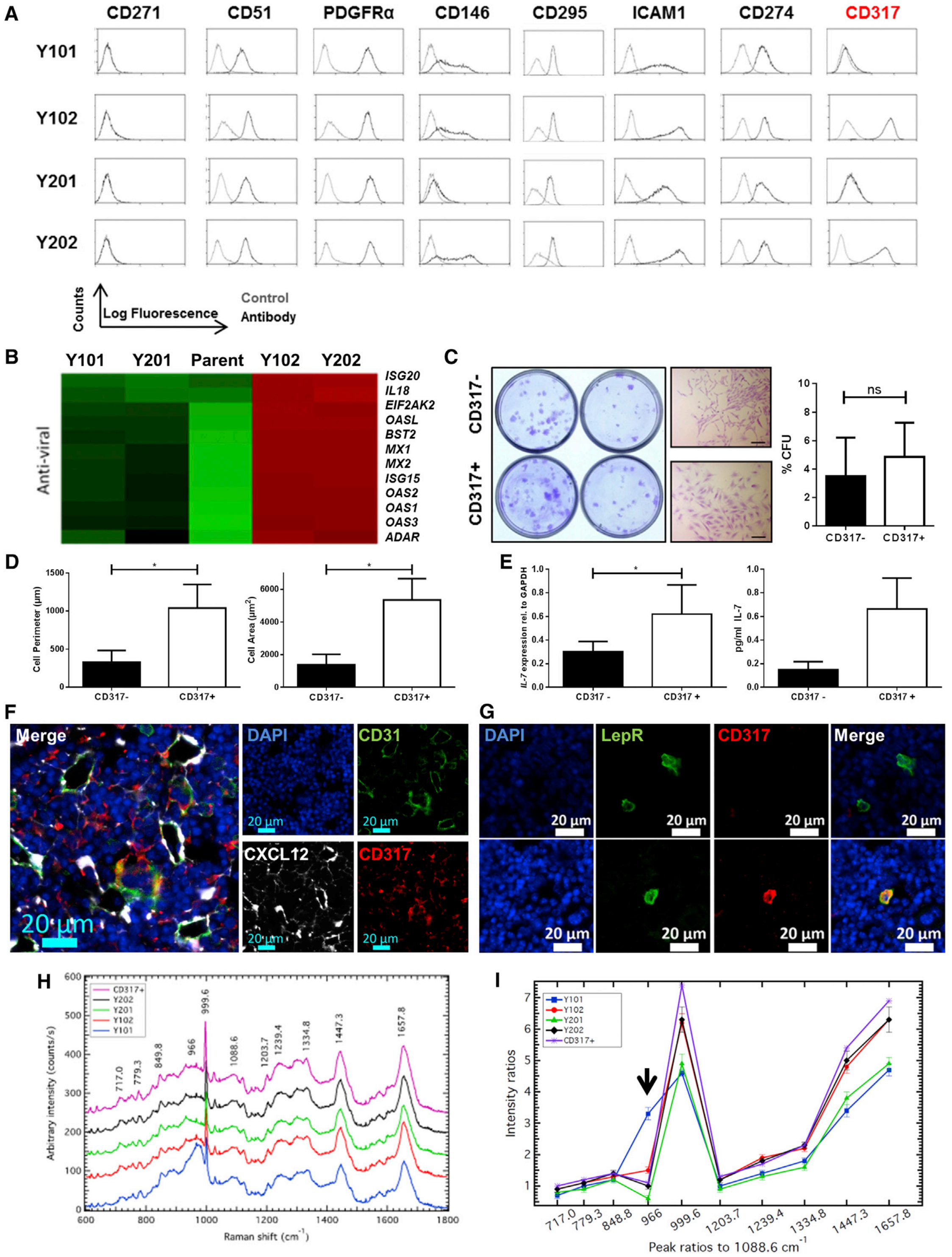

Figure 4. Identification of CD317$^+$ Bone Marrow Cells

(A) Flow cytometric analysis of hTERT-BMSC; CD317 expression selectively discriminates Y102/Y202 from Y101/Y201.

(B) Heatmap showing expression of key genes involved in anti-viral responses.

(C) CFU-F assay and morphology of CD317$^-$ and CD317$^+$ cells sorted from heterogeneous BMSCs. Scale bars, 200 μm. Histogram represents mean ± SEM (n = 4 independent experiments performed with five replicates).

(legend continued on next page)

non-progenitor cell functions, including immunomodulatory properties. To this end, from the original hTERT-BMSC lines generated, we selected four clones positioned at opposing ends of a differentiation competency scale, tripotent Y101/Y201 and relatively impotent Y102/Y202, for in-depth cell profiling. The OAC-differentiating lines expressed an extensive and significantly enriched gene set consistent with blood vessel support/interaction compared to nullipotent clones. This may reflect a relationship between BMSC differentiation competency and a perivascular niche location (Sacchetti et al., 2007). Conversely, Y102 and Y202 lines expressed CD antigens widely used to describe BMSCs (Dominici et al., 2006; Pittenger et al., 1999) but exhibited very little differentiation capacity. Subsequent assays revealed that an equivalent $CD317^+$ cell type existed in heterogeneous primary BMSC populations at ~1%–3% frequency and $CD45^-CD31^-CD317^+$ cells represented ~0.4% of whole human BM-MNCs. Primary cell analysis and in vivo validation is an important component of our study. Data obtained using in vitro immortalized cell lines must be interpreted with some caution, considering the effects of hTERT transduction artifacts. Integration site, enhanced telomerase activity and long-term culture are all likely to impact on cell function. We attempted to minimize potential confounding effects by selecting two cell lines, each displaying similar properties (Y101/Y201 and Y102/Y202). We used Raman spectroscopy, which has been demonstrated as a non-destructive label-free method to study the biological state of single cells and to identify molecular-scale differences for cell discrimination (see Schulze et al., 2010, for example). In this work, Raman spectroscopy was able to uniquely identify the four hTERT-BMSC clones, separating Y101 from Y201 and confirming a striking biomolecular relationship between Y102/Y202 and primary $CD317^+$ cells, which also shared an enlarged, spread morphology, forming compact, non-migratory colonies. We performed detailed bioinformatics to scrutinize gene expression profiles against the parental BMSCs. These analyses ultimately identified the closely clustered Y102 and Y202 lines as having remarkably high basal level expression of factors associated with a pro-inflammatory and anti-viral function. The significantly enriched gene sets we identified in unchallenged Y102/Y202 BMSC lines are typical of an immediate immunostimulatory response to infection and other insults. Notably, all BMSC clones exhibited similar inhibitory effects on PBMC proliferation when activated by exposure to anti-CD3 and anti-CD28 to mimic antigen presentation. Consequently, Y102/Y202 BMSC subtypes may function in both immunostimulatory (early, pro-inflammatory) and immunosuppressive (anti-inflammatory, tissue protective) responses depending on the immune environment; however, functional validation and further refinement of the characteristics of this CD317-expressing population is required. Dual immunomodulatory functions for BMSCs have been identified and discussed (Dazzi and Krampera, 2011), but not assigned to BMSC subtypes. $CD317^+$ cells (as well as Y102/Y202) were characterized as having elevated IL-7 expression, which has well-established roles in T and B cell development (Ceredig and Rolink, 2012). Examination of skeletal tissues in a lineage-tracing mouse model confirmed that a substantial subpopulation of the differentiated progeny of BMSCs could not have arisen from IL-7-expressing stromal progenitors. It should be noted that differentiated skeletal cells may express IL-7 (Long et al., 2008; Zhu et al., 2007), which would also be revealed as EYFP positivity. However, these studies provide unequivocal evidence linking IL-7 expression in stromal subsets with differentiation incompetence during normal development in vivo. Our in vivo observations were reflected in our in vitro assays of differentiation, where the IL-7^{hi} immortalized BMSC clones (Y102/Y202) exhibited limited skeletogenic potential. These in vitro assays are prone to artifact that could result in non-specific detection of widely used markers of differentiation that may not report authentic in vivo differentiation capacity, observed for example using in vivo transplantation techniques (Robey et al., 2014). Even with these caveats, we still failed to detect clear evidence of differentiation ability in Y102/Y202 $CD317^+$ IL-7^{hi} BMSC lines. In vivo, $CD317^+$ cells had a dispersed bone marrow distribution and did not generally colocalize with *Cxcl12*-$DsRed^+$ perivascular

(D) Analysis of cell perimeter (left) and cell area (right) of $CD317^-$ and $CD317^+$ cells sorted from heterogeneous BMSCs. Mean ± SEM, n = 4 independent experiments in which, on average, 113 cells were examined. *$p < 0.05$ by unpaired non-parametric t test.
(E) IL-7 mRNA (left) and protein expression (right, by ELISA) in $CD317^-$ and $CD317^+$ cells. Data represent mean IL-7 levels ± SD from FACS-sorted cells in triplicate from four or two donors, respectively. *$p < 0.05$ by paired t test.
(F) Immunohistochemistry of mouse femur bone marrow sections stained with antibodies to CD317 (red), CD31 (green) with *Cxcl12*-DsRed (white) and nuclear (DAPI, blue) staining. Representative merged and single-panel images shown.
(G) Immunolocalization of LepR/CD295 (green) and CD317 (red) in mouse bone marrow showing $LepR^+CD317^-$ (upper panel) and occasional dual $LepR^+CD317^+$ cells (lower panel). Blue indicates nuclear DAPI stain.
(H and I) Analysis of hTERT-BMSC clones and $CD317^+$ cells by Raman spectroscopy, Raman shifts (H) and peak ratios (I) are shown. Raman peak assignments are provided in Table S2.
See also Figure S4.

stromal cells, again pointing to an alternative function for CD317⁺ BMSCs, unrelated to CXCL12-mediated hematopoietic stem cell niche support. CD317 has a well-defined anti-viral function acting to tether and restrict the release of viral particles and may also trigger the viral immune response (Hotter et al., 2013). The existence of a marrow-resident, non-migratory, non-differentiating colony-forming stromal cell type, apparently primed for host defense, is an interesting concept. These cells could act as first responders to pathogen invasion and/or provide low-level immune control. As IL-7 has been implicated as a causative factor in autoimmunity, including rheumatoid arthritis (Dooms, 2013), unregulated control of CD317⁺ cell numbers and function may also contribute to disease onset and progression. These findings highlight the importance of identifying unique markers of BMSC sub-populations and assigning function, which will instruct strategies to analyze stromal-immune cell interaction, differentiation competency, and cell selection for safe and effective therapy.

EXPERIMENTAL PROCEDURES

Primary BMSC Isolation, Culture, and hTERT BMSC Production

All animal experiments and work involving human samples were approved by the University of York, Department of Biology ethics committee. Primary human BMSCs were isolated from femoral heads obtained with informed consent during routine hip replacement (see the Supplemental Experimental Procedures) or as explant cultures from human tibial plateaus after routine knee replacement. Cells were cultured in DMEM containing 15% fetal bovine serum, 100 U/ml penicillin, and 100 μg/ml streptomycin (changed every 3–4 days); cells were passaged at 70%–80% confluency. Cells were expanded and used between passage 1 (p1) and p5.

The hTERT lentiviral vector was produced using ViraPower Lentiviral Gateway Expression Kit (Invitrogen), according to the manufacturer's guidelines, before transduction into primary human BMSCs isolated at p3 from the femoral head of a single donor. For selection of single cell lines, the transduced BMSCs were trypsinized and plated at 10 cells/cm^2. Cells were grown in BMSC medium containing 20% HyClone serum at plating, then replaced with fresh media containing 15% HyClone serum every 3 or 4 days to day 14 (until discrete single-cell colonies were visible). Single-cell colonies were isolated and expanded.

For further information, see Supplemental Experimental Procedures.

SUPPLEMENTAL INFORMATION

Supplemental Information includes Supplemental Experimental Procedures, four figures, and three tables and can be found with this article online at http://dx.doi.org/10.1016/j.stemcr.2015.05.005.

AUTHOR CONTRIBUTIONS

S.J. and J.F. performed experiments and designed the studies. F.A., M.H., S.C., J.L., and C.K. performed in vitro experiments; J.A. and P.A. performed the bioinformatics analyses; and O.P., S.C., and M.C. provided and analysed in vivo data. R.D.A.R.P. and Y.H. designed and analysed the Raman work. P.G. supervised the work and designed experiments. All authors contributed to writing the paper, which was led by J.F., S.J., and P.G.

ACKNOWLEDGMENTS

Arthritis Research UK Tissue Engineering Centre (19429), Biotechnology and Biological Sciences Research Council, the Dr. Hadwen Trust and Medical Research Council (G0601156), and a CNPq (Brazil) PhD scholarship funded the work. We thank Florian Gruber for assistance with CellProfiler analysis. We are grateful to staff and patients of Clifton Park Hospital for samples.

Received: July 30, 2014
Revised: May 5, 2015
Accepted: May 5, 2015
Published: June 9, 2015

REFERENCES

Ceredig, R., and Rolink, A.G. (2012). The key role of IL-7 in lymphopoiesis. Semin. Immunol. *24*, 159–164.

Chan, J.W., Taylor, D.S., Zwerdling, T., Lane, S.M., Ihara, K., and Huser, T. (2006). Micro-Raman spectroscopy detects individual neoplastic and normal hematopoietic cells. Biophys. J. *90*, 648–656.

Crop, M.J., Baan, C.C., Korevaar, S.S., Ijzermans, J.N., Pescatori, M., Stubbs, A.P., van Ijcken, W.F., Dahlke, M.H., Eggenhofer, E., Weimar, W., and Hoogduijn, M.J. (2010). Inflammatory conditions affect gene expression and function of human adipose tissue-derived mesenchymal stem cells. Clin. Exp. Immunol. *162*, 474–486.

Dazzi, F., and Krampera, M. (2011). Mesenchymal stem cells and autoimmune diseases. Best Pract. Res. Clin. Haematol. *24*, 49–57.

Dominici, M., Le Blanc, K., Mueller, I., Slaper-Cortenbach, I., Marini, F., Krause, D., Deans, R., Keating, A., Prockop, Dj., and Horwitz, E.; The International Society for Cellular Therapy Position Statement (2006). Minimal criteria for defining multipotent mesenchymal stromal cells. The International Society for Cellular Therapy position statement. Cytotherapy *8*, 315–317.

Dooms, H. (2013). Interleukin-7: Fuel for the autoimmune attack. J. Autoimmun. *45*, 40–48.

Hotter, D., Sauter, D., and Kirchhoff, F. (2013). Emerging role of the host restriction factor tetherin in viral immune sensing. J. Mol. Biol. *425*, 4956–4964.

Johnson, K., Zhu, S., Tremblay, M.S., Payette, J.N., Wang, J., Bouchez, L.C., Meeusen, S., Althage, A., Cho, C.Y., Wu, X., and Schultz, P.G. (2012). A stem cell-based approach to cartilage repair. Science *336*, 717–721.

Kuznetsov, S.A., Krebsbach, P.H., Satomura, K., Kerr, J., Riminucci, M., Benayahu, D., and Robey, P.G. (1997). Single-colony derived

strains of human marrow stromal fibroblasts form bone after transplantation in vivo. J. Bone Miner. Res. *12*, 1335–1347.

Larsen, K.H., Frederiksen, C.M., Burns, J.S., Abdallah, B.M., and Kassem, M. (2010). Identifying a molecular phenotype for bone marrow stromal cells with in vivo bone-forming capacity. J. Bone Miner. Res. *25*, 796–808.

Long, D., Blake, S., Song, X.Y., Lark, M., and Loeser, R.F. (2008). Human articular chondrocytes produce IL-7 and respond to IL-7 with increased production of matrix metalloproteinase-13. Arthritis Res. Ther. *10*, R23.

Mabuchi, Y., Morikawa, S., Harada, S., Niibe, K., Suzuki, S., Renault-Mihara, F., Houlihan, D.D., Akazawa, C., Okano, H., and Matsuzaki, Y. (2013). LNGFR(+)THY-1(+)VCAM-1(hi+) cells reveal functionally distinct subpopulations in mesenchymal stem cells. Stem Cell Reports *1*, 152–165.

Méndez-Ferrer, S., Michurina, T.V., Ferraro, F., Mazloom, A.R., Macarthur, B.D., Lira, S.A., Scadden, D.T., Ma'ayan, A., Enikolopov, G.N., and Frenette, P.S. (2010). Mesenchymal and haematopoietic stem cells form a unique bone marrow niche. Nature *466*, 829–834.

Movasaghi, Z., Rehman, S., and Rehman, I.U. (2007). Raman spectroscopy of biological tissues. Appl. Spectrosc. Rev. *42*, 493–541.

Muraglia, A., Cancedda, R., and Quarto, R. (2000). Clonal mesenchymal progenitors from human bone marrow differentiate in vitro according to a hierarchical model. J. Cell Sci. *113*, 1161–1166.

Nauta, A.J., and Fibbe, W.E. (2007). Immunomodulatory properties of mesenchymal stromal cells. Blood *110*, 3499–3506.

Okamoto, T., Aoyama, T., Nakayama, T., Nakamata, T., Hosaka, T., Nishijo, K., Nakamura, T., Kiyono, T., and Toguchida, J. (2002). Clonal heterogeneity in differentiation potential of immortalized human mesenchymal stem cells. Biochem. Biophys. Res. Commun. *295*, 354–361.

Pinho, S., Lacombe, J., Hanoun, M., Mizoguchi, T., Bruns, I., Kunisaki, Y., and Frenette, P.S. (2013). PDGFRα and CD51 mark human nestin+ sphere-forming mesenchymal stem cells capable of hematopoietic progenitor cell expansion. J. Exp. Med. *210*, 1351–1367.

Pittenger, M.F., Mackay, A.M., Beck, S.C., Jaiswal, R.K., Douglas, R., Mosca, J.D., Moorman, M.A., Simonetti, D.W., Craig, S., and Marshak, D.R. (1999). Multilineage potential of adult human mesenchymal stem cells. Science *284*, 143–147.

Quirici, N., Soligo, D., Bossolasco, P., Servida, F., Lumini, C., and Deliliers, G.L. (2002). Isolation of bone marrow mesenchymal stem cells by anti-nerve growth factor receptor antibodies. Exp. Hematol. *30*, 783–791.

Robey, P.G., Kuznetsov, S.A., Riminucci, M., and Bianco, P. (2014). Bone marrow stromal cell assays: in vitro and in vivo. Methods Mol. Biol. *1130*, 279–293.

Russell, K.C., Phinney, D.G., Lacey, M.R., Barrilleaux, B.L., Meyertholen, K.E., and O'Connor, K.C. (2010). In vitro high-capacity assay to quantify the clonal heterogeneity in trilineage potential of mesenchymal stem cells reveals a complex hierarchy of lineage commitment. Stem Cells *28*, 788–798.

Russell, K.C., Lacey, M.R., Gilliam, J.K., Tucker, H.A., Phinney, D.G., and O'Connor, K.C. (2011). Clonal analysis of the proliferation potential of human bone marrow mesenchymal stem cells as a function of potency. Biotechnol. Bioeng. *108*, 2716–2726.

Sacchetti, B., Funari, A., Michienzi, S., Di Cesare, S., Piersanti, S., Saggio, I., Tagliafico, E., Ferrari, S., Robey, P.G., Riminucci, M., and Bianco, P. (2007). Self-renewing osteoprogenitors in bone marrow sinusoids can organize a hematopoietic microenvironment. Cell *131*, 324–336.

Schulze, H.G., Konorov, S.O., Caron, N.J., Piret, J.M., Blades, M.W., and Turner, R.F. (2010). Assessing differentiation status of human embryonic stem cells noninvasively using Raman microspectroscopy. Anal. Chem. *82*, 5020–5027.

Serakinci, N., Guldberg, P., Burns, J.S., Abdallah, B., Schrødder, H., Jensen, T., and Kassem, M. (2004). Adult human mesenchymal stem cell as a target for neoplastic transformation. Oncogene *23*, 5095–5098.

Simmons, P.J., and Torok-Storb, B. (1991). Identification of stromal cell precursors in human bone marrow by a novel monoclonal antibody, STRO-1. Blood *78*, 55–62.

Zhou, B.O., Yue, R., Murphy, M.M., Peyer, J.G., and Morrison, S.J. (2014). Leptin-receptor-expressing mesenchymal stromal cells represent the main source of bone formed by adult bone marrow. Cell Stem Cell *15*, 154–168.

Zhu, J., Garrett, R., Jung, Y., Zhang, Y., Kim, N., Wang, J., Joe, G.J., Hexner, E., Choi, Y., Taichman, R.S., and Emerson, S.G. (2007). Osteoblasts support B-lymphocyte commitment and differentiation from hematopoietic stem cells. Blood *109*, 3706–3712.

Stem Cell Reports
Supplemental Information

Multiparameter Analysis of Human Bone Marrow Stromal Cells Identifies Distinct Immunomodulatory and Differentiation-Competent Subtypes

Sally James, James Fox, Farinaz Afsari, Jennifer Lee, Sally Clough, Charlotte Knight, James Ashmore, Peter Ashton, Olivier Preham, Martin Hoogduijn, Raquel De Almeida Rocha Ponzoni, Y. Hancock, Mark Coles, and Paul Genever

SUPPLEMENTAL FIGURES

Figure S1

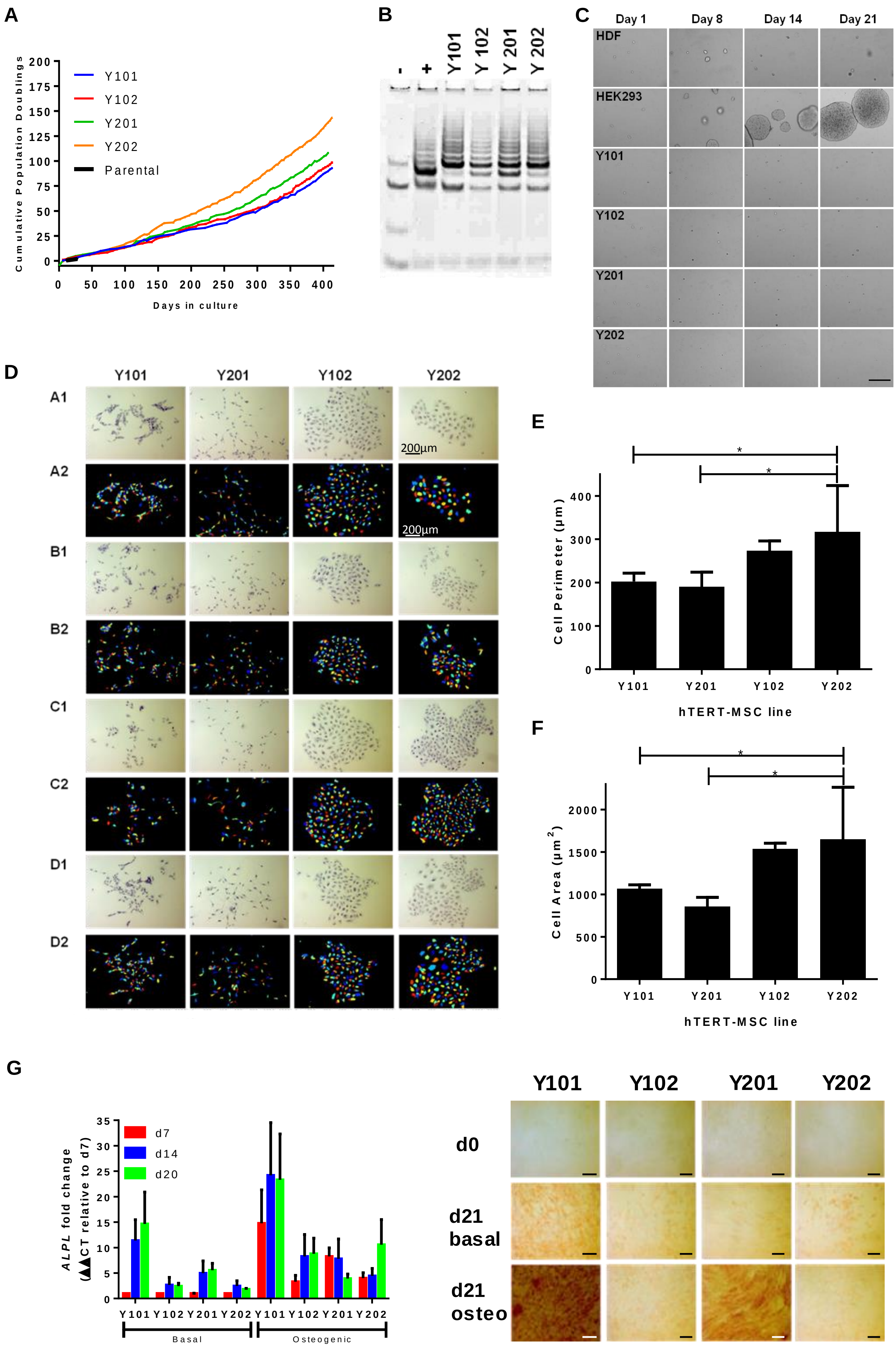

Figure S2

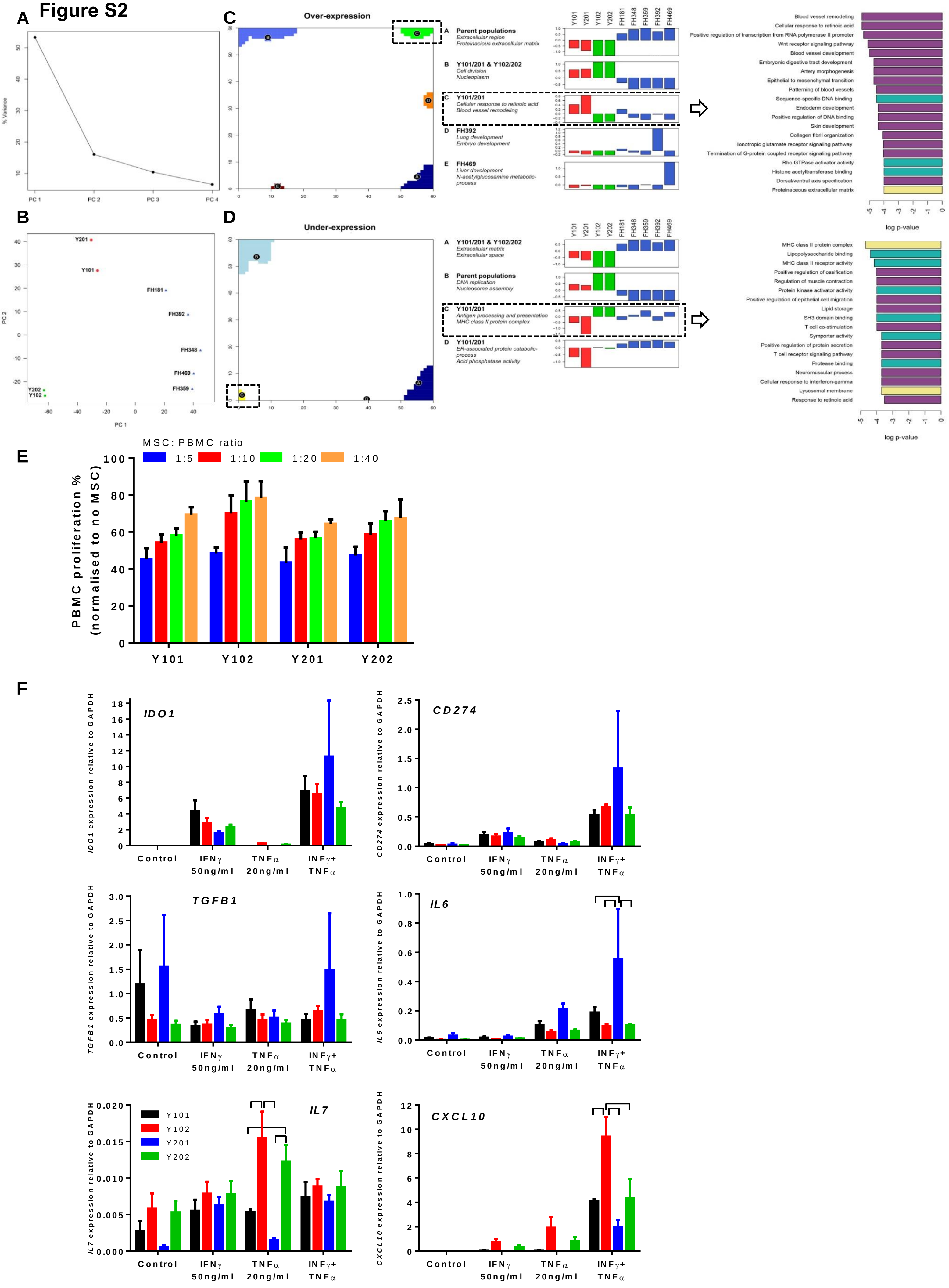
A
% Variance
PC 1
PC 2
PC 3
PC 4
B
Y201
Y101
FH181
FH392
FH348
FH469
FH359
Y202
Y102
PC 1
PC 2
C
Over-expression
A Parent populations
Extracellular region
Proteinacious extracellular matrix
B Y101/201 & Y102/202
Cell division
Nucleoplasm
C Y101/201
Cellular response to retinoic acid
Blood vessel remodeling
D FH392
Lung development
Embryo development
E FH469
Liver development
N-acetylglucosamine metabolic-process
Y101
Y201
Y102
Y202
FH181
FH348
FH359
FH392
FH469
Blood vessel remodeling
Cellular response to retinoic acid
Positive regulation of transcription from RNA polymerase II promoter
Wnt receptor signaling pathway
Blood vessel development
Embryonic digestive tract development
Artery morphogenesis
Epithelial to mesenchymal transition
Patterning of blood vessels
Sequence-specific DNA binding
Endoderm development
Positive regulation of DNA binding
Skin development
Collagen fibril organization
Ionotropic glutamate receptor signaling pathway
Termination of G-protein coupled receptor signaling pathway
Rho GTPase activator activity
Histone acetyltransferase binding
Dorsal/ventral axis specification
Proteinaceous extracellular matrix
log p-value
D
Under-expression
A Y101/201 & Y102/202
Extracellular matrix
Extracellular space
B Parent populations
DNA replication
Nucleosome assembly
C Y101/201
Antigen processing and presentation
MHC class II protein complex
D Y101/201
ER-associated protein catabolic-process
Acid phosphatase activity
MHC class II protein complex
Lipopolysaccharide binding
MHC class II receptor activity
Positive regulation of ossification
Regulation of muscle contraction
Protein kinase activator activity
Positive regulation of epithelial cell migration
Lipid storage
SH3 domain binding
T cell co-stimulation
Symporter activity
Positive regulation of protein secretion
T cell receptor signaling pathway
Protease binding
Neuromuscular process
Cellular response to interferon-gamma
Lysosomal membrane
Response to retinoic acid
log p-value
E
MSC: PBMC ratio
1:5
1:10
1:20
1:40
PBMC proliferation %
(normalised to no MSC)
Y101
Y102
Y201
Y202
F
IDO1
IDO1 expression relative to GAPDH
CD274
CD274 expression relative to GAPDH
TGFB1
TGFB1 expression relative to GAPDH
IL6
IL6 expression relative to GAPDH
IL7
IL7 expression relative to GAPDH
CXCL10
CXCL10 expression relative to GAPDH
Control
IFNγ 50ng/ml
TNFα 20ng/ml
INFγ+ TNFα
Y101
Y102
Y201
Y202

Figure S3

A

Cell/Tissue	Femur	Tibia	Sternum	Calvaria
Central marrow	3.9	3.9	5.2	3.4
Endosteal region	5.4	6.0	8.5	2.2
Articular chondrocyte	60.0	54.0	11.1	nd
Hypertrophic chondrocyte	13.8	3.6	31.0	6.9
Osteocyte	9.5	22.9	nd	7.6

B

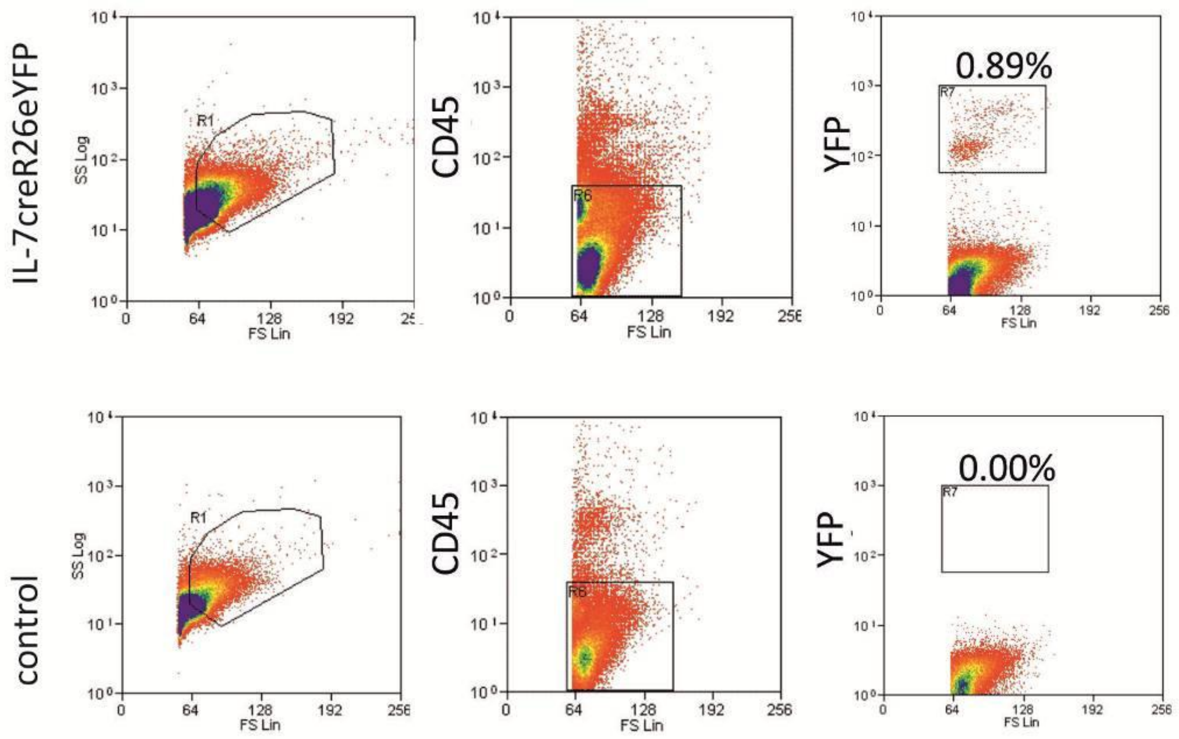

C

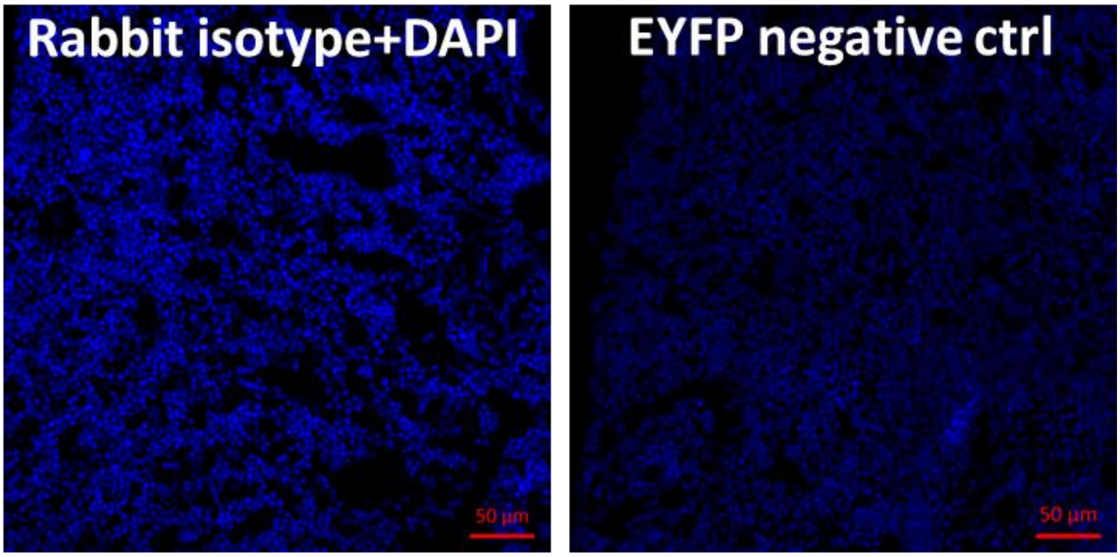

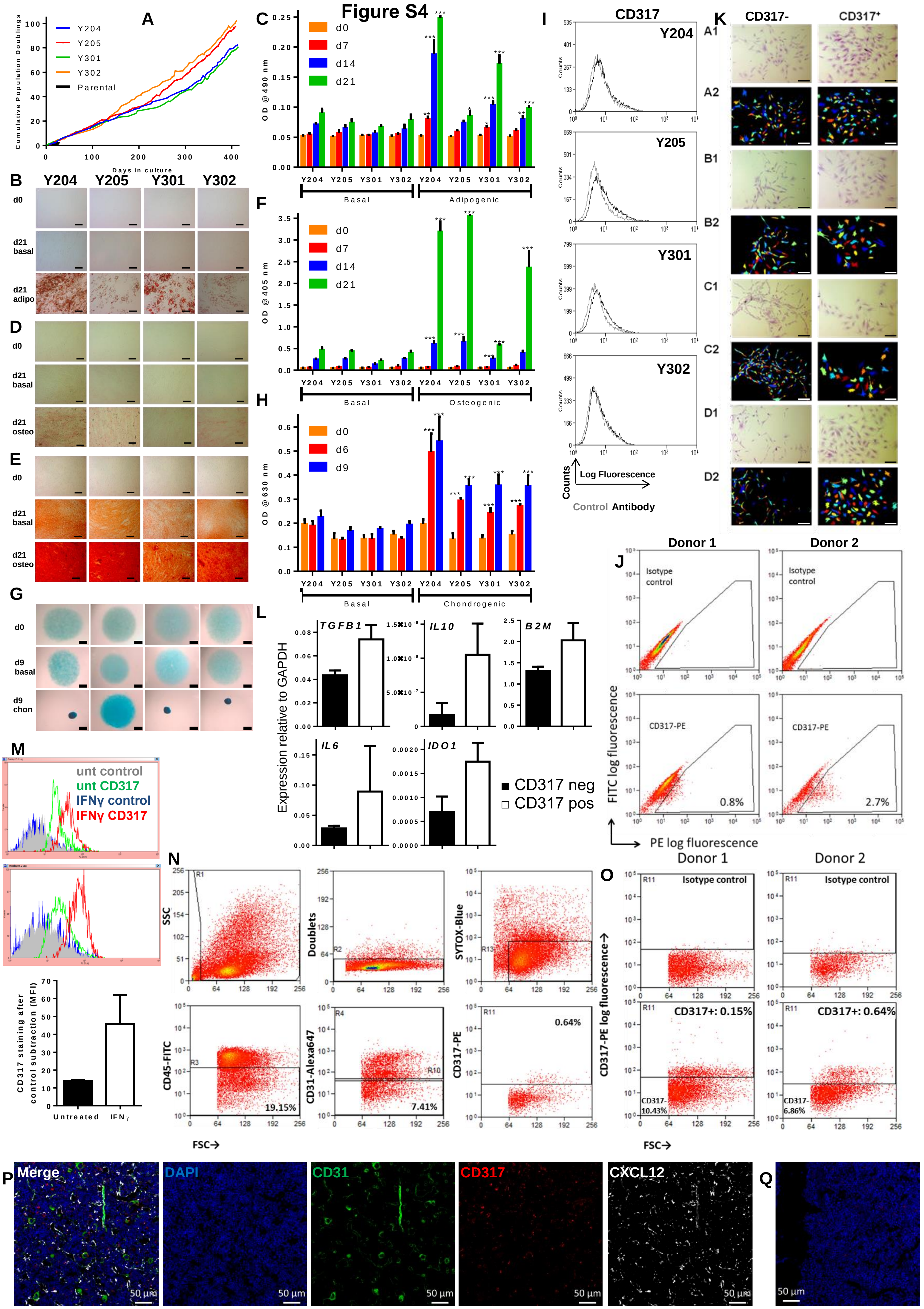
Figure S4
A
Cumulative Population Doublings
Days in culture
Y204
Y205
Y301
Y302
Parental
B
Y204 Y205 Y301 Y302
d0
d21 basal
d21 adipo
C
OD @ 490 nm
d0
d7
d14
d21
Basal
Adipogenic
D
d21 osteo
E
F
OD @ 405 nm
Osteogenic
G
d9 basal
d9 chon
H
OD @ 630 nm
d6
d9
Chondrogenic
I
CD317
Counts
Log Fluorescence
Control Antibody
K
CD317-
CD317+
A1
A2
B1
B2
C1
C2
D1
D2
J
Donor 1
Donor 2
Isotype control
CD317-PE
0.8%
2.7%
FITC log fluorescence
PE log fluorescence
L
TGFB1
IL10
B2M
IL6
IDO1
Expression relative to GAPDH
CD317 neg
CD317 pos
M
unt control
unt CD317
IFNγ control
IFNγ CD317
CD317 staining after control subtraction (MFI)
Untreated
IFNγ
N
SSC
Doublets
SYTOX-Blue
CD45-FITC
CD31-Alexa647
CD317-PE
19.15%
7.41%
0.64%
FSC→
O
Isotype control
CD317+: 0.15%
CD317+: 0.64%
CD317- 10.43%
CD317- 6.86%
CD317-PE log fluorescence→
P
Merge
DAPI
CD31
CD317
CXCL12
50 µm
Q

SUPPLEMENTAL FIGURE LEGENDS

Figure S1. Generation and analysis of hTERT-BMSC clones, Y101, Y102, Y201 and Y202, Related to Figure 1.

(A) Cumulative population doublings of hTERT-BMSC clonal lines with the original parental primary BMSCs.

(B) Telomerase activity measured in the four hTERT-BMSC clones against positive (+) and negative (-) controls. Banding indicates PCR amplification of telomeric repeats.

(C) Anchorage-independent growth assays in soft agarose assays using the hTERT-BMSC clones Y101, Y102, Y201 and Y202, alongside human diploid fibroblast (HDF) and HEK-293 cells as negative/positive controls respectively. Representative images of colonies taken at same magnification are shown after 1, 8, 14 and 21 days of culture, scale bar = 250μm.

(D) Microscopic examination of colonies formed by Y101, Y102, Y201 and Y202 clones. Toluidine blue stain (A1-D1) and CellProfiler analysis (A2-D2), four representative images shown. Images are all taken at same scale, scale bar = 200μm.

(E-F) Cell perimeter (E) and area (F) measurements of hTERT-BMSC clones using CellProfiler software. The mean ± SEM of four independent experiments examining on average 124 cells per experiment is shown. Data were analysed for statistically significant differences in perimeter or area using 1way ANOVA and Newman-Keuls multiple comparison tests between the hTERT –BMSC lines; *=$p<0.05$.

(G) Histological ALP staining and real time qPCR gene expression analysis for *ALPL* of the hTERT-BMSCs cultured in osteogenic differentiation media. Mean gene expression levels ± SEM are shown (average of three experiments performed in triplicate). Scale bar = 200μm.

Figure S2. Bioinformatic and functional analysis of hTERT-BMSC clones, Related to Figure 2.

(A-B) Principal component analysis of Y101, Y102, Y201 and Y202 clones against the parent BMSC population (FH181) and four other primary BMSC cultures (FH392, FH348, FH469 and FH359).

(C-D) Analysis of over- (C) and under- (D) expressed metagenes in Y101, Y102, Y201 and Y202 clones, the parent BMSC population (FH181) and four additional primary BMSC cultures (FH392, FH348, FH469 and FH359).

(E) Peripheral blood mononuclear cell (PBMC) proliferation assay in the presence of hTERT-BMSCs at the stated ratios. Data represents average inhibition of PBMC proliferation ± SEM from five experiments performed in triplicate. 2way ANOVA with Sidak's multiple comparisons between the 4 cell lines under the different conditions were performed, revealing no significant difference at any cell:cell ratio.

(F) Expression of immunomodulatory factors (*IDO1, CD274, TGFB1, IL6, IL7 and CXCL10*) by hTERT-BMSC clones following exposure to TNFα and/or INFγ. Mean expression levels from three experiments performed in triplicate ± SEM are shown. Where there was significant difference in gene expression between control and treated cells, significant differences between the cell lines was assessed by 2way ANOVA and differences are indicated as brackets across the data points at at least $p<0.05$.

Figure S3. Identification of EYFP-positive cells in bone/marrow of IL-7cre Rosa26-EYFP lineage-tracing mice, Related to Figure 3.

(A) Distribution of EYFP-positive cells in bone. Slides were scored for the number of EYFP-positive cells per field as a percentage of total nucleated cells as stained by DAPI, nd = not determined.

(B) Bone marrow cells were isolated from the femora and tibiae of IL-7cre Rosa26-EYFP mice and analysed by flow cytometry. The R1 gate represents live cells separated by FSC/SSC, R6 gate discriminates between CD45-negative, non-hematopoietic cells and CD45-positive cells, R7 gate identifies CD45-negative, EYFP-positive cells. Representative flow cytometric data shown.

(C) Representative isotype control IL-7cre Rosa26-EYFP bone marrow section and bone marrow section showing EYFP negative control stained with the anti-GFP, alexa fluor 488 conjugated antibody. DAPI nuclear stain (blue).

Figure S4. Validation of CD317 expression in BMSC subpopulations, Related to Figure 4.

(A) Cumulative population doublings of four additional hTERT-BMSC clonal lines (Y204, Y205, Y301, Y302) with the original parental primary BMSCs (experiment performed alongside data shown in Figure S1A).

(B-H) Assessment of the OAC potential of Y204, Y205, Y301 and Y302 BMSC clones; representative images (B, D, E, G) and quantification (C, F, H) of cells differentiated with adipogenic (B, C), osteogenic (D, E, F) and chondrogenic (G, H) supplements. Scale bar = 200µm in B, D & E whilst in G, scale bar = 2mm. Data represents average quantified values ± SD for 3 independent experiments performed using 6 replicates. 2way ANOVA with Sidak's multiple comparisons were used to assess statistically significant differences in the three measures of differentiation between basal and induced conditions at each time point for each cell line. Statistical significance is denoted by *=$p<0.05$; **=$p<0.01$; ***=$p<0.001$.

(I) Representative flow cytometric analysis of CD317 expression in Y204, Y205, Y301 and Y302 BMSC clones.

(J) Identification of rare $CD317^+$ cells in heterogeneous primary BMSC cultures by flow cytometry, two example donors shown.

(K) Microscopic examination of colonies formed by $CD317^-$ and $CD317^+$ cells sorted from primary BMSCs. Crystal violet stain (A1-D1) and CellProfiler analysis (A2-D2), four representative images shown. Scale bar represents 200µm

(L) Expression of immunomodulatory factors by $CD317^-$ and $CD317^+$ cells sorted from primary BMSCs by qPCR. Mean ± SEM of gene expression in sorted cells from two donors is shown.

(M) CD317 staining on untreated (unt) primary BMSCs or following treatment with 50ng/ml IFN-γ for 24hrs. Flow cytometry histograms for the two donors (left and middle) are shown and CD317 mean fluorescence intensity (MFI) is quantified (right) following subtraction of the control staining in the adjacent histograms. Mean MFI ± SD of two independent experiments are shown in the histogram.

(N) Gating strategy for flow cytometric sorting of human bone-marrow mononuclear cells (BM-MNCs). Debris, doublets, dead cells (sytox blue positive), CD45-positive and CD31-positive cells were removed. Percentages shown are the cell populations of the live (sytox blue negative) cells. Data

shown are representative of two independent experiments using two separate preparations of primary human BM-MNCs.

(O) Representative flow cytometric analysis of CD317 expression in BM-MNCs: two donors are shown with the percentage of total live cells negative for CD45 and CD31 but expressing CD317 displayed.

(P) Representative staining of mouse bone marrow sections. Scale bar represents 50µm. Blue = nuclear DAPI, green = CD31, red = CD317 and white = *Cxcl12*-DsRed. Single channel and merged overlay images are shown.

(Q) Representative antibody isotype control for the donkey anti-rabbit antibody used as a secondary antibody to reveal CD317 staining on mouse bone marrow sections in Fig 4F/G. Scale bar represents 50µm. Blue = DAPI; Red = isotype control plus donkey anti-rabbit secondary antibody.

SUPPLEMENTAL TABLES

**Target**	**Conjugate**	**Supplier**	**Catalogue number**
hCD45	FITC	eBioscience	11-9459
hCD166	PE	BD Pharmingen	559263
hCD44	FITC	BD Pharmingen	555478
hCD90	None	eBioscience	14-0909
hCD105	APC	eBioscience	17-1057
hCD29	Purified (none)	BD Pharmingen	556048
hCD73	Purified (none)	BD Pharmingen	550256
hCD34	FITC	Miltenyi Biotec	130-081-001
hCD317	PE	BioLegend or eBioscience	348406 or 12-3179
hCD271	FITC	Miltenyi Biotec	130-091-917
hCD51/CD61	None	BD Pharmingen	555504
hPDGFRα	None	Santa Cruz Biotechnology	sc-21789
hCD146/MCAM	None	Abcam	ab24577
hICAM	None	BD Pharmingen	555510
hCD274	APC	eBioscience	17-5983
hCD295	PE	R&D Systems	FAB867P
hCD31	Alexa Fluor 647	BD Pharmingen	558094
hIL-7	None	R&D Systems	MAB207
mCD317	None	Novus Biologicals	NBP2-27154SS
mCD295	Biotin	R&D Systems	BAF497
mPerilipin A/B	None	Bioss	bs-6765R
mIgG2b, isotype control	PE	Biolegend	400313
mIgG1, isotype control	None	eBioscience	14-4714
Goat anti-mouse-IgG	Alexa Fluor 488	Invitrogen	A11001
Goat anti-rabbit IgG	Alexa Fluor 488	Invitrogen	A11008
Donkey anti-rabbit-IgG	Alexa Fluor 647	Invitrogen	A31573
Anti-GFP tag	Alexa Fluor 488	Invitrogen	A21311

Table S1. Antibodies used for flow cytometry. Key: h=human; m=mouse.

Peak assignment cm^{-1}	Peak Identification*
717.1	Lipids
779.3	DNA/RNA nucleic acids
849.8	Proteins/amino acids
966.0	DNA/RNA nucleic acids
999.6	Phenylalanine
1088.6	PO^{-}_{2} stretch DNA
1203.7	Amide III and other amino acids/nucleic acids
1239.4	Amide III
1334.8	Lipids/nucleic acids/proteins
1447.3	Proteins and lipids
1657.8	Lipids

Table S2. Mean Raman peak assignments corresponding to the averaged spectra per cell line (Figure 4H, I); maximum uncertainties in the peak measurements are ± 0.8 cm^{-1}, corresponding to the spectral resolution of the instrument.

**Gene Target**	**Primer sequence**
Peroxisome proliferator activated receptor (*PPAR*)-γ forward	GGCTCCATGACAAGGGAGTTTC
PPARγ reverse	AACTCAAACTTGGGCTCCATAAAG
Lipoprotein lipase (*LPL*) forward	GAGGTACTTTTCAGCCAGGATGTAAC
LPL reverse	AGCTGGTCCACATCTCCAAGTC
Ribosomal protein S (*RPS*)*27a* forward	TGGATGAGAATGGCAAAATTAGTC
RPS27a reverse	CACCCCAGCACCACATTCA
Alkaline Phosphatase (*ALPL*) forward	GGGAACGAGGTCACCTCCAT
ALPL reverse	TGGTCACAATGCCCACAGAT
RUNX2 forward	GGTTAATCTCCGCAGGTCAC
RUNX2 reverse	GTCACTGTGCTGAAGAGGCT
SOX9 forward	TTCCGCGACGTGGACAT
SOX9 reverse	TCAAACTCGTTGACATCGAAGGT

Table S3. qPCR primer sequences

SUPPLEMENTAL EXPERIMENTAL PROCEDURES

Primary BMSC isolation and culture

Primary human BMSCs were isolated from femoral heads obtained with informed consent during routine hip replacement, as previously described (Dyson et al., 2007; Etheridge et al., 2004), or as explant cultures from human tibial plateaus after routine knee replacement surgery. Cells were cultured in DMEM containing 15% FBS, 100U/mL penicillin and 100µg/mL streptomycin, which was changed every 3–4 days; cells were passaged at 70-80% confluency. Cells were expanded and used between passages (p)1 and p5.

hTERT BMSC production

The hTERT lentiviral vector was produced using the ViraPower Lentiviral Gateway Expression Kit (Invitrogen) according to manufacturer's guidelines, and as previously described (Saleh et al., 2012) before transduction into primary human BMSCs from a single donor. After clonal selection, the human telomerase gene was subcloned from pCI-neo-hEST2 (Addgene, courtesy of Robert Weinberg) into the pENTR 1A backbone provided using restriction enzyme digestion and ligation. After appropriate screening, an LR recombination reaction was performed, successfully incorporating the hTERT gene into pLent6/V5-DEST lentiviral vector.

pLenti6/V5-DEST-hTERT lentiviral stocks were produced using 293FT cells according to manufacturer's instructions. 24hrs after lipofectamine transfection of pLenti6/V5-DEST-hTERT and viral packaging mix, culture media was replaced with 5ml fresh culture medium to concentrate viral particles. Lentivirus-containing medium was harvested 48hrs post-transfection, and lentiviral stocks were titered using the Lenti-X qRTPCR Titration Kit (Clontech).

BMSCs were transduced with pLenti6/V5-DEST-hTERT in the presence of 6µg/ml polybrene. After 16hrs the lentivirus-containing medium was removed and replaced with fresh BMSC media. After a further 24hrs, the cells were passaged and 2µg/ml blasticidin was added to the culture media. Media was replaced every 3-4 days with fresh blasticidin-containing medium for 12 days, when no living mock-transfected cells remained. To select single cell lines the transduced BMSCs were trypsinized

and plated in 10cm plates at 10 cells/cm^2. Cells were grown in BMSC medium containing 20% HyClone serum for plating out the cells, then replaced with fresh BMSC medium containing 15% HyClone serum every 3/4 days for 14 days (until discrete single cell colonies were visible). Single cell colonies were isolated using cloning cylinders, silicone grease, and trypsin-EDTA and then transferred to wells of 24-well plates. Once the cells reached 70% confluence they were passaged into 6-well plates, then 25cm^2 flasks, and finally 75cm^2 flasks for continued expansion. Cells were counted at each passage to determine population doubling time and maintained for continued culture in DMEM containing 10% FBS and antibiotics. Telomerase activity in hTERT-BMSCs was assessed using the TRAPeze® gel-based telomerase detection kit (Millipore) according to manufacturer’s guidelines. Analyses using CellProfiler software were performed according to the designer instructions (www.cellprofiler.org).

I*n vitro* hTERT BMSC differentiation

In vitro induction of BMSC differentiation was performed as previously outlined (Etheridge et al., 2004; Hoogduijn et al., 2006; Saleh et al., 2011). Osteogenic differentiation was induced for up to 21 days with the addition of 5mM β-glycerophosphate, 50□g/mL L-ascorbic acid phosphate and 10nM dexamethasone to growth media. Osteogenesis was assessed by both histological staining with alizarin red and quantitative elution of the stain alongside quantitative assays for alkaline phosphatase (ALP) activity (Cook et al., 2014; Hoogduijn et al., 2006) and gene expression analyses for Runx2 and ALP. Adipogenic differentiation was induced with 0.5mM isobutyl-methylxanthine, 1µg/ml insulin, 100µM indomethacin and 1µM dexamethasone for up to 21 days. Lipid accumulation was examined and quantified by Oil Red O staining (Cook et al., 2014) and gene expression analyses for *PPARγ* and *LPL* were performed. Micromass pellet cultures were utilized for chondrogenesis assays. Briefly, cells were trypsinized and resuspended to $2x10^7$ cells/ml in serum-free media; 20µl cell suspension was placed in the middle of a culture well and allowed to adhere at 37°C for 3h. Wells were flooded with serum-free media containing 50µg/ml L-ascorbic acid phosphate, 100nM dexamethasone, 40µg/ml L-proline, 1% ITS^+, with or without TGF-β; medium was changed twice

weekly. Chondrogenic differentiation was assessed using 9 day micromass cultures and potential was indicated through an early cell condensation phenotype (Johnson et al., 2012). After 1, 6 or 9 days of culture, cells were fixed in 100% methanol (-20°C, 30 mins), then incubated overnight with 0.5% alcian blue 8GS in 1M HCl (pH 0.2 to selectively stain highly sulphated glycosaminoglycans (GAGs) associated with cartilage tissue). Cells were washed extensively with distilled water before stereomicroscope visualisation. Specific GAG-associated stain was eluted from pellets by 6M guanidine incubation for 3 hours with rocking; supernatant absorbance was measured at 630nm. Total GAG content (pellet associated and secreted) for each cell line following chondrogenic induction was measured by the Blyscan glycosaminoglycan assay (Biocolor Ltd, UK) according to the manufacturer's instructions. Gene expression analyses by qPCR for the early chondrogenic marker Sox9 were also performed.

Flow cytometry and cell sorting

Cell surface marker profiles were assessed on a CyAn flow cytometer and analyzed using Summit software (v4.3; Beckman Coulter). Cells were detached from culture plastic by incubation with PBS containing 0.2% BSA and 5mM EDTA, and stained with either directly conjugated primary antibodies, or successive incubations with optimised concentrations of primary antibody and an appropriate fluorescent-conjugated secondary antibody. All antibodies are listed in Table S1. For cell sorting, BMSCs were processed and stained as above then sorted on a MoFlo Astrios high-speed, sterile cell sorter (Beckman Coulter). Cryopreserved, primary human bone marrow mononuclear cells (BM-MNCs) were purchased from Lonza (Belgium); both healthy donors were male Caucasians, donor one was 21 years of age, donor two was 22. Cells were thawed according to the Poietics protocol, rested for an hour before staining for 40mins with optimised concentrations of CD45, CD31 and CD317 antibodies or equivalent concentrations of isotype controls before sorting. Cells were also extracted from femora and tibiae bone marrow of IL-7cre Rosa26-EYFP mice and incubated with ACK buffer to lyse any red blood cells prior to flow cytometric analysis. CD317 staining was also

assessed on primary cells after 50ng/ml recombinant human IFN-γ (PeproTech EC Ltd, UK) treatment for 24 hours.

Anchorage independent growth assay

Anchorage independent growth assays as an assessment of tumorigenic potential were performed on hTERT BMSC-lines alongside HEK-293 cells as positive controls and human dermal fibroblasts as a negative control. Cells were seeded into media containing 0.45% agarose in plates coated with 0.75% agarose and cultured for up to 21 days before imaging by brightfield microscopy.

RNA isolation and gene expression analysis

Total RNA was isolated from 80-90% confluent cultures using Macherey-Nagel Nucleospin RNA II kit. RNA quality was determined with the Agilent 2100 Bioanalyzer nano chip; high quality RNA samples were labelled using Agilent One Color Quick Amp labelling. Samples were processed for genome-wide gene expression analysis using the Agilent SurePrint G3 Human Gene Expression 8x60K v2 Microarray kit; triplicate arrays were performed on each cell line. All quality control checks met required standards. Data were analyzed using GeneSpring version 12.1 software (Agilent Technologies), using initial one-way ANOVA with controlling for multiple testing. This allowed for p-value adjustment lowering false discovery rate whilst maintaining sensitivity. Genes with a minimum two-fold difference in expression level and an adjusted p-value of <0.05 were considered differentially expressed. Subsequent pathway analysis was performed on lists of differentially expressed genes within the GeneSpring software. Additional analyses were performed using the statistical programming language R and the Bioconductor package limma.

Quantitative real-time PCR (qPCR) was performed using two protocols; the first used 500ng cDNA with the StepOnePlus™ Real-Time PCR System, TaqMan Universal PCR Master Mix and the assay-on-demand primer/probes for *IDO1* (Hs00158027.m1), *IL6* (Hs00174131.m1), IL7 (Hs00174202.m1), *IL10* (Hs00174086.m1), *TGFB1* (Hs00171257.m1), *CD274* (Hs00204257.m1), *CXCL10* (Hs00171042.m1) and *B2M* (Hs00187842.m1) (all Applied Biosciences, Foster City, CA,

USA). Glyceraldehyde 3-phosphate dehydrogenase (GAPDH) mRNA served as endogenous control for normalization (Hs99999905.m1; Applied Biosciences). Intra- and inter-assay variations were determined by negative controls and positive reference samples. Changes in target gene expression relative to GAPDH were quantified using the comparative CT method. The second method used the SYBR green system and optimised amounts of cDNA reverse transcribed using superscript II (Invitrogen, UK) from 1µg of RNA isolated from primary BMSCs differentiated along the osteogenic, adipogenic or chondrogenic lineages using Trizol/chloroform extraction (Life Technologies, UK). cDNA was amplified using Fast SYBR Green Master Mix (Life Technologies) and optimised primers (Table S3) for analysis of adipogenic genes (peroxisome proliferator-activated receptor gamma and lipoprotein lipase), osteogenic genes (alkaline phosphatase and *RUNX2*) or a chondrogenic gene (*SOX9*) using the StepOne real-time PCR system. Gene expression levels were quantified using the comparative CT (ΔΔCt) method and StepOne software v2.3 relative to the expression of the housekeeping gene RPS27a and normalized to d7 basal levels.

Agilent microarray data processing

Preprocessing of the microarray data was carried out using the statistical programming language R and the Bioconductor package limma. Firstly, background correction with optimal offset value was applied to the arrays to minimize signal related to non-specific fluorophore binding. The optimal offset value was evaluated by identifying which value stabilized the variances of probeset intensities the most. Quantile normalization was then applied to the arrays, which resulted in each array having the same empirical distribution of probeset intensity values, removing systematic variation from the data and scaling it so comparisons could be made between them. The values were then transformed by $\log_2$ to control for variability in both highly and poorly expressed genes. Uninformative data such as control probesets and genes with low variance were also removed to decrease the false discovery rate (FDR). Lastly, the average expression levels from each of the replicates were collected into a global expression dataset.

Microarray data can be found on ArrayExpress: E-MTAB-3511 (release date: 03-August-2015).

Differential gene expression analysis

Differentially expressed genes were defined as those which exhibited a log fold-change of greater than two. The statistical significance of this differential expression was evaluated using a moderated t-test, known as Empirical Bayes. Instead of calculating an estimate of the variance for each gene, this moderated version calculates a global variance from the variances for all genes in the dataset. This is combined with an average of the variance for each gene producing a statistical test value that has a t-distribution. The Bioconductor package limma was used to calculate this log fold-change and significance value by applying a linear model to the probe set intensities for each gene using a least squares method. Limma also employed the Benjamini and Hochberg algorithm to control for the effects of multiple testing. This procedure allowed for the adjustment of the p-values and the lowering of the false discovery rate whilst keeping the sensitivity for true positive results. Genes with a two-fold difference in expression levels and with an adjusted p-value of ≤0.05 between any pair of cell lines in the dataset were considered as significantly differentially expressed. The differentially expressed genes limma outputs were then collected into a differential expression dataset for use in subsequent analyses.

Sample and gene clustering algorithms

A preliminary insight into the variation of gene expression profiles between cell lines was achieved by performing principal component analysis (PCA) on the differential expression dataset using the R CRAN package stats. This approach reduces the dimensionality of the dataset into two orthogonal variables, visualised as a two-dimensional scatterplot of the cell lines. Cell lines that cluster close together in two-dimensional space are considered to have more similar expression profiles than those further away.

SOM-mapping of global expression profiles

Self-organizing map (SOM) machine learning was applied to the global expression dataset using the R CRAN package oposSOM (Wirth et al., 2011). This unsupervised learning method uses neural networks to reduce high dimensional data onto a two-dimensional mosaic. Each tile on the mosaic

represents a metagene consisting of mini-clusters of genes with similar expression profiles. Genes are assigned to clusters using the minimum Euclidian distance and metagenes with similar expression profiles are positioned next to each other. Because the same metagene is assigned to the same tile in SOMs across all samples, direct visual comparisons of global expression can be made and areas of notable over- or under- expression are readily observed. After running the analysis, oposSOM outputs a SOM for each cell line, a combined SOM of over- and under- expressed metagene hotspots, including a list of genes which contribute to those metagenes and their relative expression in each cell line.

Gene set over-representation analysis

Gene set over-representation analysis was used to help define the functional nature of the respective spots. For each of the spots the degree of over-representation of 1454 pre-defined gene sets was measured using the hypergeometrical distribution (Wirth et al., 2011). This assigns the probability that members of a certain gene set are present in a list of genes compared against the random chance of their appearance independent of expression values. Gene sets contain lists of genes which have been shown to act in the same pathway or appear to be coexpressed. An over-representation p-value is then used to evaluate the significance of over-representation of each gene set to each metagene. The hypergeometrical distribution then ranks the list of gene sets by decreasing significance of over-representation for the spots. In addition, gene ontologies were used to describe the attributes of each gene product in these hotspots. Gene ontologies provide a comprehensive and unified way of annotating gene products with i) the biological process to which they contribute ii) their functional biochemical activity iii) the cellular component where they are active (Ashburner et al., 2000). Using a hypergeometrical distribution, the statistical over-representation of these terms with regards to the global expressed set was also calculated. Again, oposSOM was used to carry out this analysis, and given the row names of the expression dataset are labelled using ENSEMBL gene IDs is automatically performed when the function is called.

Immunofluorescent staining for IL-7

To identify IL-7 immunopositivity in hTERT-BMSCs, $2x10^4$ cells were seeded onto coverslips, allowed to adhere overnight then fixed in 4% PFA for 5mins at 21°C before permeabilization with 0.1% Triton X-100 in PBS and blocking of endogenous peroxidase activity with 1% H_2O_2. All future steps were performed at room temperature in a humidified chamber in the presence of 1% blocking reagent supplied with the tyramide signal amplification kit. Extensive washes were performed between antibodies; all reagents were from Invitrogen unless otherwise stated. Samples were initially blocked for 30mins in PBS containing 1% blocking reagent before incubations for 60mins with optimised concentrations of either mouse anti-human IL-7 primary antibody (R&D clone 7417/catalogue number MAB207) or an IgG1 isotype control. Coverslips were incubated with 1:200 biotinylated anti-mouse secondary antibody for 30mins before incubation with 1:100 diluted horseradish peroxidase for 45mins. Alexa Fluor-647 conjugated tyramide was 1:100 diluted in amplification buffer and incubated with the samples for 5mins. Finally, samples were extensively washed before mounting in Prolong gold containing DAPI and imaged using an LSM710 invert confocal microscope (Carl Zeiss).

***In vivo* IL-7 lineage tracing**

IL-7cre Rosa26-EYFP mice were bred under pathogen free conditions at the University of York (Repass et al., 2009). All work was in accordance with ethical approvals from the University of York and Home Office License 60/4169. For lineage tracing experiments, tissues from IL-7cre Rosa26-EYFP mice were fixed in 4% PFA overnight and placed at room temperature in 30% sucrose solution for 15mins followed by 15% sucrose overnight at 4°C. Tissues were then coated with 10% polyvinyl alcohol (PVA) and frozen in cooled isopropanol on dry ice. They were then fully mounted in 10% PVA and sectioned (at10□m) using a Bright's cryostat, slide-mounted and observed by fluorescence microscopy.

Immunohistochemistry

Bone samples from the femurs of 11- or 12-week old female hybrid C57BL/Ka x C57BL/6 mice (derived from in-house crossing of *Cxcl12*$^{tm2.1Sjm}$/J (The Jackson Laboratory) and wild-type C57BL/6 mice) or IL-7cre Rosa26-EYFP and non-EYFP expressing mice were used. These mice express DsRedE2 under control of the *Cxcl12* promoter (Ding and Morrison, 2013) or IL-7 EYFP, respectively. Samples were fixed overnight at 4°C in 4% paraformaldehyde/75mM lysine/10mM periodate fixative. Bones were decalcified for 3 days in 10% EDTA, 0.1M Tris, pH7.2 at 4°C with agitation before overnight incubation in 30% sucrose in PBS at 4°C and embedding in optimal cutting temperature embedding media (Tissue-Tek). 5µm sections were cut, applied to slides and blocked for 1hr at room temperature in staining buffer (PBS + 0.05% BSA + 5% species specific serum) before staining with optimised concentrations of primary antibodies in staining buffer at 4°C overnight. Three five-minute washes (PBS + 0.05% BSA) were performed before slides were incubated with optimised concentrations of relevant secondary antibody for 1hr at RT. Where indicated, anti-GFP tag antibody was applied for 1hr. Three further washes, DAPI counterstaining and prolong gold mounting (Invitrogen) were performed before imaging using an LSM710, LSM780 or LSM 510 confocal microscopes (Carl Zeiss) and image analysis with Zen 2012 (blue edition) software (Carl Zeiss).

ELISA for IL-7 secretion

Cells were seeded into 6 well plates at $2x10^4$ cells/cm^2 and left to adhere overnight. Culture media was replaced with 2ml fresh media; 24h later the quantity of IL-7 in the supernatant was measured using the Quantikine HS Human IL-7 Immunoassay (R&D systems) according to the manufacturer's guidelines, and compared against medium alone.

Peripheral blood mononuclear cell (PBMC) proliferation assay

PBMCs were incubated with the hTERT-BMSCs at ratios of 1:5, 1:10, 1:20 and 1:40. PBMC proliferation was quantified by detection of ^{3}H-thymidine incorporation into the PBMCs after three days of stimulation with anti-CD3 anti-CD28 antibodies.

Raman Methods

The hTERT BMSCs and CD317+ cells were seeded at $5x10^4$ cells per CaF_2 microscope slide; slides were incubated in growth media with 0.1% amphotericin B for 24h before replacement with DMEM containing 0.5% FBS and another 24h incubation for G_0 synchronisation. Before Raman analysis, slides were PBS rinsed twice and air-dried. Raman spectra were collected using a HORIBA XploRA micro-Raman instrument with 532nm laser wavelength and x100 objective (NA = 0.9) in confocal setting. Spectra were acquired ambiently using 45s laser exposure resulting in 3.5mW power at the sample. Each measurement was obtained using a 2400 lines/mm diffraction grating, with the results averaged over 2 spectral acquisitions over the 'finger-print' range ($600cm^{-1}$ to $1800cm^{-1}$). Raman spectra were collected from hTERT BMSC lines using 20 randomly selected cells, with five randomly selected points taken in the nucleus of each cell. The resulting 100 spectra per BMSC line were baseline-corrected and averaged so that a representative spectrum of each BMSC population was obtained. For the CD317+ cell line, a Raman spatial map consisting of 172 spectra across the nucleus was acquired. These spectra were baseline-corrected and averaged to obtain a representative spectrum of the individual CD317+ cell under investigation. To prevent laser-induced cell damage, Raman spectra were monitored during real-time data acquisition, with optical inspection also being performed after each Raman measurement. All spectra were collected using the LabSpec 5 software (HORIBA). Baseline-correction was performed to each spectrum using an interval linear fitting method obtained in the '*Raman tool set'* software (Candeloro et al., 2013). Further analysis on the averaged spectra, including individual peak baselines and Gaussian peak fitting, was conducted using IGOR Pro 6.32. Raman markers were determined from the set of two-peak intensity ratios derived from key peaks in the averaged cell spectra.

Data Analysis

Data were analysed in GraphPad Prism v6.05; statistical analyses using 2way ANOVA followed by Sidak's multiple comparisons, paired t-test or 1way ANOVA with relevant multiple comparison test were used, where indicated.

SUPPLEMENTAL REFERENCES

Ashburner, M., Ball, C.A., Blake, J.A., Botstein, D., Butler, H., Cherry, J.M., Davis, A.P., Dolinski, K., Dwight, S.S., Eppig, J.T., *et al.* (2000). Gene Ontology: Tool for the Unification of Biology. The Gene Ontology Consortium. Nature genetics. *25*, 25-29.

Candeloro, P., Grande, E., Raimondo, R., Di Mascolo, D., Gentile, F., Coluccio, M.L., Perozziello, G., Malara, N., Francardi, M., and Di Fabrizio, E. (2013). Raman Database of Amino Acids Solutions: A Critical Study of Extended Multiplicative Signal Correction. The Analyst. *138*, 7331-7340.

Cook, D.A., Fellgett, S.W., Pownall, M.E., O'Shea, P.J., and Genever, P.G. (2014). Wnt-Dependent Osteogenic Commitment of Bone Marrow Stromal Cells Using a Novel Gsk3beta Inhibitor. Stem cell research. *12*, 415-427.

Ding, L., and Morrison, S.J. (2013). Haematopoietic Stem Cells and Early Lymphoid Progenitors Occupy Distinct Bone Marrow Niches. Nature. *495*, 231-235.

Dyson, J.A., Genever, P.G., Dalgarno, K.W., and Wood, D.J. (2007). Development of Custom-Built Bone Scaffolds Using Mesenchymal Stem Cells and Apatite-Wollastonite Glass-Ceramics. Tissue engineering. *13*, 2891-2901.

Etheridge, S.L., Spencer, G.J., Heath, D.J., and Genever, P.G. (2004). Expression Profiling and Functional Analysis of Wnt Signaling Mechanisms in Mesenchymal Stem Cells. Stem cells. *22*, 849-860.

Hoogduijn, M.J., Gorjup, E., and Genever, P.G. (2006). Comparative Characterization of Hair Follicle Dermal Stem Cells and Bone Marrow Mesenchymal Stem Cells. Stem cells and development. *15*, 49-60.

Johnson, K., Zhu, S., Tremblay, M.S., Payette, J.N., Wang, J., Bouchez, L.C., Meeusen, S., Althage, A., Cho, C.Y., Wu, X., *et al.* (2012). A Stem Cell-Based Approach to Cartilage Repair. Science. *336*, 717-721.

Repass, J.F., Laurent, M.N., Carter, C., Reizis, B., Bedford, M.T., Cardenas, K., Narang, P., Coles, M., and Richie, E.R. (2009). Il7-Hcd25 and Il7-Cre Bac Transgenic Mouse Lines: New Tools for Analysis of Il-7 Expressing Cells. Genesis. *47*, 281-287.

Saleh, F.A., Frith, J.E., Lee, J.A., and Genever, P.G. (2012). Three-Dimensional in Vitro Culture Techniques for Mesenchymal Stem Cells. Methods in molecular biology. *916*, 31-45.

Saleh, F.A., Whyte, M., Ashton, P., and Genever, P.G. (2011). Regulation of Mesenchymal Stem Cell Activity by Endothelial Cells. Stem cells and development. *20*, 391-403.

Wirth, H., Loffler, M., von Bergen, M., and Binder, H. (2011). Expression Cartography of Human Tissues Using Self Organizing Maps. BMC bioinformatics. *12*, 306.
